# Tangent space alignment: Transfer learning for Brain-Computer Interface

**DOI:** 10.3389/fnhum.2022.1049985

**Published:** 2022-12-02

**Authors:** Alexandre Bleuzé, Jérémie Mattout, Marco Congedo

**Affiliations:** ^1^GIPSA-Lab, University Grenoble Alpes, CNRS, Grenoble INP, Grenoble, France; ^2^Lyon Neuroscience Research Center, INSERM, CNRS, University Claude Bernard Lyon 1, Lyon, France

**Keywords:** Brain-Computer Interface, Riemannian geometry, transfer learning, domain adaptation, ERP, motor imagery, SSVEP

## Abstract

Statistical variability of electroencephalography (EEG) between subjects and between sessions is a common problem faced in the field of Brain-Computer Interface (BCI). Such variability prevents the usage of pre-trained machine learning models and requires the use of a calibration for every new session. This paper presents a new transfer learning (TL) method that deals with this variability. This method aims to reduce calibration time and even improve accuracy of BCI systems by aligning EEG data from one subject to the other in the tangent space of the positive definite matrices Riemannian manifold. We tested the method on 18 BCI databases comprising a total of 349 subjects pertaining to three BCI paradigms, namely, event related potentials (ERP), motor imagery (MI), and steady state visually evoked potentials (SSVEP). We employ a support vector classifier for feature classification. The results demonstrate a significant improvement of classification accuracy, as compared to a classical training-test pipeline, in the case of the ERP paradigm, whereas for both the MI and SSVEP paradigm no deterioration of performance is observed. A global 2.7% accuracy improvement is obtained compared to a previously published Riemannian method, Riemannian Procrustes Analysis (RPA). Interestingly, tangent space alignment has an intrinsic ability to deal with transfer learning for sets of data that have different number of channels, naturally applying to inter-dataset transfer learning.

## 1. Introduction

A Brain-Computer Interface (BCI) is a system that allows interactions between a human and a machine using only neurophysiological signals coming from the brain. It aims at rehabilitating, improving, or enhancing the ability of the user by means of a computerized system (Wolpaw et al., 2002). The most common modality used to record neurophysiological signals is electroencephalography (EEG). This is mainly because EEG is affordable, completely safe for the user and because it features a high temporal resolution. EEG signals can be translated into a command to be sent to a computer by means of a decoding algorithm. The loop is often closed by means of a feedback given to the user.

Several BCI applications have emerged to help patients, such as spellers (Yin et al., 2015; Rezeika et al., 2018) or wheelchair controllers (Li et al., 2013). The focus in this line of research is to restore lost communication or movement capabilities. Other applications are designed for the rehabilitation of patients after an incapacitating event such as a stroke (Frisoli et al., 2012; Mane et al., 2020). Non-clinical applications have also been proposed, for example to provide a means of control in video games (Congedo et al., 2011; Bonnet et al., 2013). Mixed approaches are also possible, for example in the Cybathlon BCI Race, where people with complete or severe loss of motor function compete in a video game-based competition (Perdikis et al., 2018).

Several paradigms can be used in order to control a BCI. The most commons are event related potentials (ERP), motor imagery (MI), and steady state visually evoked potentials (SSVEP). The ERP paradigm consists of electrical potentials evoked by sensory stimulations; in MI the user imagines to move body parts, resulting in synchronizations and desynchronizations in the sensory-motor cortex; in SSVEP-based BCIs, the user concentrates on visual stimuli flashed at distinct frequencies, leading to responses at the same frequency in the brain. Regardless of the paradigm, it is necessary to calibrate the BCI system in order to allow proper decoding. The calibration process is time consuming, annoying for the healthy user and problematic for the clinical population, which has limited mental resources (Mayaud et al., 2016). In fact, a calibration is required not only for every new user, but also for every new session of the same user. This is due to the high inter-subject and inter-session variability of the features extracted from the EEG. Such variability is caused by several factors, including, but not limited to, the impedance and placement of EEG electrodes, individual morphological and physiological characteristics of the brain and changing brain states.

One way do deal with this variability is to use transfer learning (TL). This means trying to reuse some of the information we have already gathered on known data that may be coming from either previous subjects or previous sessions. In transfer learning we usually consider two types of data. The **source** represents the data we already know on a given subject whereas the **target** consists of a new subject whose some training data may be available, but mostly is unlabeled and is to be used as a test. The aim is to adapt as accurately as possible the data of the target using the few available training data to the source data (or vice versa). In order to do so, several methods have already been developed.

The authors in Jayaram et al. (2016) adapt the weights given to spatial features that are meant to predict the stimulus in order to transfer information from one subject to another or from one session to another. Some other methods adapt the parameters of a neural network. For example the authors in Fahimi et al. (2019) perform a partial retraining of a deep neural network on a small number of samples of a new user, improving significantly the accuracy. Unsupervised domain adaptation methods have also emerged, as in Sun et al. (2015), where the authors perform unsupervised transfer learning in the Euclidean domain, using covariance matrices to align data from different subjects. A well-established approach for classification in the BCI field is to use covariance matrices of the signal since those matrices have many relevant properties (Congedo et al., 2017). The covariance matrices are Symmetric Positive Definite (SPD) and therefore lie in a Riemannian manifold. In this way, some algorithms have been developed to achieve transfer learning in the Riemannian manifold of SPD matrices. For instance, the authors in Zanini et al. (2018) propose a recentering procedure consisting in translating the center of mass of both the source and target data to the identity using parallel transport. This procedure is actually equivalent to a whitening using the Riemannian mean as anchor point. In Yair et al. (2019), both the center of mass of the source and target data are translated to their midpoint along the geodesic, allowing equivalent results. The authors of Rodrigues et al. (2019), inspired by the Procrustes analysis, proposed to add two more steps after recentering: a stretching of the observations, so as to equalize the dispersion of the data in the source and target domain and a rotation, so as to align as much as possible the center of mass of each class between the source and the target data set. The method, named Riemannian Procrustes Analysis (RPA), was shown to allow efficient transfer learning. A later alignment method was discussed in He and Wu (2020). This method is similar to Sun et al. (2015) with improvement related to enhanced dimensions in the Euclidean space. The authors of Zhang et al. (2020) chose another approach by transferring instances of the source close enough to the target in order to enhance the low data availability of the target model. They used MI data and compared the proximity of source and target trials using Hamming distance after preprocessing steps. Another idea proposed in Zhang and Wu (2020) is to find a common subspace between source and target, yielding a projection matrix to reduce the gap between the source and the target. Finally the authors train on the source subspace to test on target subspace.

In this article we introduce a Riemannian transfer learning approach similar in spirit to the RPA approach (Rodrigues et al., 2019), but operating in the tangent space. Our contribution has multiple benefits as compared to previous attempts. First, it lies in a state-of-the-art BCI feature space, the Riemannian tangent space, introduced in the BCI domain by Barachant et al. (2010). Since the tangent space is an Euclidean space, there exists a wide variety of well-established tools to decode the data therein and in general they are faster as compared to a decoding approach in the Riemannian manifold. Second, since it acts on an Euclidean space, it can be used for all kind of feature vectors, not just those obtained in a Riemannian setting. Third, our method is computationally effective, as it only requires one singular value decomposition (SVD). Fourth, it extends naturally to the heterogeneous transfer learning case, i.e., when the number and/or placements of electrodes is not the same in the source and target data set. In a similar previous attempt the SVD has been applied independently on the source and target dataset and the resulting matrices are then used to align the data (Sun et al., 2015). Our method is instead casted as a Procrustes problem and therefore it fulfills a well-known optimality condition for inter-domain alignment.

A previous version of our method has been presented in Bleuze et al. (2021). As compared to that presentation, we have improved it by adding several ways to deal with the rank deficiency of the cross product matrix. Also, here we test it on a very large amount of data, namely, 18 BCI databases comprising a total of 349 subjects. Furthermore, these databases pertain to three BCI modalities: event related potentials (ERP), motor imagery (MI), and steady state visually evoked potentials (SSVEP). Therefore, the present study is a comprehensive test bed, which is a well-grounded way to reach general conclusions when comparing machine learning pipelines.

## 2. Materials and methods

### 2.1. Notations

Throughout this article we will denote matrices with upper case bold characters (**A**), vectors with lower case bold characters (**a**), indices and scalars by lower case italic characters (*a*), and constants by upper case italics (*A*). The function *tr*(.) will indicate the trace of a matrix, (.)^*T*^ its transpose, ||.|| the 2-norm or the Frobenius norm, ° the Hadamard product, log(.), and exp(.) the matrix logarithm and exponential, respectively. **I**_*N*_ will denote the identity matrix in dimension *N*.

### 2.2. Riemannian geometry

Let us consider a set of trials {_**X**_*n*_}*n*∈[1, *N*]_ with shape (*N*_*c*_, *N*_*sample*_), where *N*_*c*_ is the number of channels, *N*_*sample*_ the number of (temporal) samples and *N* the number of matrices in the set. A generic trial is simply denoted as **X**. In order to be as close as possible to a realistic scenario, we consider data with a low level of pre-processing and we do not use any artifact removal method, such as ocular artifacts or outliers removal (Çınar and Acır, 2017; Minguillon et al., 2017).

The (spatial) sample covariance matrix estimation (SCM) writes


(1) $$\begin{array}{l} \text{C}=\frac{1}{N_{sample}-1}\text{X}{\text{X}}^{T}. \end{array}$$

The SCM has shape (*N*_*c*_, *N*_*c*_). It lies in a Riemannian manifold of symmetric positive definite (SPD) matrices (Bhatia, 2009). It is therefore possible to classify directly a set {**C**_*n*_} of covariance matrices by means of classification algorithms acting on such a manifold, such as the Minimum Distance to Riemannian Mean (MDRM) classifier (Barachant et al., 2012) or its refinement Riemannian Minimum Distance to Means Field (RMDMF; Congedo et al., 2019). It is also possible to project the matrices onto the tangent space of the manifold at a base point **M** and use Euclidean classifiers therein (Barachant et al., 2012, 2013). The base point **M** in this work will always be chosen as the Log-Euclidean mean, which is defined as Fillard et al. (2005).


(2) $$\begin{array}{l} \text{M}=\exp\left(\frac{1}{N}\underset{n}{\sum }\log\left({\text{C}}_{n}\right)\right). \end{array}$$

The projection onto the tangent space at base point **M** is obtained by the logarithmic map operator (Nielsen and Bhatia, 2013).


(3) $$\begin{array}{l} {\mathrm{Log}}_{\text{M}}\left(\text{C}\right)={\text{M}}^{\frac{1}{2}}\log\left({\text{M}}^{-\frac{1}{2}}\text{C}{\text{M}}^{-\frac{1}{2}}\right){\text{M}}^{\frac{1}{2}}. \end{array}$$

The projected matrix is now a (*N*_*c*_, *N*_*c*_) symmetric matrix. Since we are concerned with transfer learning (TL), we are interested in matching the position of the source and target data sets in the manifold as much as possible. Following Zanini et al. (2018), we recenter both the source and target data sets by setting their global mean at the identity. This is simply obtained by transforming all trials of a dataset such as


(4) $$\begin{array}{l} C_{rec}=M^{-\frac{1}{2}}CM^{-\frac{1}{2}}, \end{array}$$

where **M** is the center of mass of the observations and **C_rec_** denotes the recentered trial. After recentering all trials their center of mass becomes the identity matrix, corresponding to the “zero” point in an Euclidean space.

The logarithmic mapping at the identity simplifies, yielding


$$\begin{array}{l} {\text{Log}}_{I_{N_{c}}}\left(C_{rec}\right)=I_{N_{c}}^{1/2}\log\left(I_{N_{c}}^{-1/2}M^{-\frac{1}{2}}CM^{-\frac{1}{2}}I_{N_{c}}^{-1/2}\right)I_{N_{c}}^{1/2}\\ \;=\log\left(M^{-\frac{1}{2}}CM^{-\frac{1}{2}}\right). \end{array}$$

The above recentering followed by tangent space projection was first proposed in the BCI field in Barachant et al. (2012, 2013) and is nowadays a standard processing procedure, which in this article is carried out systematically, unless explicitly mentioned.

Once projected onto the tangent space the matrices are vectorized. Since they are symmetric, only the upper (or lower) triangle of the matrix is kept and the off-diagonal terms are weighted by $\sqrt{2}$ so as to preserve the norm of the original matrix. In mathematical notation, the vectorization of tangent vector **S** reads


(5) $$\begin{array}{l} \text{s}=\mathrm{triu}\left(\text{S}\;\circ \;\text{A}\right), \end{array}$$

with triu(.) the operator vectorizing the upper triangle and **A** a matrix with the same shape as **S**, filled with 1 on the diagonal and $\sqrt{2}$ on the off-diagonal part. Since the matrices have been previously recentered, the resulting vectors are also recentered, that is, the mean tangent vector is the zero vector.

Having obtained the tangent vectors as described here above, it is possible to use all the well know classification algorithms that act in an Euclidean space, the most commonly employed in the BCI community being the linear discriminant analysis (LDA; Barachant et al., 2012), support vector classifier (SVC; Xu et al., 2021) and Lasso logistic regression (LR) (Tomioka et al., 2006). In this study, we use the SVC.

### 2.3. Alignment

As anticipated in the introduction, the method here proposed has been inspired by previous Riemannian TL methods, such as in Zanini et al. (2018) and Rodrigues et al. (2019) which focus on covariance matrices in the Riemannian space of SPD matrices. In Rodrigues et al. (2019) the authors consider again the recentering and add two further alignment steps:

1. a *rescaling* so as to match the dispersion around the mean (center of mass) in both the source and target data sets and
2. a *rotation* so as to align the mean of each class as much as possible. This effectively results in a Riemannian Procrustes alignment.

In this article, the same steps are undertaken in the tangent space. In particular, we rotate the tangent vectors using an Euclidean Procrustes procedure.

Let us consider the set of centered tangent vectors for the source {**s**_*n*_}*n*∈[1, *N*_*s*_] and the target {**t**_*n*_}*n*∈[1, *N*_*t*_] domain. *N*_*s*_ and *N*_*t*_ are the number of vectors for, respectively, the source and the target data set. As we will see, in the following it will not be required that the source and target tangent vectors have the same dimensions. Denoting **s** and **t** the generic source and target tangent vectors, the rescaling is obtained setting the average norm within each set to 1, which is readily obtained by transformations


(6) $$\begin{array}{l} \tilde{\text{s}}=\frac{\text{s}}{\frac{1}{N_{s}}\underset{n}{\sum }||{\text{s}}_{n}||} \end{array}$$

and


(7) $$\begin{array}{l} \tilde{\text{t}}=\frac{\text{t}}{\frac{1}{N_{t}}\underset{n}{\sum }||{\text{t}}_{n}||}, \end{array}$$

yielding rescaled source and target data sets ${\left\{{\tilde{\text{s}}}_{n}\right\}}_{n\in \left[1,N_{s}\right]}$ and ${\left\{{\tilde{\text{t}}}_{n}\right\}}_{n\in \left[1,N_{t}\right]}$. It is also possible to set the norm of the target data set equal to the norm of the source data set if it is sought not to modify the norm of the source data set.

For the rotation (alignment), we propose a supervised method that uses the mean point of the classes. Let us consider *K* classes that we ought to align. Although other procedures are possible, in the following we always align the target set to the source set. Let **y** and **z** be the label vectors of, respectively, ${\left\{{\tilde{\text{s}}}_{n}\right\}}_{n\in \left[1,N_{s}\right]}$ and ${\left\{{\tilde{\text{t}}}_{n}\right\}}_{n\in \left[1,N_{t}\right]}$ with shape *N*_*s*_ and *N*_*t*_. We start by computing the mean for each class *k*, given its *N*_*k*_ trials


(8) $$\begin{array}{l} {\bar{\text{s}}}_{k}=\frac{1}{N_{k}}\underset{{\text{y}}_{i}=k}{\sum }{\tilde{\text{s}}}_{i} \end{array}$$

for the source set and


(9) $$\begin{array}{l} {\bar{\text{t}}}_{k}=\frac{1}{N_{k}}\underset{{\text{z}}_{i}=k}{\sum }{\tilde{\text{t}}}_{i} \end{array}$$

for the target set. In the supervised procedure these vectors are the *anchor points* we use for alignment. Therefore, we define


(10) $$\begin{array}{l} \bar{\text{S}}=\left[{\bar{\text{s}}}_{k},k\in \left[1,K\right]\right] \end{array}$$

and


(11) $$\begin{array}{l} \bar{\text{T}}=\left[{\bar{\text{t}}}_{k},k\in \left[1,K\right]\right] \end{array}$$

as the two matrices of shape $\left(\frac{N_{c}\left(N_{c}+1\right)}{2},K\right)$ holding the anchor vectors stacked one next to the other. We can now define the cross-product matrix


(12) $$\begin{array}{l} C_{st}=\bar{S}{\bar{T}}^{T}. \end{array}$$

of shape $\left(\frac{N_{c}\left(N_{c}+1\right)}{2},\frac{N_{c}\left(N_{c}+1\right)}{2}\right)$. Like any rectangular matrix—or squared when source and target have the same number of channels—**C_st_** can be decomposed by singular value decomposition, such that


(13) $$\begin{array}{l} C_{st}=UDV^{T}, \end{array}$$

with **U** and **V** the two orthonormal matrices holding in columns the left and right singular vectors, respectively, and **D** a diagonal matrix holding the singular values. As usual in signal processing, we will retain a subset of the singular vectors in order to suppress noise. Such a truncation has also the advantage to work for the case where **U** and **V** do not have the same shape. As a general rule, we seek the smallest number *N*_*v*_ of singular vectors which corresponding singular values explain at least 99.9% of the variance, resulting in $\tilde{\text{U}}$ and $\tilde{\text{V}}$ with shape $\left(\frac{N_{c}\left(N_{c}+1\right)}{2},N_{v}\right)$. Finally, we are able to align the target vectors previously created ${\left\{{\tilde{\text{t}}}_{n}\right\}}_{n\in \left[1,N\right]}$ to the domain of the source vectors ${\left\{{\tilde{\text{s}}}_{n}\right\}}_{n\in \left[1,N\right]}$ as


(14) $$\begin{array}{l} \hat{\text{t}}=\tilde{\text{U}}{\tilde{\text{V}}}^{T}\tilde{\text{t}} \end{array}$$

where $\hat{\text{t}}$ denotes the aligned target vectors. The newly created set ${\left\{{\hat{\text{t}}}_{n}\right\}}_{n\in \left[1,N\right]}$ is now aligned to the space of source vectors ${\left\{{\tilde{\text{s}}}_{n}\right\}}_{n\in \left[1,N\right]}$, therefore it can be classified with algorithms trained on the source domain. As it is well-known, when the cross-product in Equation (12) is full-rank, the unique solution to the Procrustes optimization problem


(15) $$\begin{array}{l} \underset{\text{Z}}{\text{arg min}}\left(\left||\text{Z}\bar{\text{T}}-\bar{\text{S}}|\right|\right) \end{array}$$

is indeed **Z** = **UV**^*T*^. In our case, the solution is not unique. Note that a connection between this problem and the Bures-Wasserstein metric has been recently described in Bhatia et al. (2019).

**Figure 1:**
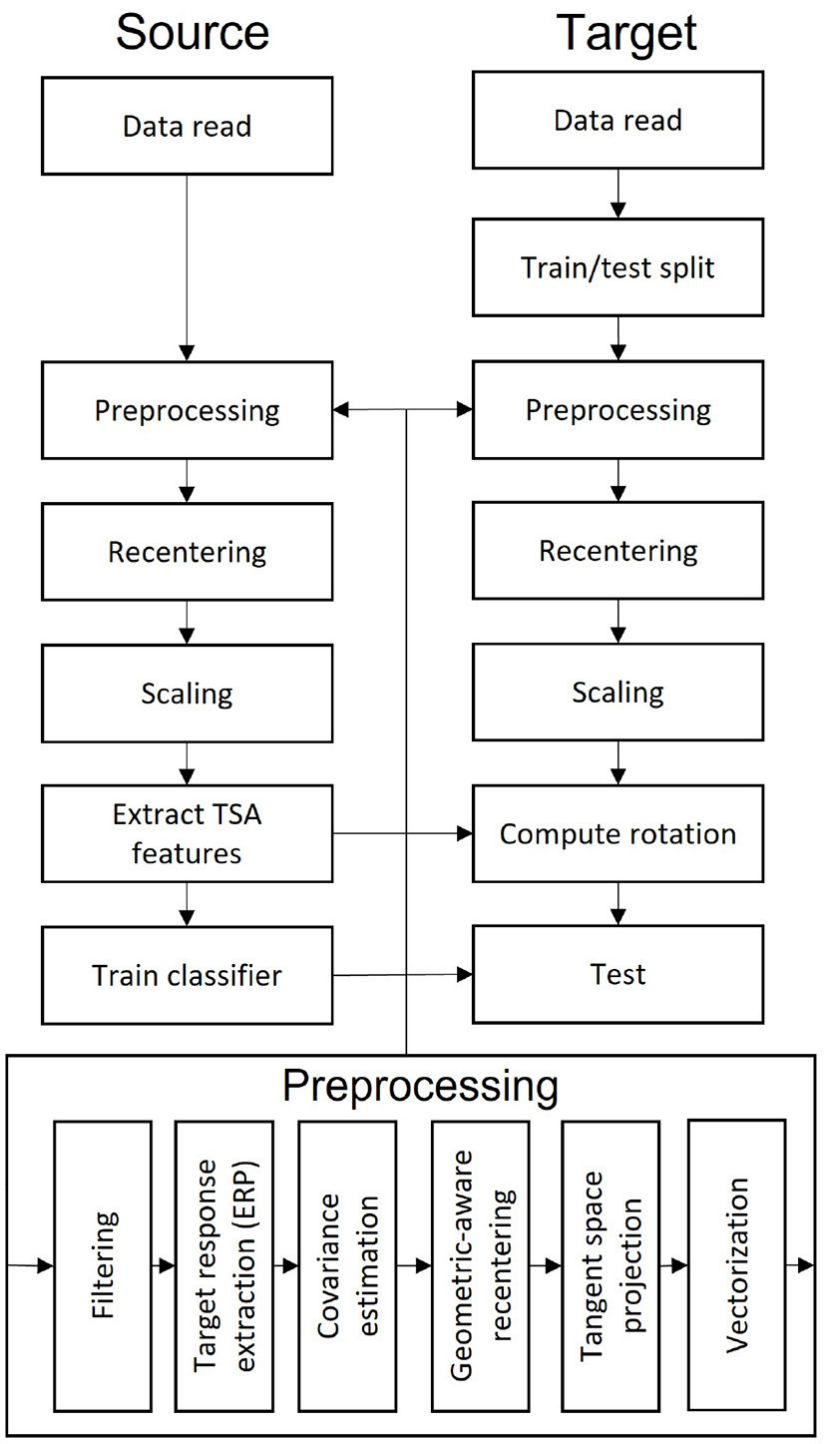
Flowchart summarizing the analysis pipeline.

The whole process for our method is summarized in Figure 1.

### 2.4. Augmenting/improving **C_st_**

Cross-product matrix **C_st_** is usually rank deficient and its estimation could be improved in several ways. In this section, we will suggest two such improvements. First, as long as a supervised TL is possible, since we are relying on averaging the tangent vectors, it is possible to employ robust average estimators. For instance, we may consider the trimmed means, the median or power means to estimate suitable anchor points. We may also stack several of these average estimators to obtain larger matrices ${\bar{\text{s}}}_{k}$ and ${\bar{\text{t}}}_{k}$, which may provide more robust information on the actual central tendency of the data.

**Figure 2:**
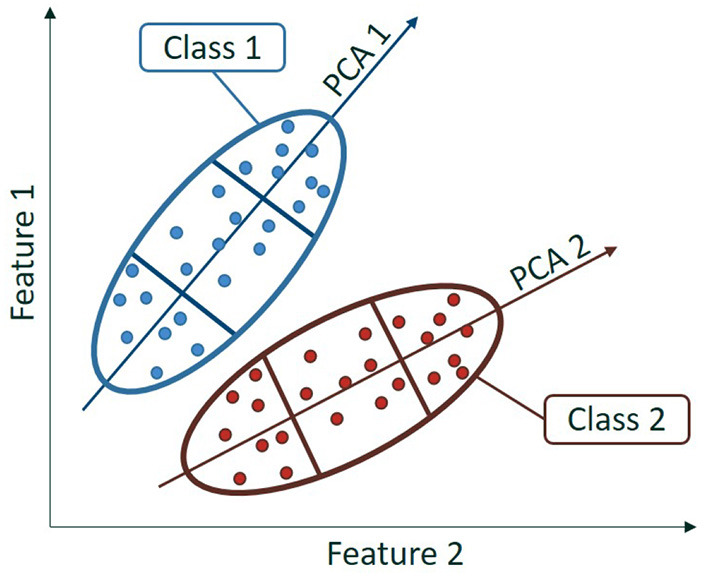
Schematic of a two-class dataset using the first dimension of a PCA for each class.

Second, we cluster the data sets in several subsets describing the shape of the data set when considered altogether and compute separate means for each cluster. We may use, for instance, principal component analysis (PCA) on each class independently to create clusters, as depicted in Figure 2. The centroid of those clusters are then computed and used as anchor points. In order to obtain the clustering for both the source and target data set, we consider for each class a PCA trained on the source and used as such on both the source and target data set.

Using such a clustering procedure, if the source and the target data set display a rather similar shape, their alignment will be very effective, leading to promising transfer learning results. Such a procedure is also possible with unlabeled data in case of unsupervised TL. However, in this case, we have noticed that at least two PCA components are necessary to obtain an efficient transfer learning. Therefore, in the unsupervised case we recommend using at least two PCA components and separate data for each dimensions, creating a **C_st_** matrix with shape $\left[\frac{N_{c}\left(N_{c}+1\right)}{2},N_{d}\times N_{g}\right]$ with *N*_*d*_ the number of dimensions used and *N*_*g*_ the number of groups created in each dimensions. An effective strategy is to visualize the data and their representations in order to verify whether the chosen reduced dimensionality offers a good approximation of the data as it may be as well totally inaccurate, depending on the data, especially for unsupervised TL. For our results, we chose to create three PCA clusters for each class and use these means to compute the cross-product matrix **C_st_** as it gives enough information without reducing the size of the data used for each mean too much.

## 3. Results

The TSA algorithm previously introduced has been tested on three well know BCI paradigms: ERP, MI, and SSVEP. We have analyzed 18 open-access BCI databases available on the Mother Of All BCI Benchmark (MOABB; Jayaram and Barachant, 2018). Python library, of which five uses the ERP paradigm, 10 the MI paradigm, and 3 the SSVEP paradigm. The 18 databases include a total of 349 subjects with very high variability between and within datasets. We summarize the data in Table 1, following Congedo et al. (2019).

**Table 1:** List of the processed databases for event-related potentials, motor imagery paradigms, and steady-state visual evoked potentials.

	**Type**	**Ch**	**Trials**	**Sess**	**Sub**
2003–2015	P300	8	1,800	1	10
2008–2014	P300	8	4,200	1	8
2009–2014	P300	16	1,728	3	10
Brain Invaders 2013a	P300	16	480	1	24
EPFL P300 dataset	P300	32	3,268	4	8
2001–2014	MI	22	288	2	9
2001–2015	MI	13	400	2	12
2002–2014	MI	15	160	1	14
2004–2014	MI	3	720	5	9
Alexandre motor imagery	MI	16	40	1	8
Cho 2017	MI	64	200	1	49
Grosse-Wentrup 2009	MI	128	300	1	10
Physionet motor imagery	MI	64	45	1	109
Weibo 2014	MI	60	160	1	10
Zhou 2016	MI	14	290	3	4
SSVEP exoskeleton	SSVEP	8	160	1	12
SSVEP Nakanishi	SSVEP	8	180	1	9
SSVEP Wang	SSVEP	62	240	1	34

Ch, number of channels; Sess, number of sessions; Sub, number of subjects.

We execute transfer learning from one subject to the other for all possible source/target pair of subjects within each database. The accuracy is evaluated using balanced accuracy since the number of trials per class is often unbalanced (always for the ERP paradigm).

**Figure 3:**
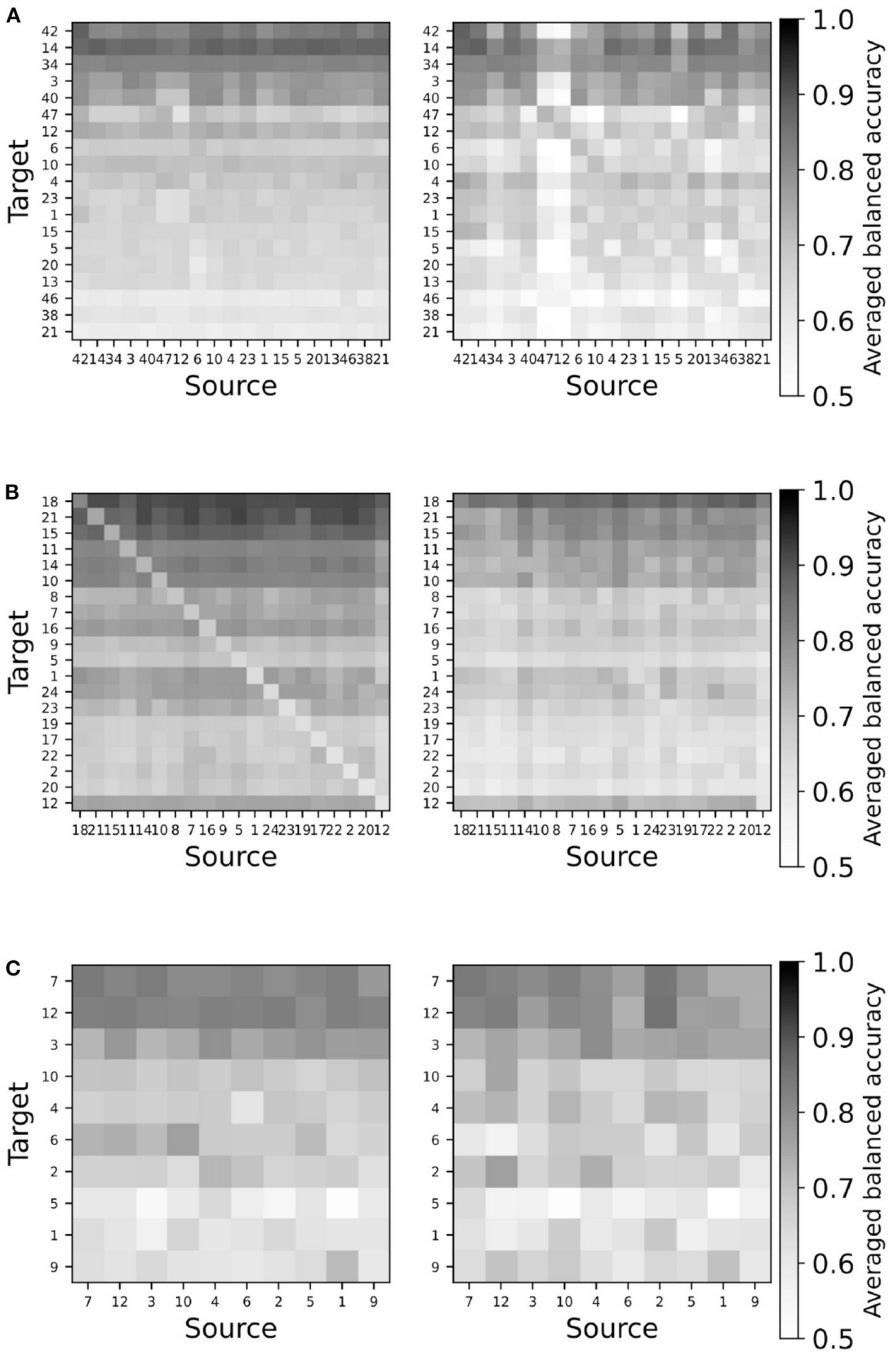
Representative seriation plots for TSA (left) and RPA (right) methods for each paradigms. See text for details. **(A)** Database Cho2017 (MI). **(B)** Database brain invaders 2013a (ERP). **(C)** Database SSVEP exoskeleton (SSVEP).

Since the amount of data of all pair-wise comparisons is huge, we start by visually evaluating all balanced accuracies obtained for a given database by means of seriation plots, i.e., plots showing the accuracy for each pair of target and source subject arranged in matrix form. The accuracy is averaged over all numbers of alignment trials for each pair. The case where the target and the source are the same, i.e., on the diagonal, is replaced by the classical train-test cross-validation accuracy, offering a straightforward benchmark. Furthermore, the target and source subjects are sorted on rows by descending order of the train-test cross-validation accuracy. It should be kept in mind that since the train-test procedure is fully supervised and optimized for the train data, it is expected to outperform a transfer learning method. Figure 3 shows a representative seriation plot for each paradigm allowing the visual comparison of the performance of the TSA vs. the RPA transfer learning method; all figures are available as Supplementary material.

**Figure 4:**
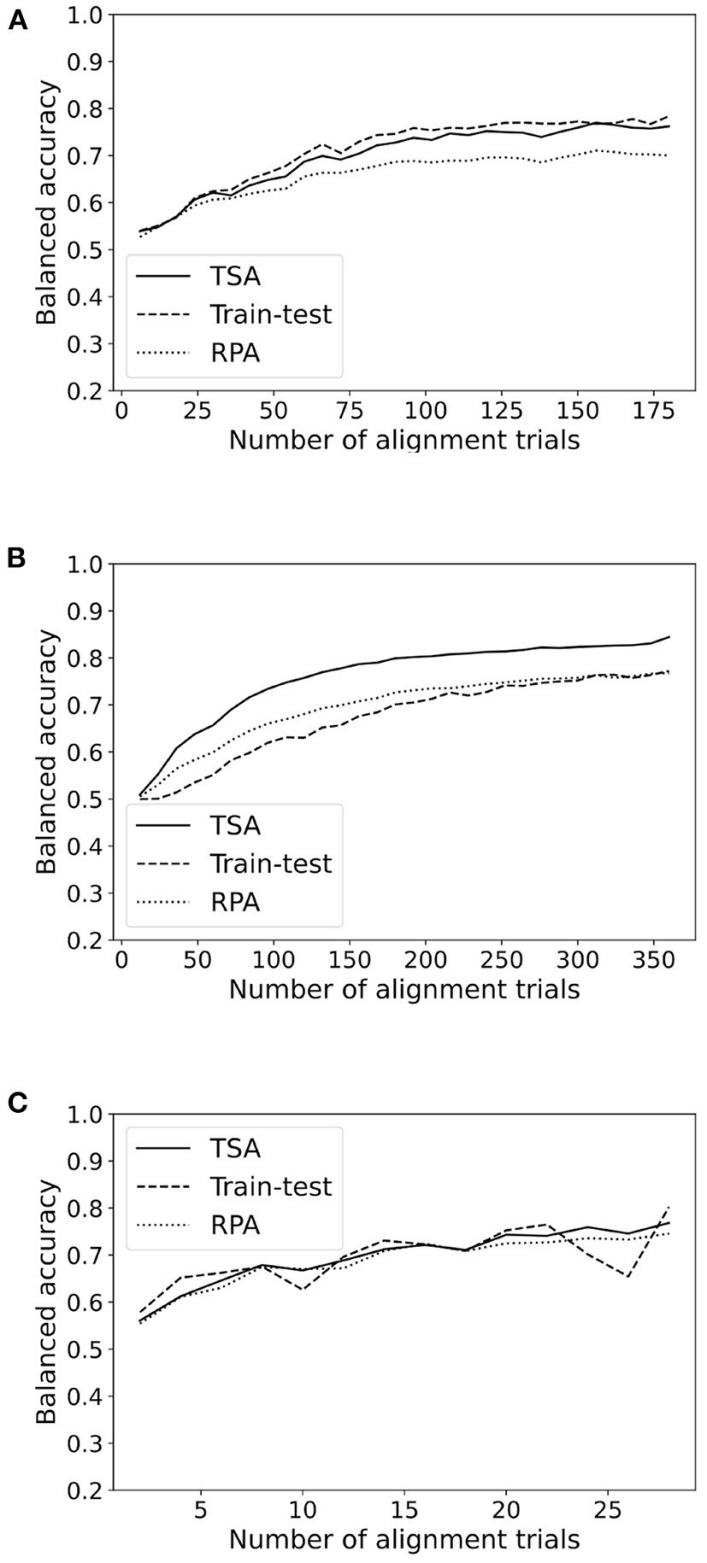
Accuracy as a function of the number of alignment trials for TSA and RPA methods for a representative database in each paradigm (MI, ERP, SSVEP). **(A)** Database Cho2017 (MI). **(B)** Database brain invaders 2013a (ERP). **(C)** Database SSVEP exoskeleton (SSVEP).

In order to evaluate the average performance, we plot the balanced accuracy averaged across all subjects in a database for each method as a function of the number of alignment trials. Since we are averaging across subjects, for this analysis we include only those subjects featuring at least 60% accuracy in a classical train-test cross-validation. This restriction excludes about half of the subjects, leaving 178 subject out of 349. It's important to note that subjects with an overall 60% accuracy usually have more than 70% accuracy with all available training trials. Figure 4 shows a representative plot for each paradigm. All figures are available as Supplementary material.

Then, we summarize all the pair-wise accuracy information in the accuracy tables such as Table 2. These tables give for each target subject the accuracy averaged over numbers of alignment trials and source subjects. For this database, there are 30 numbers of alignment trials considered and 18 possible source subjects, which makes an average over 540 values. This makes the standard error low in general. Accuracy tables for all databases are given as Supplementary material.

**Table 2:** Balanced accuracy ± standard error for the good subjects of database Cho 2017 averaged over number of alignment trials and source for train-test, TSA, and RPA.

	**Train-test acc**	**TSA acc**	**RPA acc**
Subject 1	67.86 ± 2.00	66.70 ± 0.43	65.84 ± 0.43
Subject 3	81.39 ± 0.81	77.78 ± 0.18	74.96 ± 0.32
Subject 4	69.26 ± 1.49	69.24 ± 0.40	69.25 ± 0.50
Subject 5	65.90 ± 2.12	65.03 ± 0.50	59.12 ± 0.55
Subject 6	70.74 ± 2.11	67.65 ± 0.38	59.80 ± 0.53
Subject 10	70.55 ± 1.91	70.71 ± 0.45	62.32 ± 0.43
Subject 12	70.97 ± 1.61	73.01 ± 0.39	67.68 *pm*0.29
Subject 13	63.60 ± 1.14	64.30 ± 0.25	60.83 ± 0.25
Subject 14	88.26 ± 1.52	86.99 ± 0.37	82.06 ± 0.45
Subject 15	66.15 ± 1.64	65.63 ± 0.38	65.20 ± 0.37
Subject 20	64.96 ± 1.13	64.64 ± 0.26	60.79 ± 0.33
Subject 21	60.63 ± 1.50	58.63 ± 0.24	56.54 ± 0.30
Subject 23	68.64 ± 1.62	66.69 ± 0.37	64.94 ± 0.41
Subject 34	82.73 ± 0.37	81.89 ± 0.09	79.97 ± 0.15
Subject 38	61.98 ± 1.27	61.51 ± 0.27	58.53 ± 0.35
Subject 40	76.73 ± 1.89	76.30 ± 0.44	72.21 ± 0.47
Subject 42	88.34 ± 3.32	83.02 ± 0.73	75.78 ± 0.77
Subject 46	62.55 ± 1.79	59.08 ± 0.37	54.64 ± 0.26
Subject 47	72.34 ± 1.66	67.98 ± 0.36	63.03 ± 0.42

The accuracy tables confirm what can be evaluated visually in the average accuracy plots and seriation plots; on the average there is about 1% difference between classical train-test cross-validation accuracy and TSA and about 5% between classical train-test cross-validation and RPA. This speaks in favor of a clear improvement of the TSA method over the RPA method. Table 3 summarizes all the balanced accuracy for each dataset. On the average across databases there is no loss of accuracy using a TSA as compared to the optimal train-test accuracy. This is not true for the RPA.

**Table 3:** Balanced accuracy ± standard error for the good subjects of each dataset averaged over number of alignment trials, target and source for train-test, TSA, and RPA.

	**Train-test acc**	**TSA acc**	**RPA acc**
2003–2015	69.04 ± 0.74	76.05 ± 0.57	69.63 ± 0.57
2008–2014	67.52 ± 0.69	72.00 ± 0.38	65.50 ± 0.38
2009–2014	70.48 ± 0.50	77.38 ± 0.21	70.90 ± 0.21
Brain invaders 2013a	67.26 ± 0.45	76.07 ± 0.11	69.59 ± 0.11
EPFL P300 dataset	67.22 ± 0.61	73.013 ± 0.24	65.28 ± 0.24
2001–2015	81.13 ± 0.73	79.01 ± 0.24	76.21 ± 0.24
2002–2014	78.40 ± 0.70	76.51 ± 0.20	72.55 ± 0.55
2004–2014	74.35 ± 1.30	76.00 ± 0.59	75.27 ± 0.59
Alexandre motor imagery	78.51 ± 2.82	76.30 ± 1.33	75.93 ± 1.33
Cho 2017	71.24 ± 0.52	69.83 ± 0.12	65.97 ± 0.12
Grosse-Wentrup 2009	77.20 ± 0.91	73.12 ± 0.34	72.16 ± 0.34
Physionet motor imagery	70.75 ± 1.35	64.28 ± 0.27	63.26 ± 0.27
Weibo 2014	73.97 ± 1.25	69.66 ± 0.50	71.27 ± 0.50
Zhou 2016	82.82 ± 1.59	82.43 ± 0.86	81.31 ± 0.86
SSVEP exoskeleton	69.50 ± 1.16	69.69 ± 0.40	68.72 ± 0.40
SSVEP Nakanishi	95.55 ± 0.82	95.95 ± 0.28	96.83 ± 0.28
SSVEP Wang	68.26 ± 2.63	57.42 ± 0.76	59.11 ± 0.76
Global	74.42 ± 0.30	74.49 ± 0.12	71.79 ± 0.12

Finally, we performed statistical tests on all pair-wise source/target accuracy results we have collected. To this end, we follow the procedure introduced in Rodrigues et al. (2021). In a nutshell: we first compute signed paired *t*-test for every target subject comparing the accuracy between methods, yielding T statistics *T*_*m, i*_ and *p*-values *p*_*m, i*_ for each pair of methods *m* and target subject *i*. In order to correct for the multiplicity of statistical tests we use Holm's sequential rejection multiple test procedure (Holm, 1979) for each target subject. This produces tables such as Table 4. Corresponding tables for each database are available as Supplementary material. Then, we combine the *p*-values we obtain using the Stouffer's Z-score method (Zaykin, 2011) for each database, yielding multiple *p*-values corresponding to each pair of methods for each database. Those *p*-values are also corrected by means of Holm's procedure and are summarized in Table 5. In this table we can see that among the 18 databases we have analyzed, 11 show a significant improvement of TSA as compared to RPA. No significant difference between the classical train-test cross-validation accuracy and TSA is found with the exception of two databases, for which TSA proves inferior. This number grows to five databases comparing the classical train-test cross-validation accuracy and the accuracy obtained by RPA. Finally, using Stouffer's Z-score method, *p*-values corresponding to each paradigm are computed and corrected with Holm's procedure (Table 6).

**Table 4:** Subject-wise *p*-values for MI database Cho 2017.

	**TSA>RPA**	**TRAIN>TSA**	**TRAIN>RPA**
Subject 1	0.002^*^	0.818	0.420
Subject 3	0.033^*^	< 0.001^*^	< 0.001^*^
Subject 4	0.005^*^	0.690	0.248
Subject 5	< 0.001^*^	0.980	0.301
Subject 6	0.001^*^	0.379	0.013^*^
Subject 10	< 0.001^*^	0.503	0.007^*^
Subject 12	< 0.001	0.086	< 0.001^*^
Subject 13	0.001^*^	0.997	0.700
Subject 14	0.443	< 0.001^*^	< 0.001^*^
Subject 15	0.004^*^	0.964	0.843
Subject 20	0.385	0.991	0.988
Subject 21	< 0.001^*^	1.000	0.999
Subject 23	0.009^*^	0.836	0.514
Subject 34	< 0.001^*^	< 0.001^*^	< 0.001^*^
Subject 38	< 0.001^*^	1.000	0.980
Subject 40	0.020^*^	0.002^*^	< 0.001^*^
Subject 42	0.361	< 0.001^*^	< 0.001^*^
Subject 46	0.126	1.000	0.995
Subject 47	< 0.001^*^	0.038*	< 0.001^*^

TRAIN, train-test;

* Significant *p*-values after multiple comparison correction.

**Table 5:** Database-wise *p*-values.

	**TSA>RPA**	**TRAIN>TSA**	**TRAIN>RPA**
2003–2015	< 0.001	1.000	0.663
2008–2014	0.002^*^	0.877	0.326
2009–2014	< 0.001^*^	1.000	0.561
Brain invaders 2013a	< 0.001^*^	1.000	1.000
EPFL P300 dataset	< 0.001^*^	1.000	0.102
2001–2014	0.004^*^	0.468	0.191
2001–2015	< 0.001^*^	0.133	0.005^*^
2002–2014	< 0.001^*^	0.139	< 0.001^*^
2004–2014	0.515	0.641	0.552
Alexandre motor imagery	0.636	0.353	0.280
Cho 2017	< 0.001^*^	0.098	< 0.001^*^
Grosse-Wentrup 2009	0.097	0.083	0.071
Physionet motor imagery	< 0.001^*^	< 0.001^*^	< 0.001^*^
Weibo 2014	1.000	0.099	0.189
Zhou 2016	0.071	0.453	0.398
SSVEP exoskeleton	0.002^*^	0.702	0.477
SSVEP Nakanishi	1.000	0.464	0.886
SSVEP Wang	0.989	< 0.001^*^	< 0.001^*^

TRAIN, train-test;

* Significant *p*-values after multiple comparison correction.

**Table 6:** *p*-values for each paradigm and in global for all tests that have been done. The global *p*-values are the combination of the *p*-values for all databases regardless of their paradigm.

	**TSA>RPA**	**TRAIN>TSA**	**TRAIN>RPA**
p300	< 0.001^*^	1.000	0.961
Imagery	< 0.001^*^	< 0.001^*^	< 0.001^*^
SSVEP	< 0.654	< 0.001^*^	< 0.001^*^
Global	< 0.001^*^	1.000	< 0.001^*^

TRAIN, train-test; ^*^Significant *p*-values after multiple comparison correction.

So far we have focused on cross-subject transfer learning, however the method we propose can also be used to transfer different information, such as from one session to another with the same subject or from one task to the another. In order to ensure the ability of our method to reduce the inter-session variability, we used the dataset having multiple sessions and processed results for inter-session cross-validation. In order to do so, we used the data of one session as a source, then for each other sessions with 80% test and 20% training data split we trained the transfer learning model and tested the results. The processed is then repeated using each session as the source. We compared four different methods:

- Tangent Space Alignment (TSA), our method,
- Riemannian Procrustes Analysis (RPA) used as a comparison in this article,
- Usual train-test method using only target data,
- Direct testing (DCT) using algorithms trained on the source without aligning target data by a rotation (recentering only).

**Figure 5:**
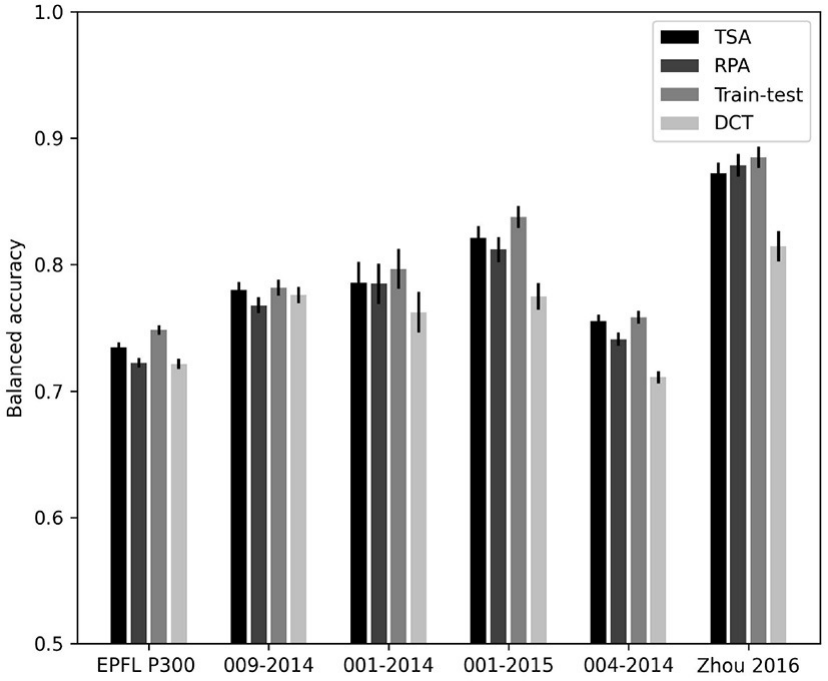
Bar graph giving the inter-session balanced accuracy for the databases possessing multiple sessions.

The results are given in Figure 5 and presented numerically in Table 7.

**Table 7:** Inter-session balanced accuracy table for each dataset and methods.

	**Train-test**	**TSA**	**RPA**	**DCT**
EPFL P300	74.85 ± 0.38	73.46 ± 0.38	72.24 ± 0.39	72.16 ± 0.40
2009–2014	78.19 ± 0.64	78.00 ± 0.65	76.79 ± 0.63	77.59 ± 0.64
2001–2014	79.66 ± 1.57	78.56 ± 1.66	78.48 ± 1.61	76.23 ± 1.63
2001–2015	83.78 ± 0.86	82.10 ± 0.95	81.19 ± 0.99	77.49 ± 1.03
2004–2014	75.83 ± 0.52	75.55 ± 0.50	74.11 ± 0.53	71.09 ± 0.50
Zhou 2016	88.48 ± 0.86	87.22 ± 0.84	87.83 ± 0.90	81.45 ± 1.19

These results are coherent with the previous ones. They yield accuracies slightly improved as compared to sessions mixed all together, which is expected. Moreover, the proposed method performs better than RPA on all databases with the exception of Zhou 2016.

## 4. Discussion

The extensive analysis we have carried out shows that for the ERP paradigm TSA clearly outperforms RPA. For the MI paradigm we observe that TSA performs better than RPA and that the classical train-test cross-validation outperforms both TSA and RPA. For SSVEP, the classical train-test cross-validation outperforms both TSA and RPA. In global, RPA is outperformed by both TSA and the classical train-test cross-validation. Based on Table 6, we do not conclude on the superiority of TSA as compared to train-test in terms of accuracy as the global value that can be found is mainly due to very large values found for the ERP paradigm. To conclude with the results, it has been shown that TSA outperforms RPA for the large majority of the databases we have used in this analysis, reaching an accuracy pretty close to the optimal train-test method. It is also a clear improvement from a methodological point of view in comparison to RPA as it naturally allows transfer learning between datasets with different number of channels, where RPA needs some extensions in order to do so (Rodrigues et al., 2021).

In this paper, we have introduced a new method for transfer learning inter and intra subject for brain computer interfaces. Our study indicates that it outperforms a state-of-the-art analogous method (RPA). However, it still does not reach the same accuracy that can be achieved with a classical test-train cross-validation procedure for the motor imagery and SSVEP paradigm. Further research is needed to understand why the performance of the TSA method is clearly superior for the ERP paradigm.

In terms of computation time, since we have a closed form for the rotation of TSA method, it is way faster than the RPA method, where an optimization on the Grassmann manifold is performed. However, even if our method can be faster by one order of magnitude, with *N*_*c*_ being the size of the pre-covariance signal (number of channels plus number of channels of the average target for ERP, number of channels for MI, number of classes times number of channels for SSVEP) we compute rotations with size [$\frac{N_{c}*\left(N_{c}+1\right)}{2},\frac{N_{c}*\left(N_{c}+1\right)}{2}$] where RPA computes rotation with size (*N*_*c*_, *N*_*c*_). This means that for datasets with a significant amount of channels the computational advantage of TSA will tend to vanish. Table 8 shows the average time of computation for each dataset if no spatial filter were to be applied before the covariance estimation, sometimes yielding big covariance matrices. Usually spatial filter are applied so the low time values are those that are to be usually encountered. Moreover, when computing the rotations for TSA, we can consider only a number of channels that will result in a proportion of the global variance of the data. This means that we do not need to compute the whole [$\frac{N_{c}*\left(N_{c}+1\right)}{2},\frac{N_{c}*\left(N_{c}+1\right)}{2}$] rotation matrix, we can select only a few singular values and their corresponding vectors, highly reducing computation time if needed.

**Table 8:** Database-wise average computation time for both TSA and RPA methods.

	**TSA (s)**	**RPA (s)**	**Ratio**	**Cov shape**
2003–2015	1.027	11.889	11.57	16*16
2008–2014	1.711	10.874	6.36	16*16
2009–2014	0.897	6.118	6.82	32*32
Brain invaders 2013a	0.774	13.368	17.26	32*32
EPFL P300 dataset	1.341	8.607	6.42	64*64
2001–2014	0.999	5.075	5.08	22*22
2001–2015	0.664	4.362	6.57	13*13
2002–2014	0.416	2.241	5.39	15*15
2004–2014	0.029	0.166	5.74	3*3
Alexandre motor imagery	0.067	0.777	11.61	16*16
Cho 2017	5.299	16.858	3.18	64*64
Grosse-Wentrup 2009	161.942	113.926	0.70	128*128
Physionet motor imagery	2.739	4.432	1.62	64*64
Weibo 2014	3.719	13.282	3.57	60*60
Zhou 2016	0.468	3.752	8.02	14*14
SSVEP exoskeleton	0.056	1.184	21.22	16*16
SSVEP Nakanishi	0.41	0.970	23.81	16*16
SSVEP Wang	125.949	22.320	0.18	124*124

We also observed that in some cases, mainly for ERPs, skipping the rescaling of the target data will lead to improved classification results. Further research is needed to fully understand the role of rescaling in Procrustes-like transfer learning methods.

In this article, we have proposed a procedure to improve the rank deficiency of the cross-product matrix, making the result more stable and more accurate. However, this improvement also presents some downsides. When using only the average point of the data for both source and target data, the method will be pretty sensitive to noise since the number of points used for means computation is drastically reduced. Adding trimmed means and/or medians could make the method less sensitive to noise. Additionally, when augmenting **C_st_** using PCA, the more groups will be created, the more each group will be sensitive to noise. By reducing the number of points in a group one increases the impact of artifacts on the average point. Furthermore, PCA is an algorithm that is pretty sensitive to noise and could be replaced by one of its robust variant. It is to be noticed that a low number of groups in general allows a good approximation of the shape of the data. Of course, artifact correction or removal would allow better performances.

It should be reminded that in this article we did not apply any form of pre-processing in order to increase the overall accuracy. We have done so because the goal of the article is to compare transfer learning methods on a large amount of data and in a wide variety of real-world situations, that is, on noisy data. For the same reason, we are using all the data of a subject without making any adaptation from one session to the other. This choice obviously leads to reduced overall accuracy, resulting in an important decrease in the number of subjects used for the final average results. It is important to do so, however, since, as it has been found in previous studies, there is a large gap between “good” and “bad” subjects in transfer learning accuracy (Zanini et al., 2018; Rodrigues et al., 2019).

Another point to mention is that even though our method has been tested extensively on many databases, there are even more databases to test on. Additionally, some paradigms such as affective BCI have not been investigated in this article. Investigation on cross-database transfer learning is still to be done.

As all efficient transfer learning methods, TSA can be very helpful when used along with a machine learning model that takes too much time for training on new data for online sessions. TSA also allows the alignment of multiple subjects' data into the same feature space. Such alignment could improve classification accuracy of multiple subjects and allow the training of robust classifier on aligned data that will give improved results for new subjects once they are aligned. This is the object of current investigation in our laboratory.

## Data availability statement

Publicly available datasets were analyzed in this study. This data can be found at: http://moabb.neurotechx.com/docs/datasets.html.

## Author contributions

AB performed the research, analyzed the data, and wrote the manuscript. MC suggested the original idea for the method. MC and JM designed, reviewed, and edited the manuscript. All authors have read and approved the submitted manuscript.

## Funding

This research has been partially supported by CNRS grant 80∣PRIMES TrAp and by ANR grant Hifi (ANR-20-CE17-0023).

## Conflict of interest

The authors declare that the research was conducted in the absence of any commercial or financial relationships that could be construed as a potential conflict of interest.

## Publisher's note

All claims expressed in this article are solely those of the authors and do not necessarily represent those of their affiliated organizations, or those of the publisher, the editors and the reviewers. Any product that may be evaluated in this article, or claim that may be made by its manufacturer, is not guaranteed or endorsed by the publisher.
