# Supplementary figures and images for: Tangent space alignment: Transfer learning for Brain-Computer Interface
Source: Front Hum Neurosci. 2022 Dec 2;16:1049985. doi: 10.3389/fnhum.2022.1049985 (PMC9755175; doi:10.3389/fnhum.2022.1049985)

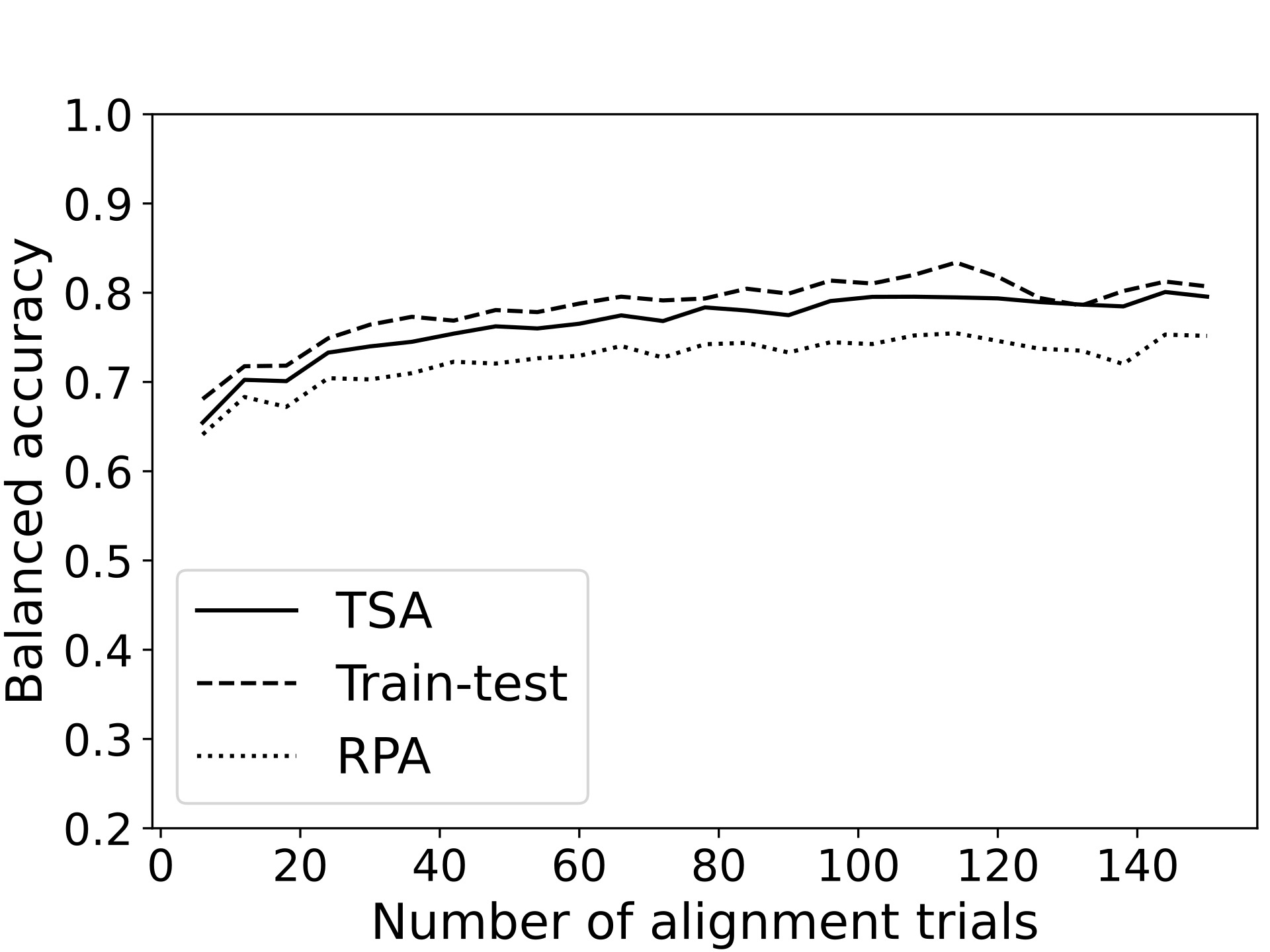

Supplement: Supplementary file 1 [file Data_Sheet_1.ZIP › MI/accuracy_002-2014_pca3threshold0.60.jpg]

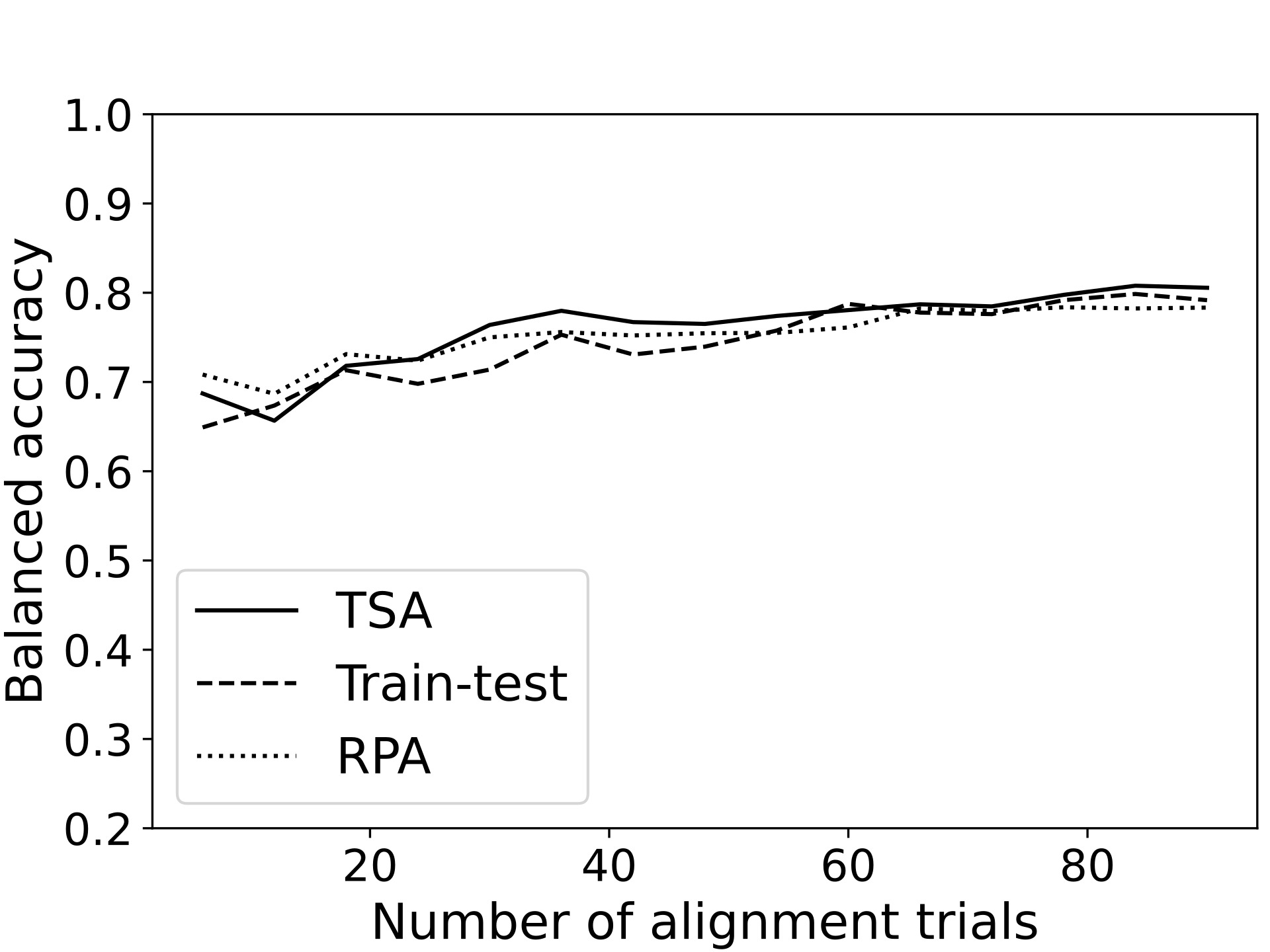

Supplement: Supplementary file 1 [file Data_Sheet_1.ZIP › MI/accuracy_004-2014_pca3threshold0.60.jpg]

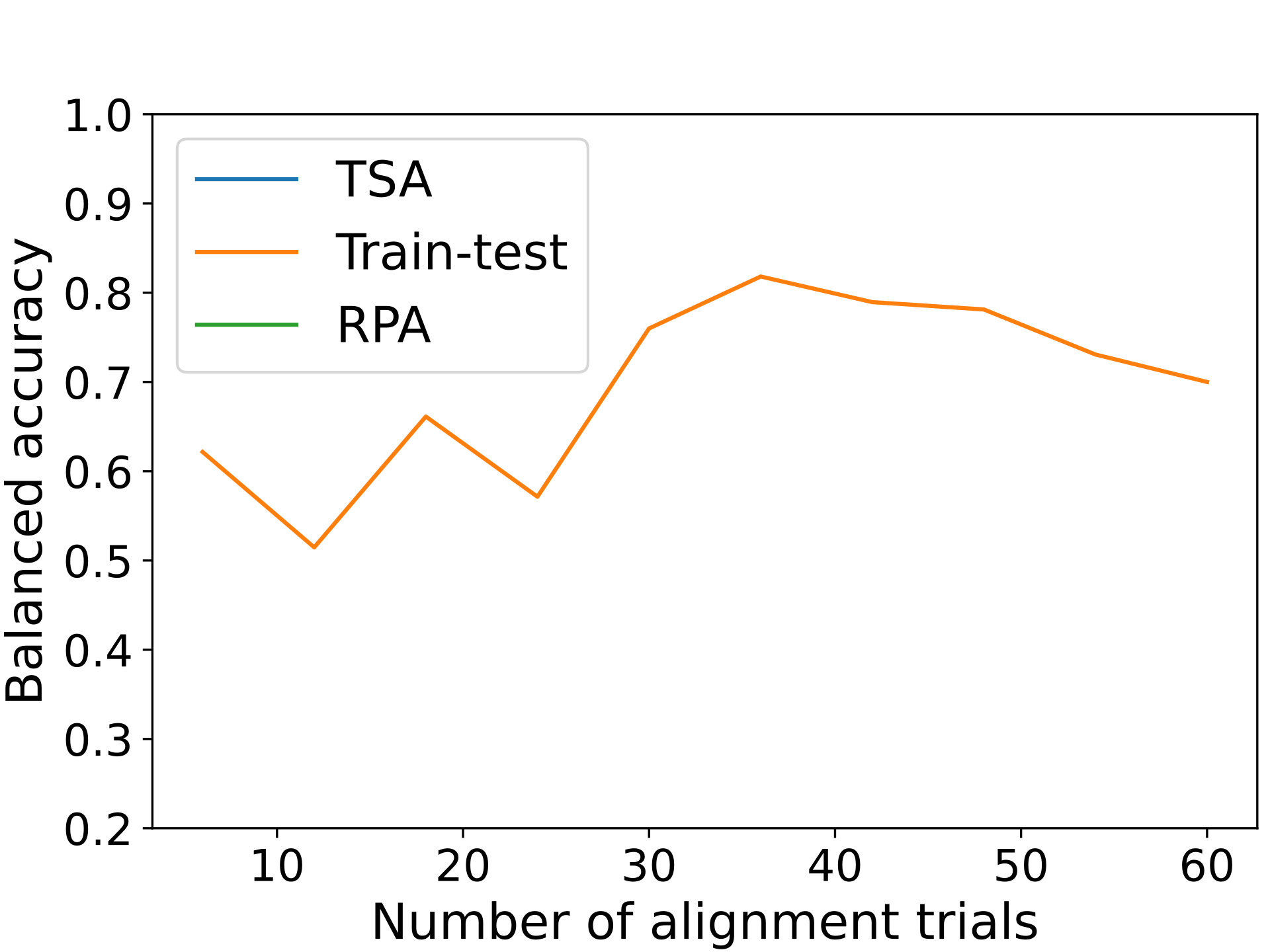

Supplement: Supplementary file 1 [file Data_Sheet_1.ZIP › MI/accuracy_004-2015_pca3threshold0.60.jpg]

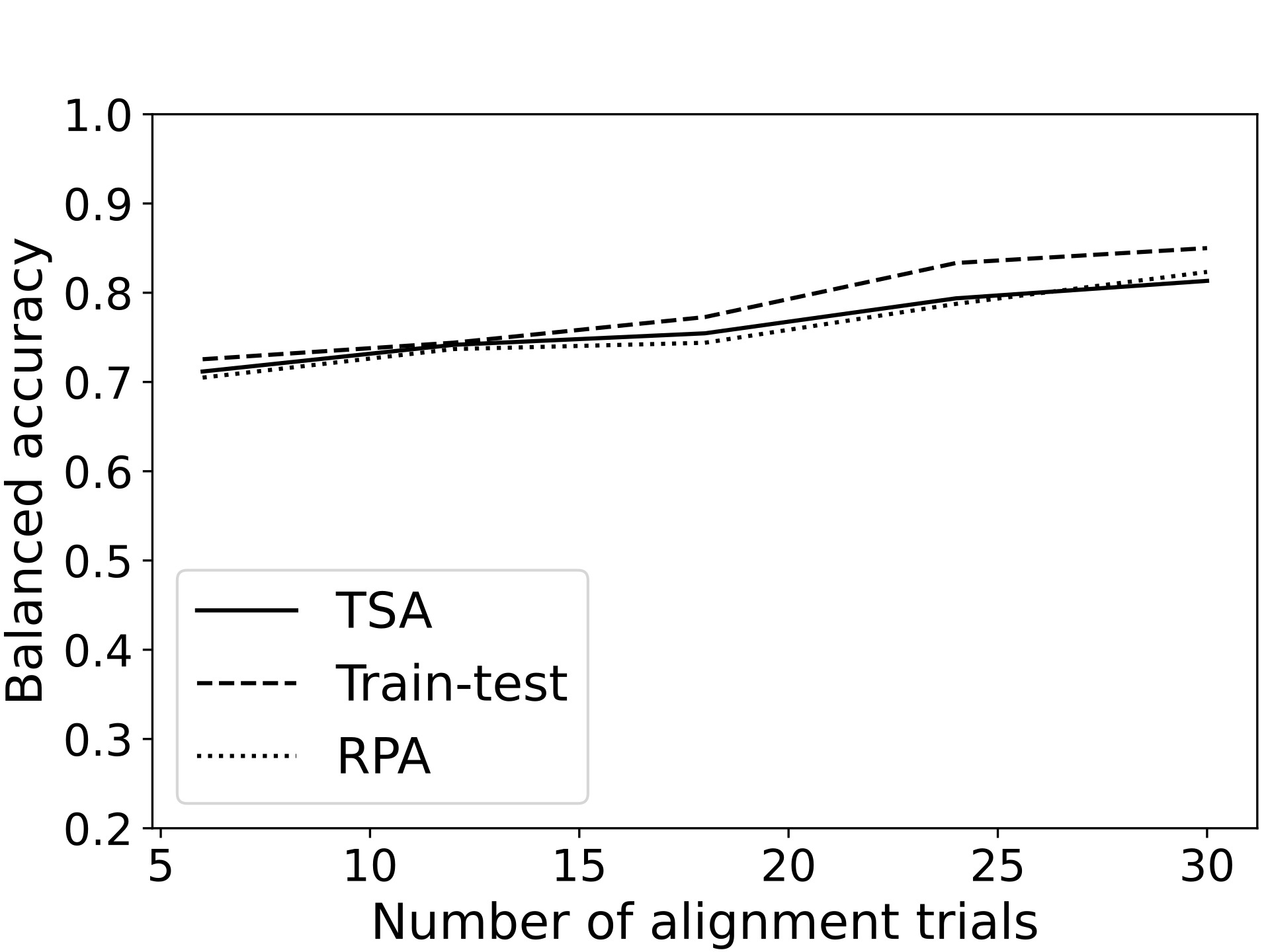

Supplement: Supplementary file 1 [file Data_Sheet_1.ZIP › MI/accuracy_Alexandre_Motor_Imagery_pca3threshold0.60.jpg]

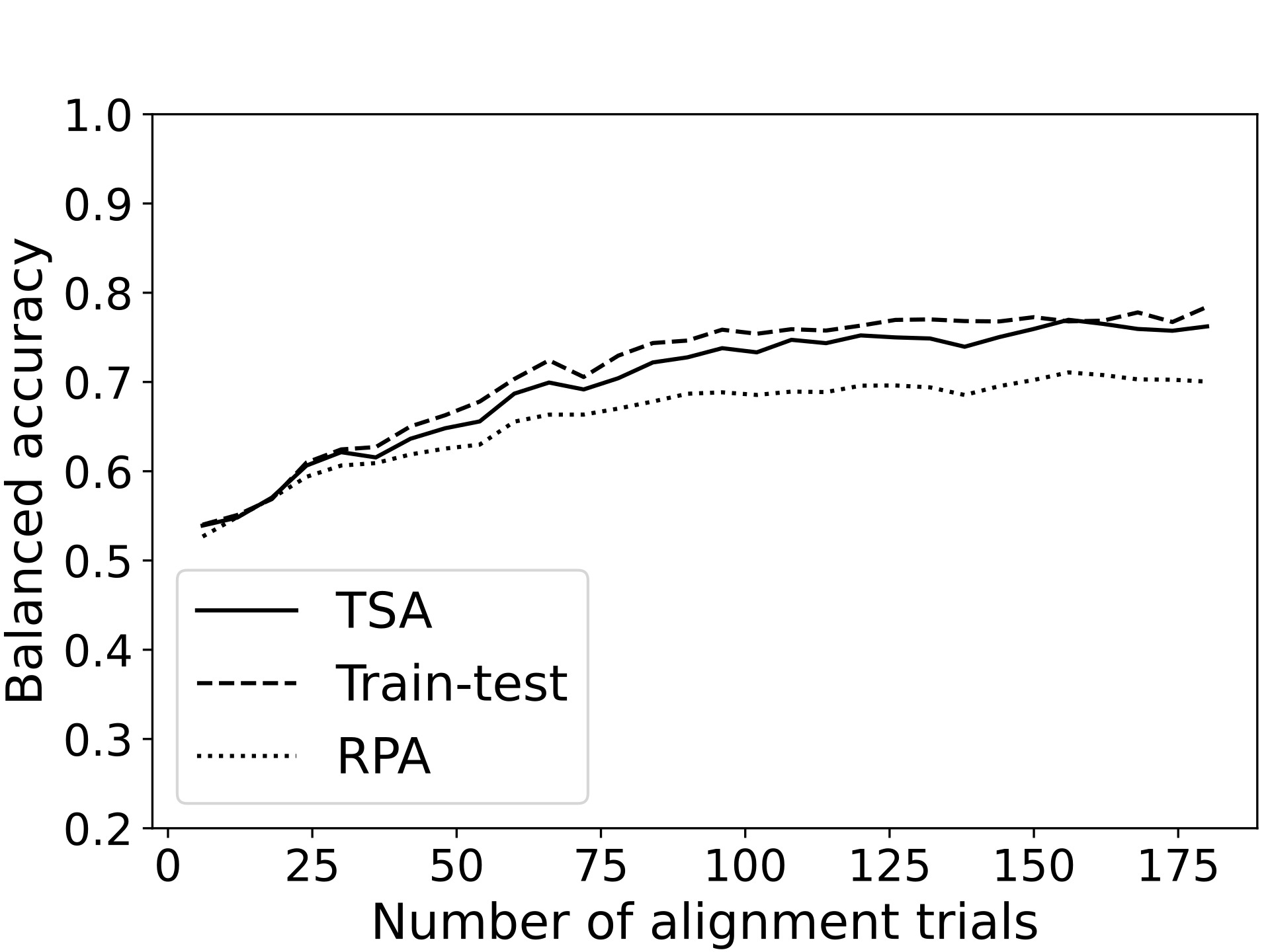

Supplement: Supplementary file 1 [file Data_Sheet_1.ZIP › MI/accuracy_Cho2017_pca3threshold0.60.jpg]

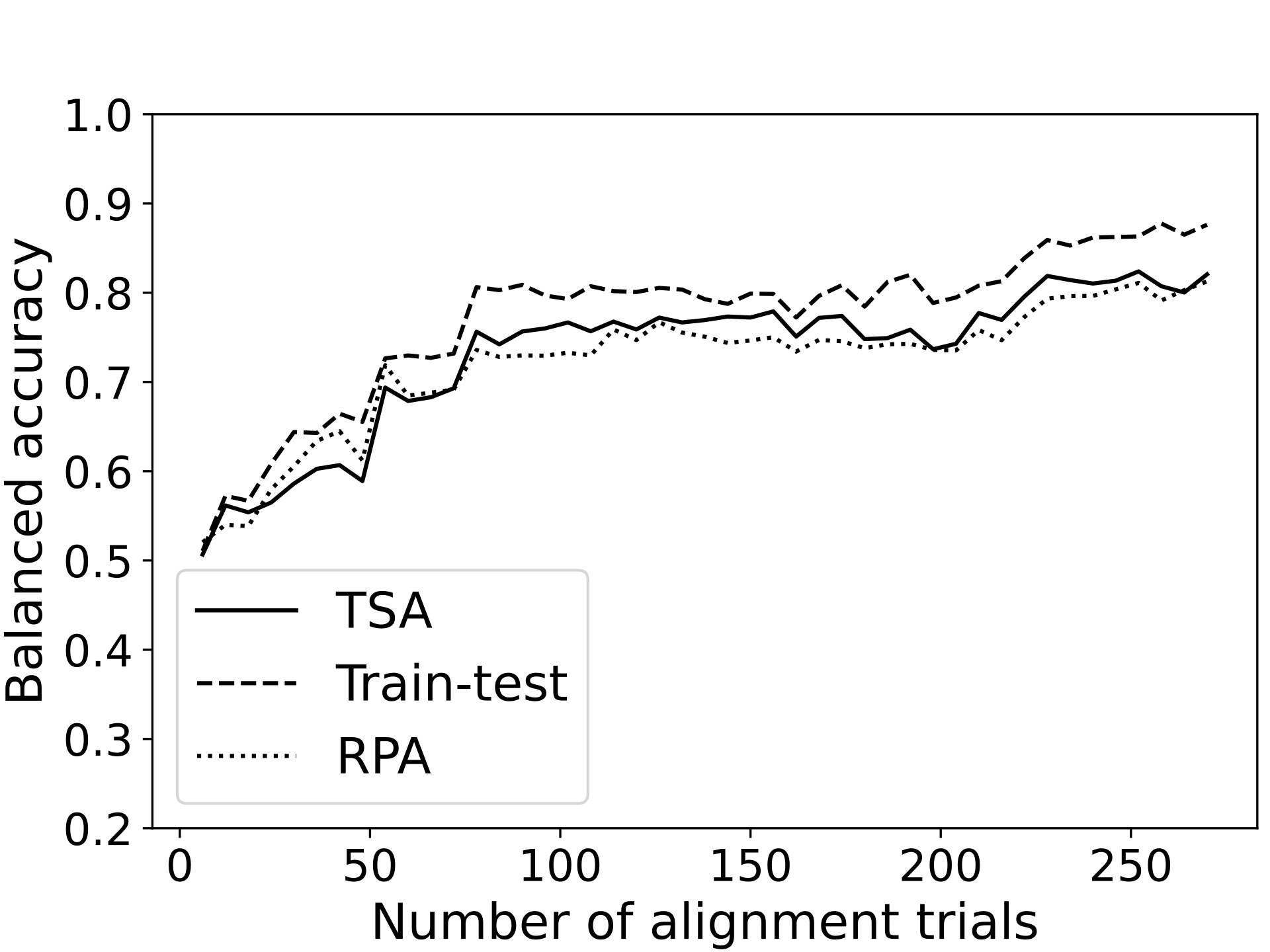

Supplement: Supplementary file 1 [file Data_Sheet_1.ZIP › MI/accuracy_Grosse-Wentrup_2009_pca3threshold0.60.jpg]

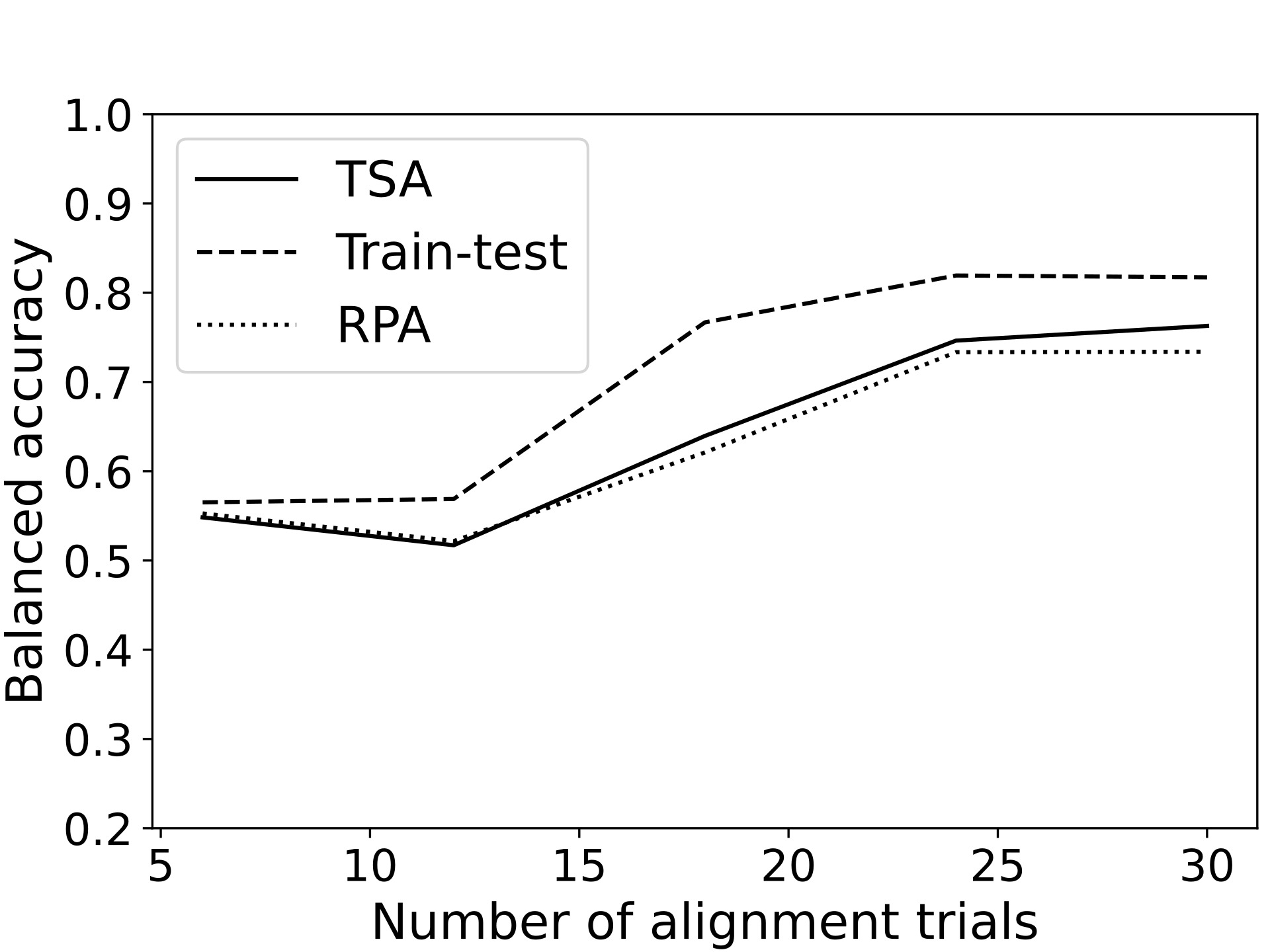

Supplement: Supplementary file 1 [file Data_Sheet_1.ZIP › MI/accuracy_Physionet_Motor_Imagery_pca3threshold0.60.jpg]

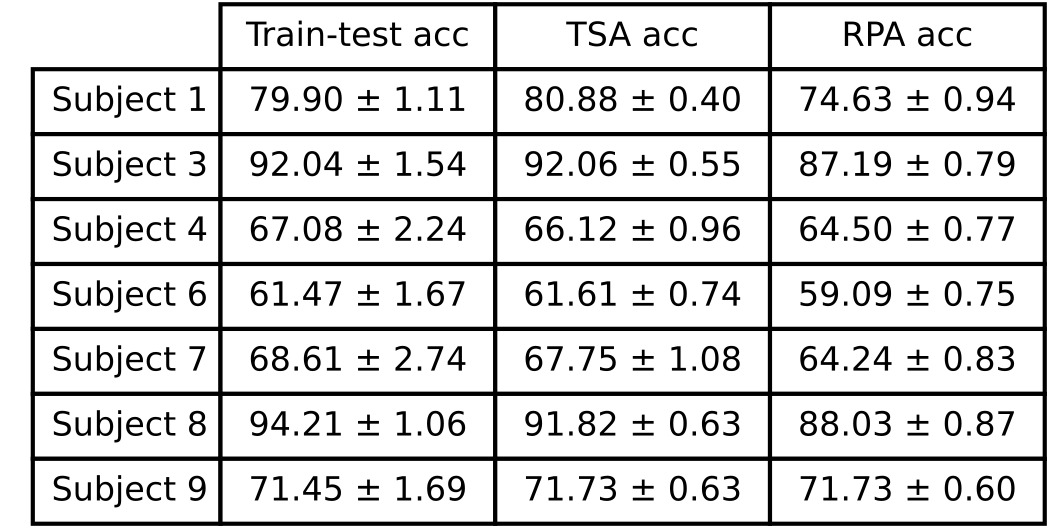

Supplement: Supplementary file 1 [file Data_Sheet_1.ZIP › MI/accuracy_table_001-2014_pca3threshold0.60.jpg]

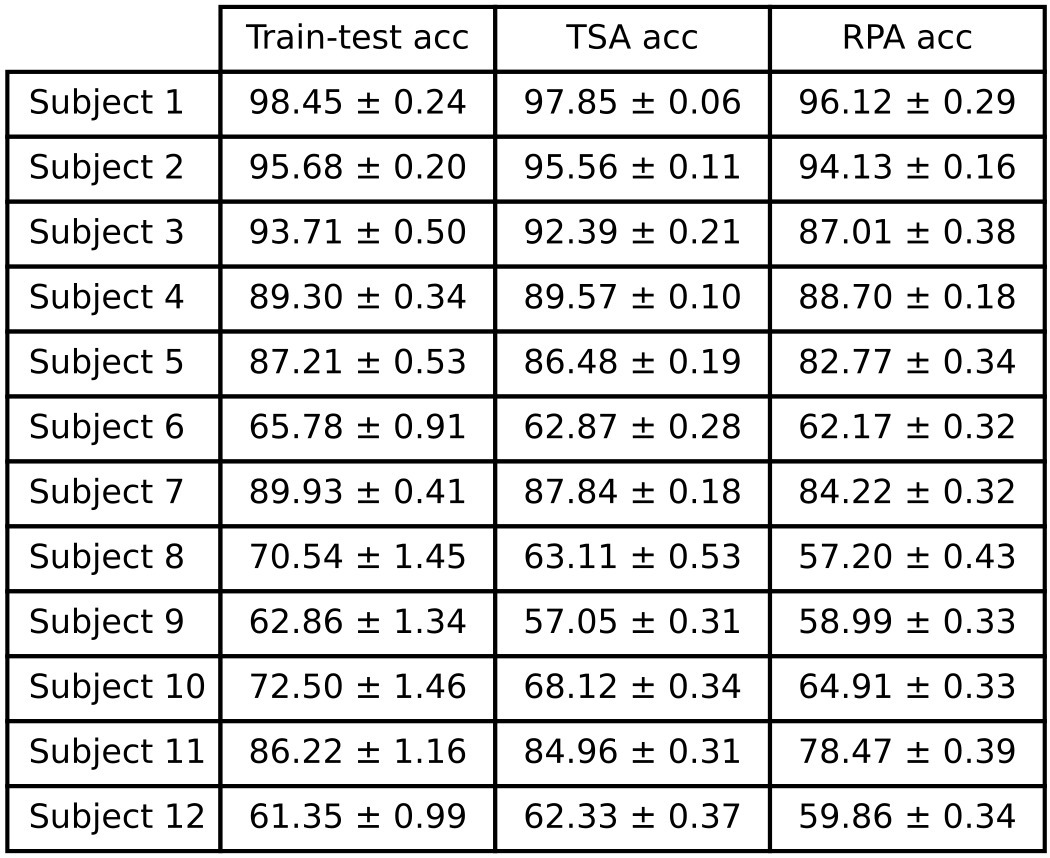

Supplement: Supplementary file 1 [file Data_Sheet_1.ZIP › MI/accuracy_table_001-2015_pca3threshold0.60.jpg]

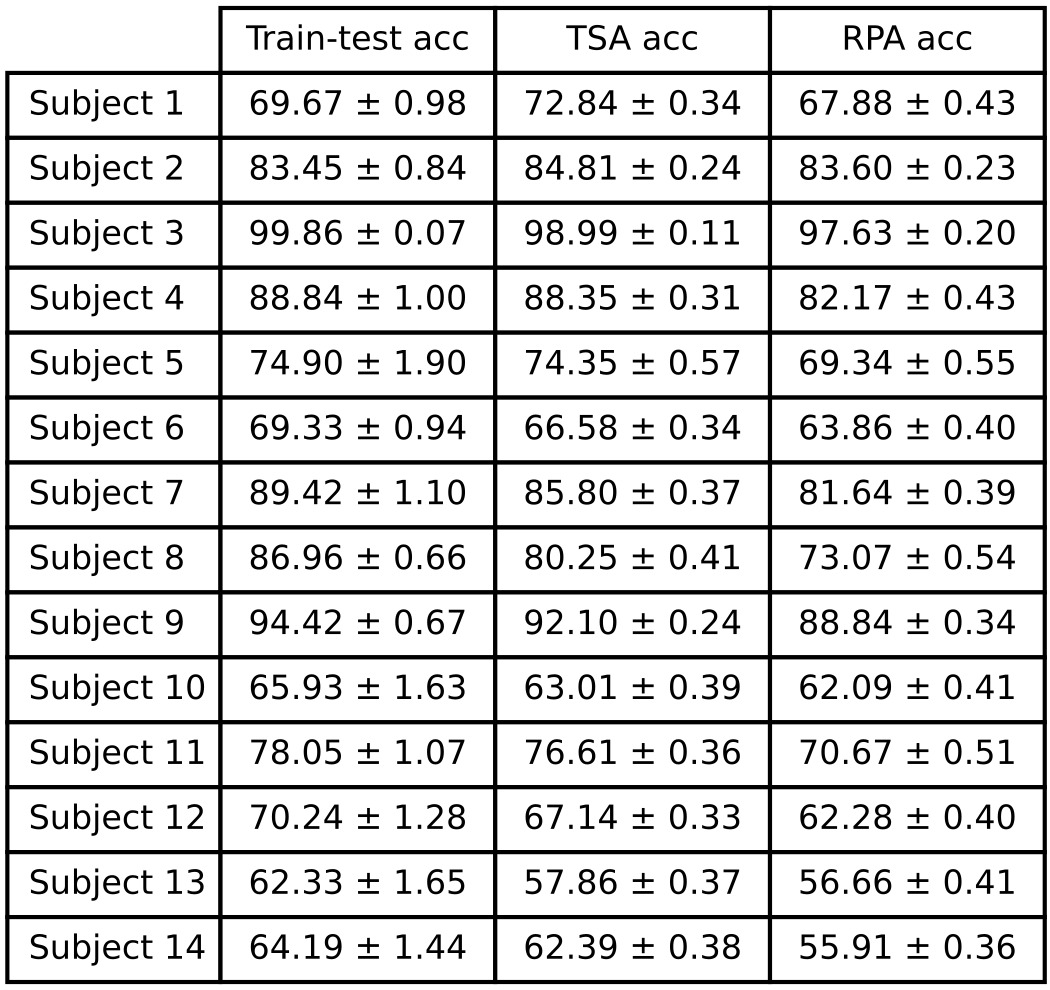

Supplement: Supplementary file 1 [file Data_Sheet_1.ZIP › MI/accuracy_table_002-2014_pca3threshold0.60.jpg]

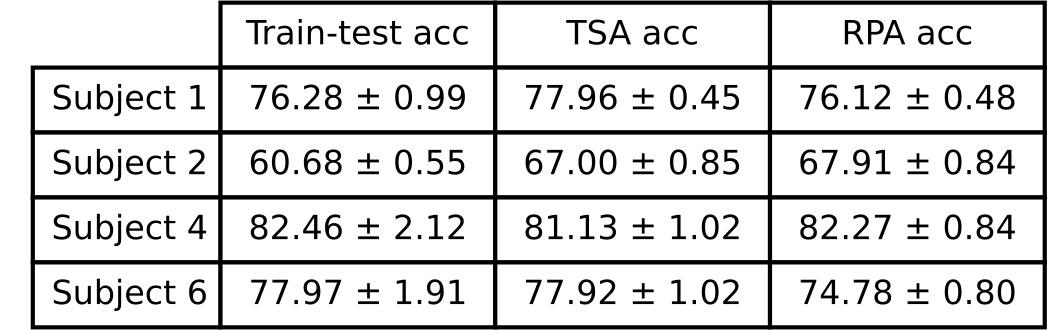

Supplement: Supplementary file 1 [file Data_Sheet_1.ZIP › MI/accuracy_table_004-2014_pca3threshold0.60.jpg]

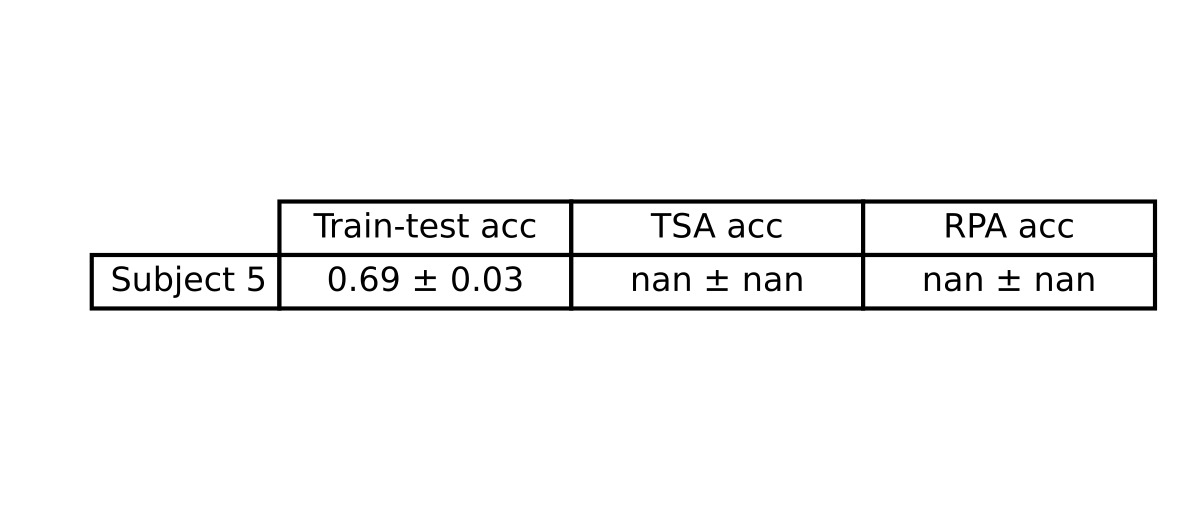

Supplement: Supplementary file 1 [file Data_Sheet_1.ZIP › MI/accuracy_table_004-2015_pca3threshold0.60.jpg]

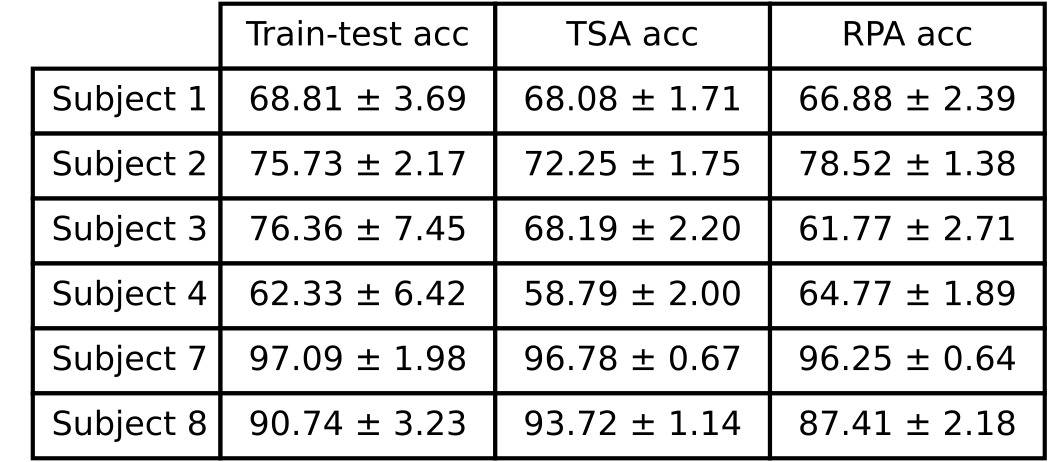

Supplement: Supplementary file 1 [file Data_Sheet_1.ZIP › MI/accuracy_table_Alexandre_Motor_Imagery_pca3threshold0.60.jpg]

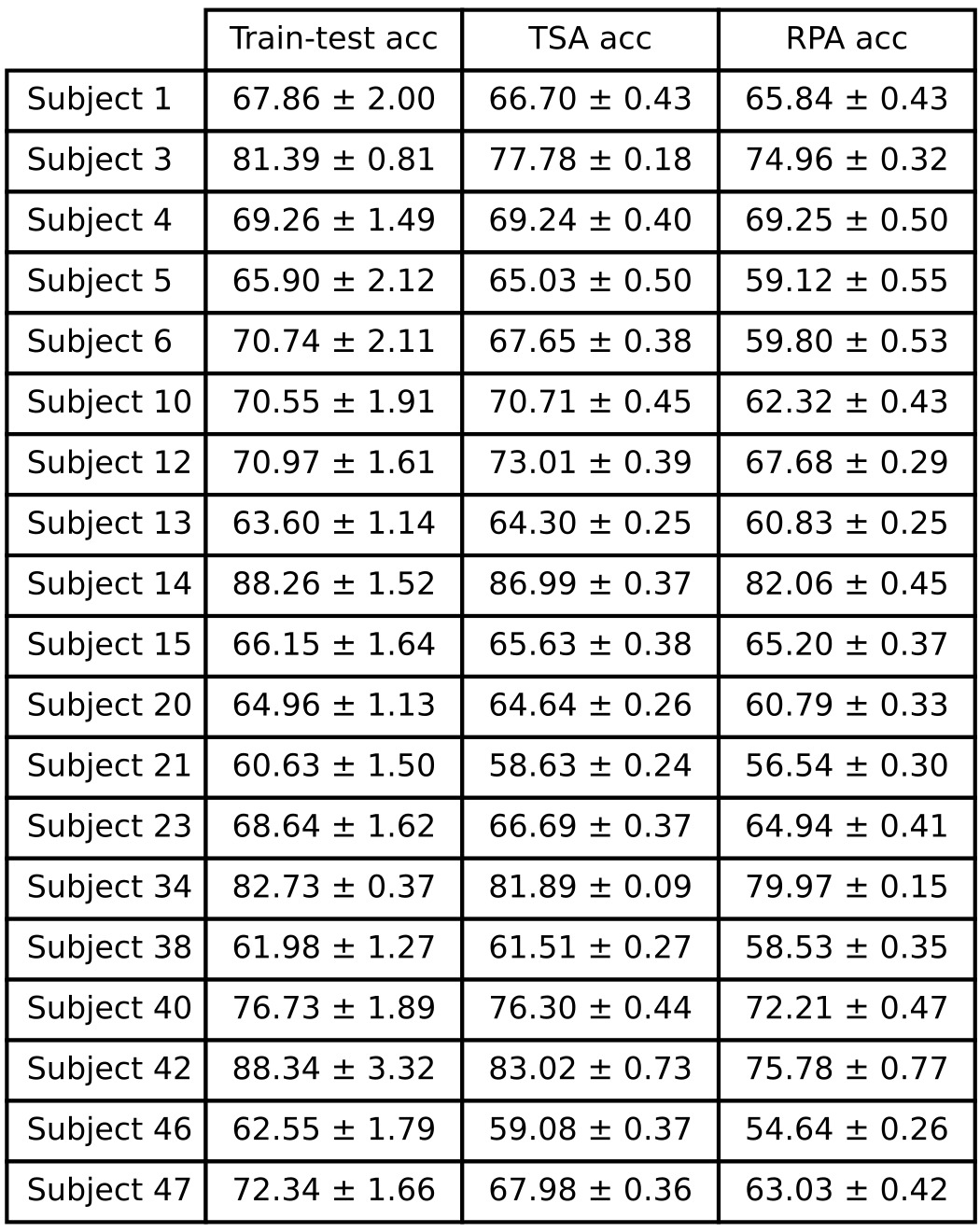

Supplement: Supplementary file 1 [file Data_Sheet_1.ZIP › MI/accuracy_table_Cho2017_pca3threshold0.60.jpg]

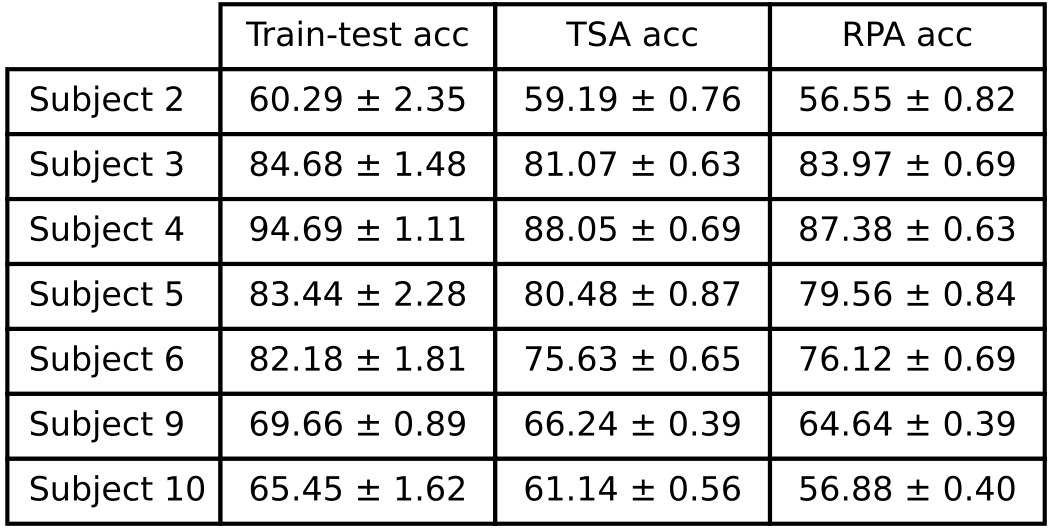

Supplement: Supplementary file 1 [file Data_Sheet_1.ZIP › MI/accuracy_table_Grosse-Wentrup_2009_pca3threshold0.60.jpg]

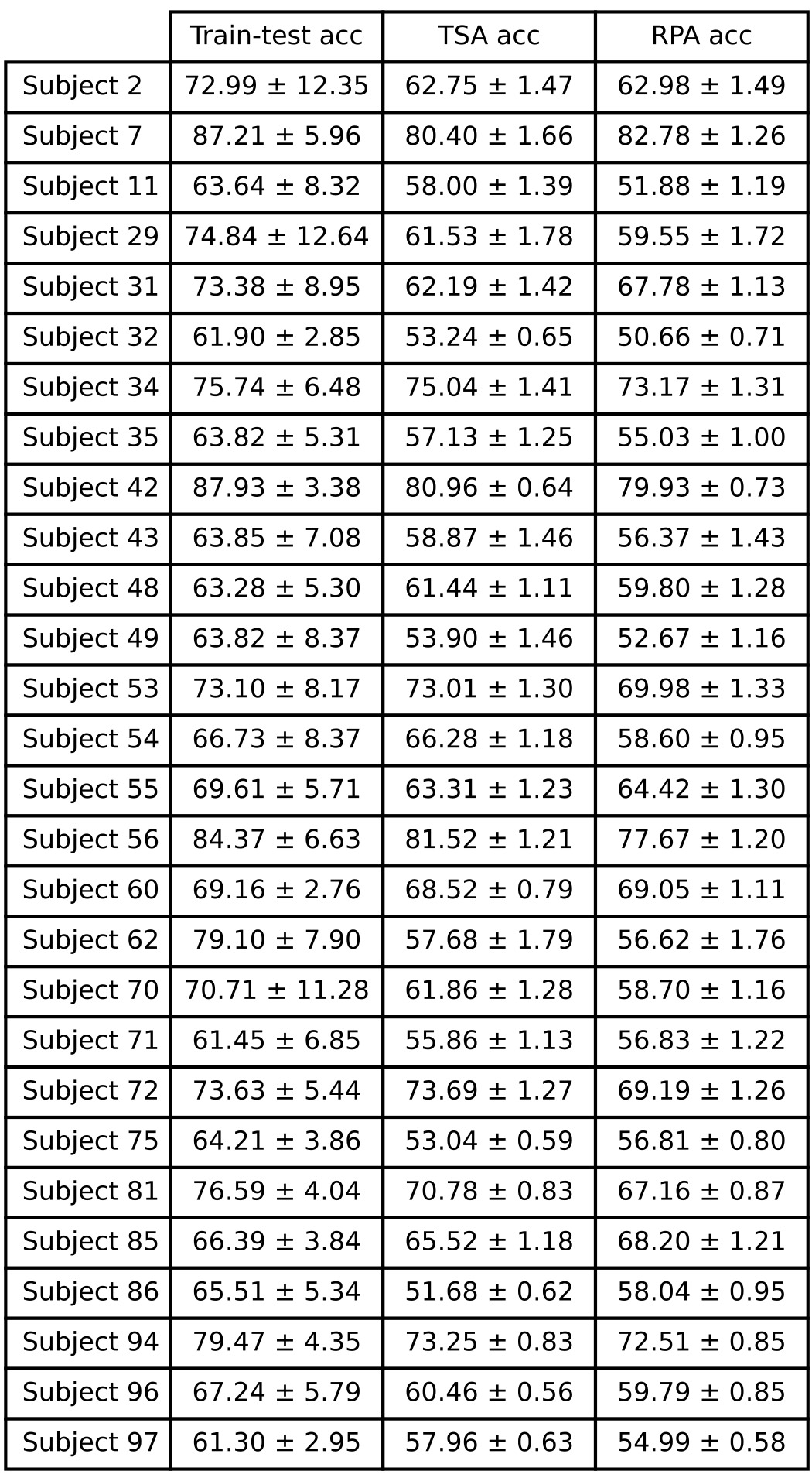

Supplement: Supplementary file 1 [file Data_Sheet_1.ZIP › MI/accuracy_table_Physionet_Motor_Imagery_pca3threshold0.60.jpg]

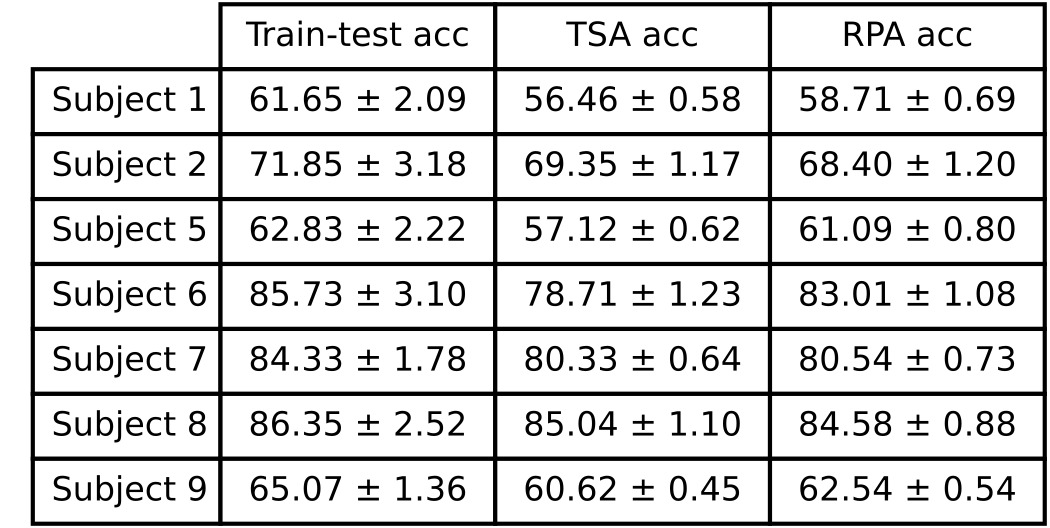

Supplement: Supplementary file 1 [file Data_Sheet_1.ZIP › MI/accuracy_table_Weibo_2014_pca3threshold0.60.jpg]

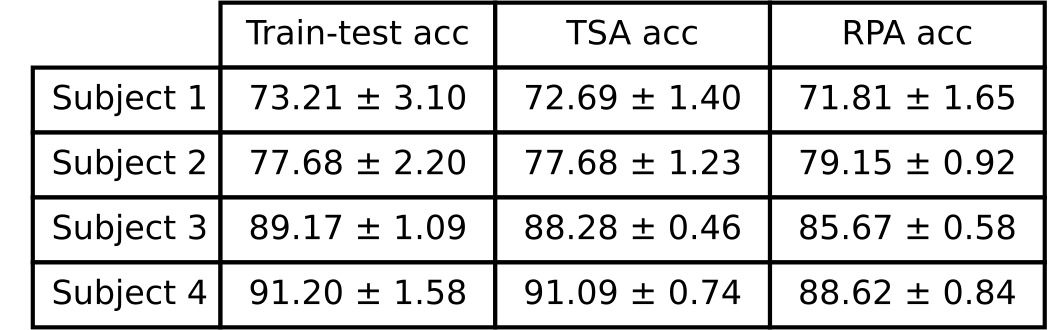

Supplement: Supplementary file 1 [file Data_Sheet_1.ZIP › MI/accuracy_table_Zhou_2016_pca3threshold0.60.jpg]

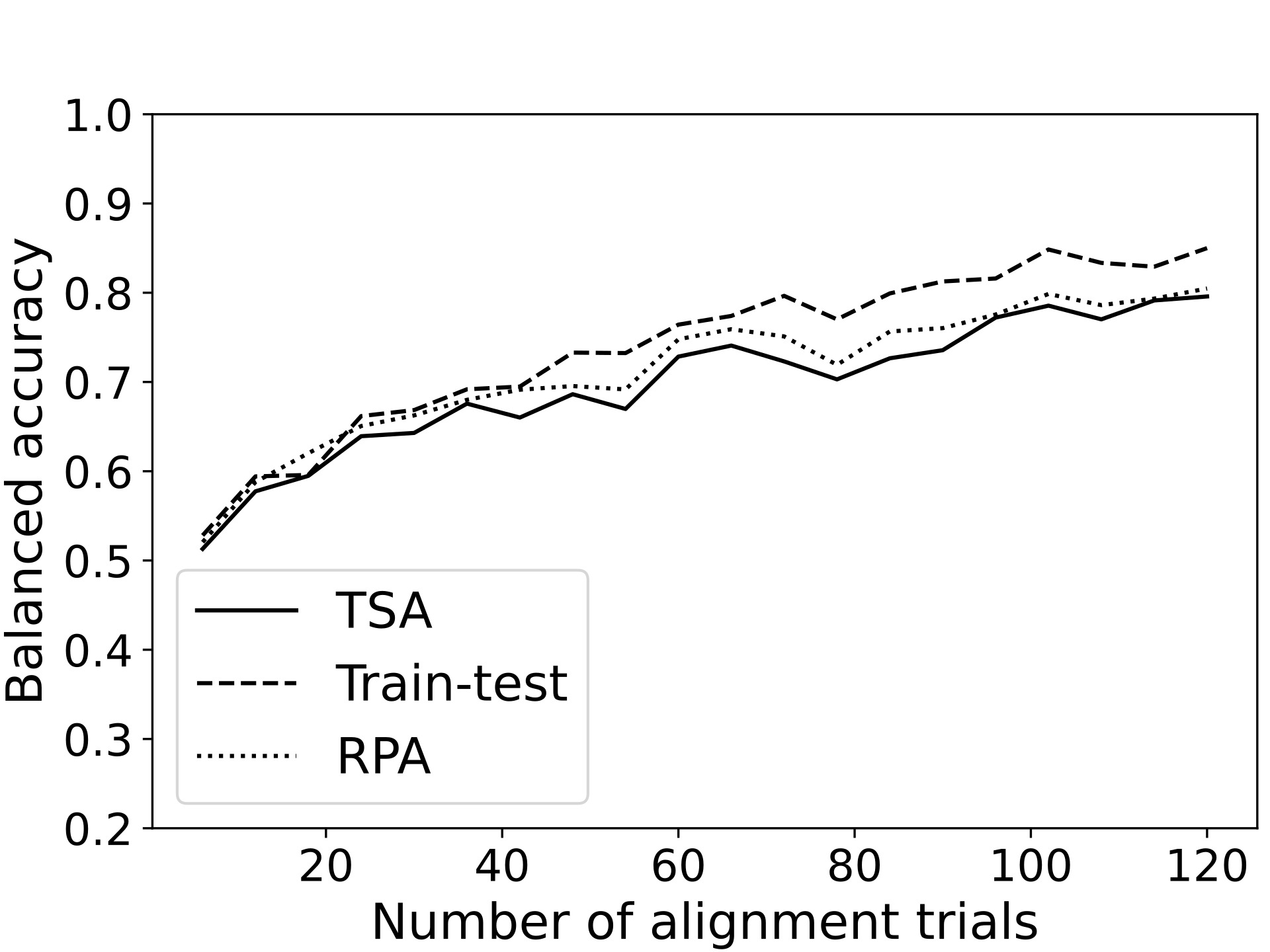

Supplement: Supplementary file 1 [file Data_Sheet_1.ZIP › MI/accuracy_Weibo_2014_pca3threshold0.60.jpg]

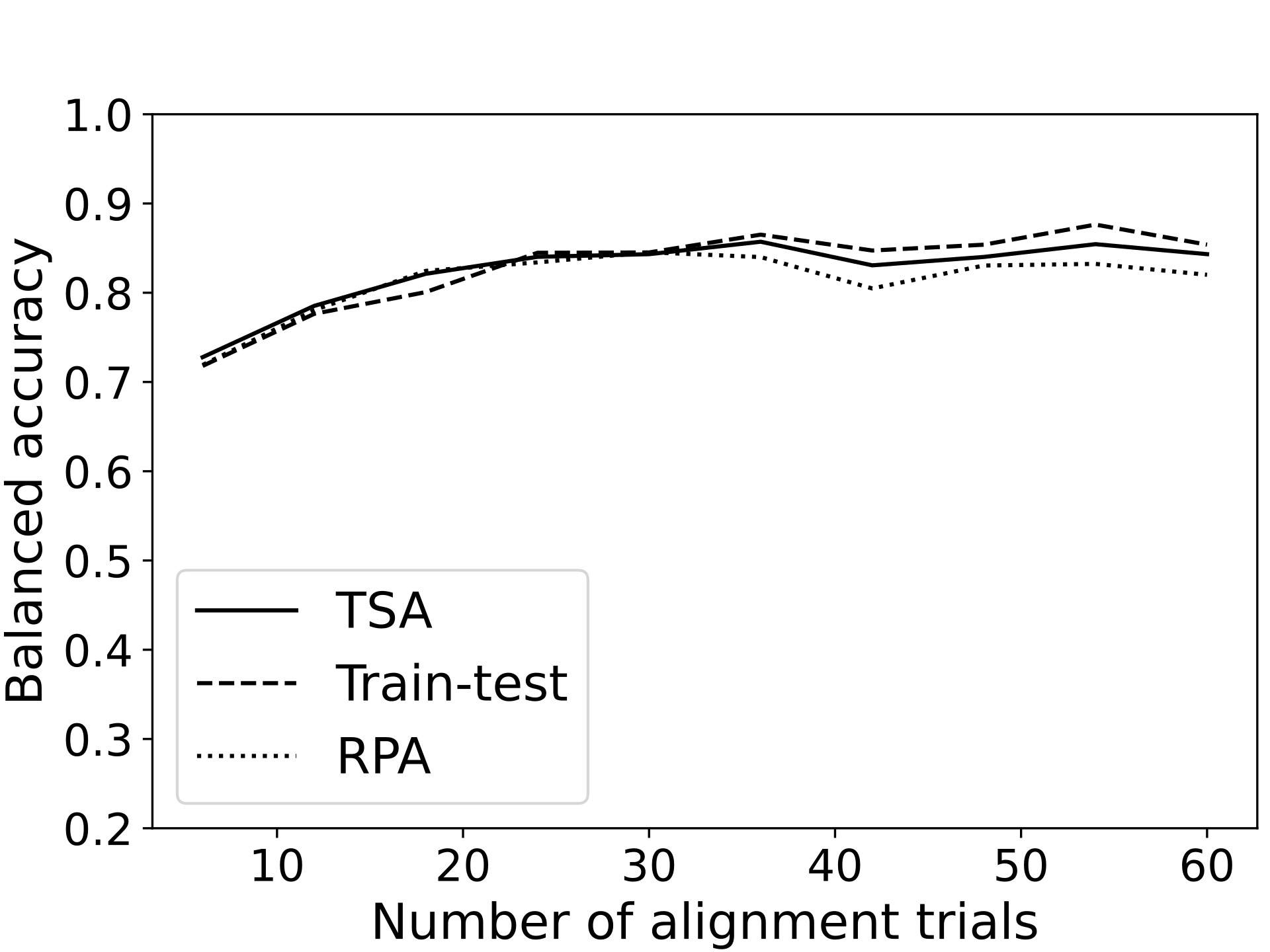

Supplement: Supplementary file 1 [file Data_Sheet_1.ZIP › MI/accuracy_Zhou_2016_pca3threshold0.60.jpg]

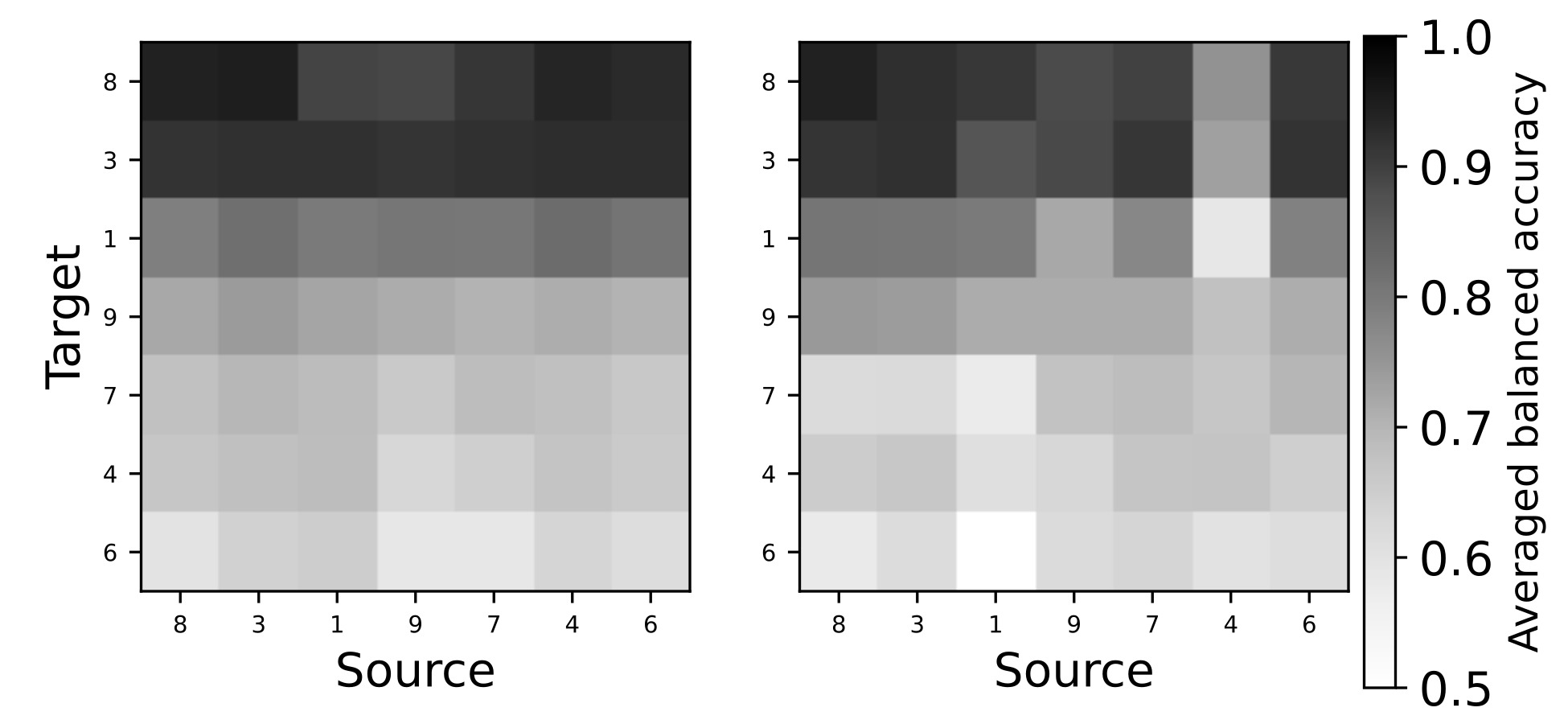

Supplement: Supplementary file 1 [file Data_Sheet_1.ZIP › MI/seriation_both_001-2014_pca3threshold0.60.jpg]

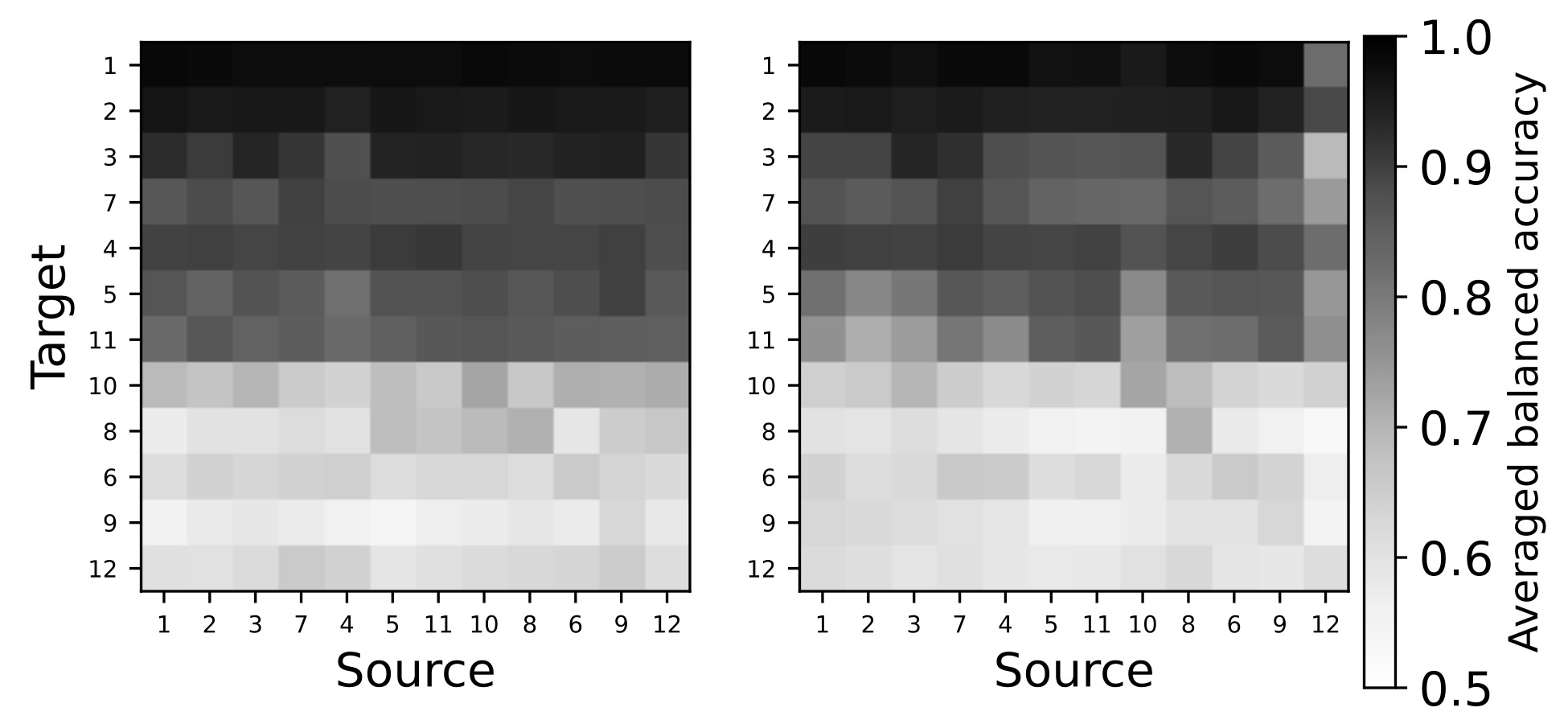

Supplement: Supplementary file 1 [file Data_Sheet_1.ZIP › MI/seriation_both_001-2015_pca3threshold0.60.jpg]

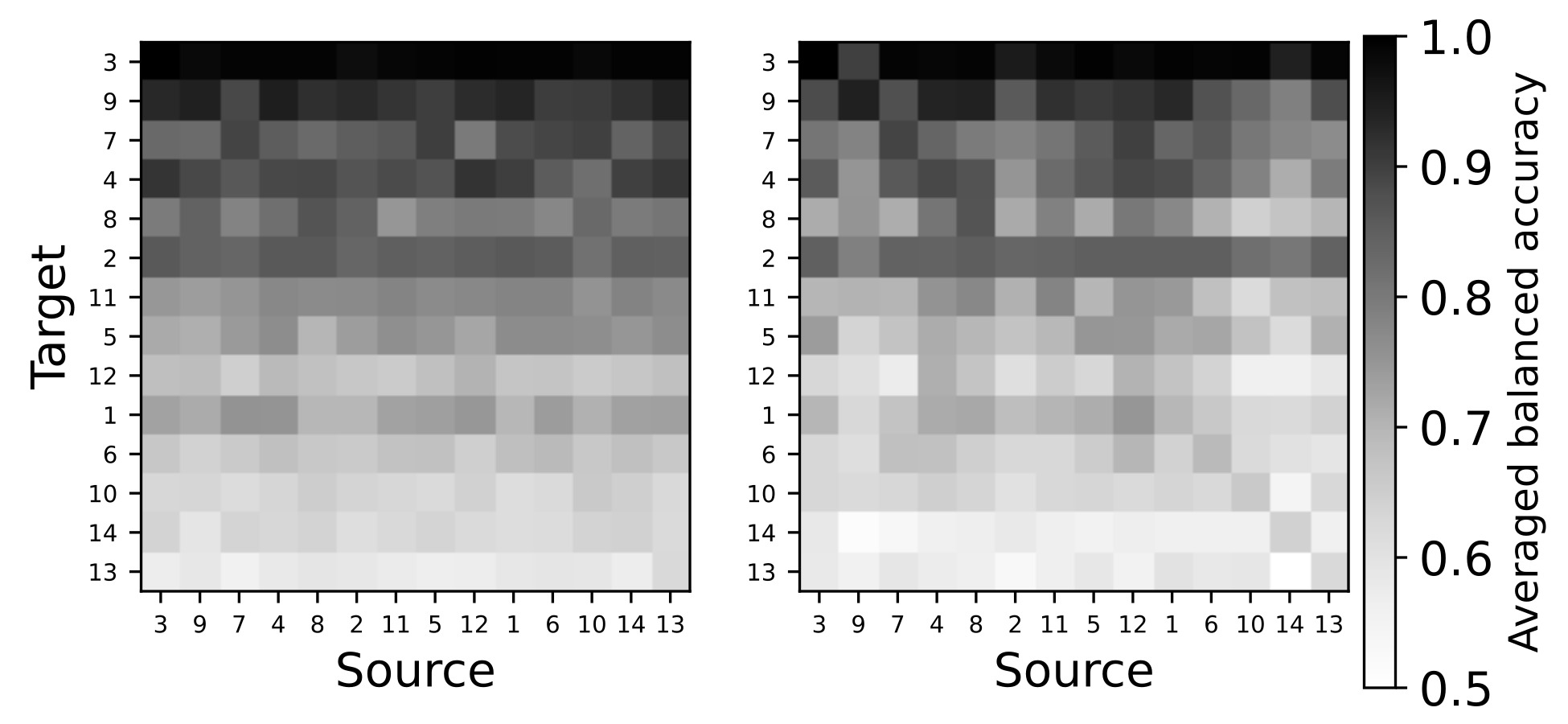

Supplement: Supplementary file 1 [file Data_Sheet_1.ZIP › MI/seriation_both_002-2014_pca3threshold0.60.jpg]

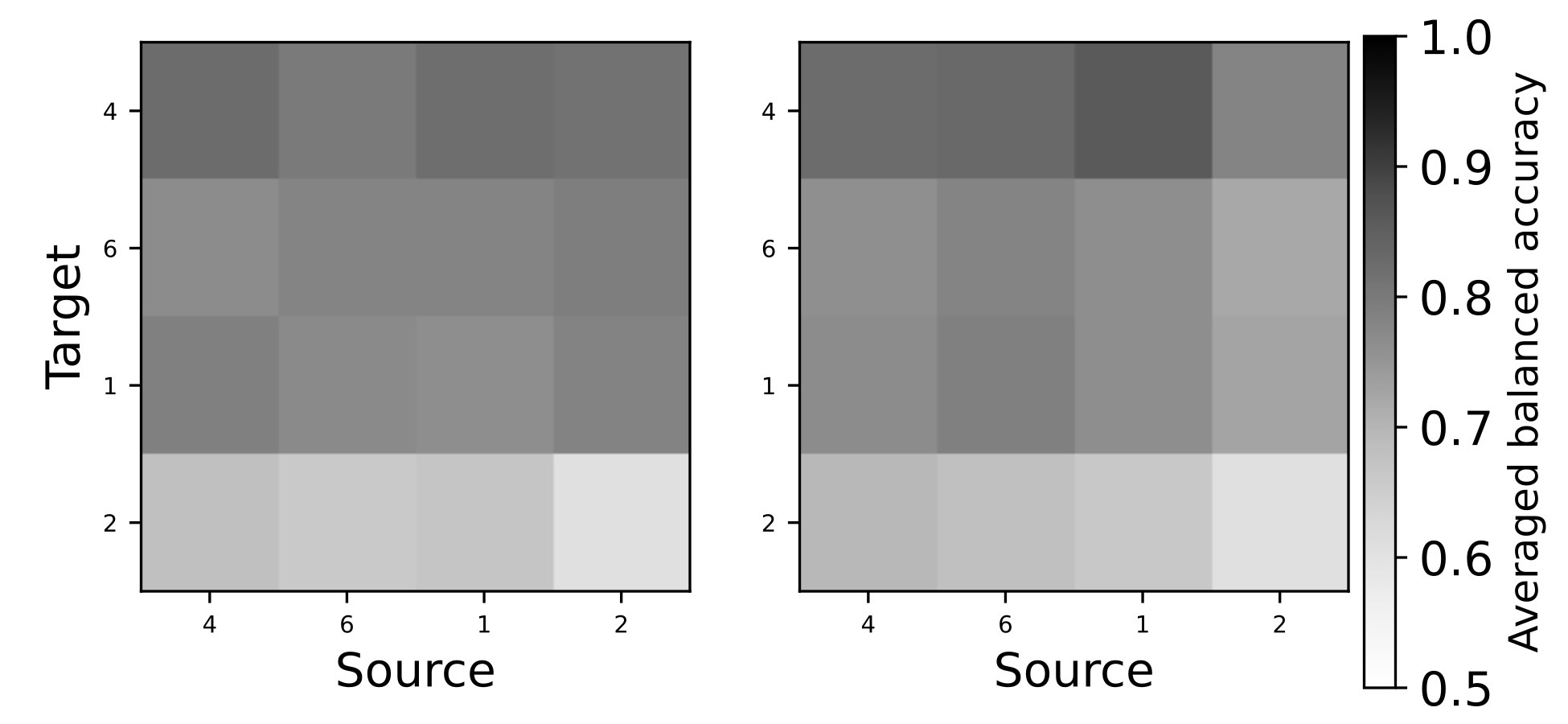

Supplement: Supplementary file 1 [file Data_Sheet_1.ZIP › MI/seriation_both_004-2014_pca3threshold0.60.jpg]

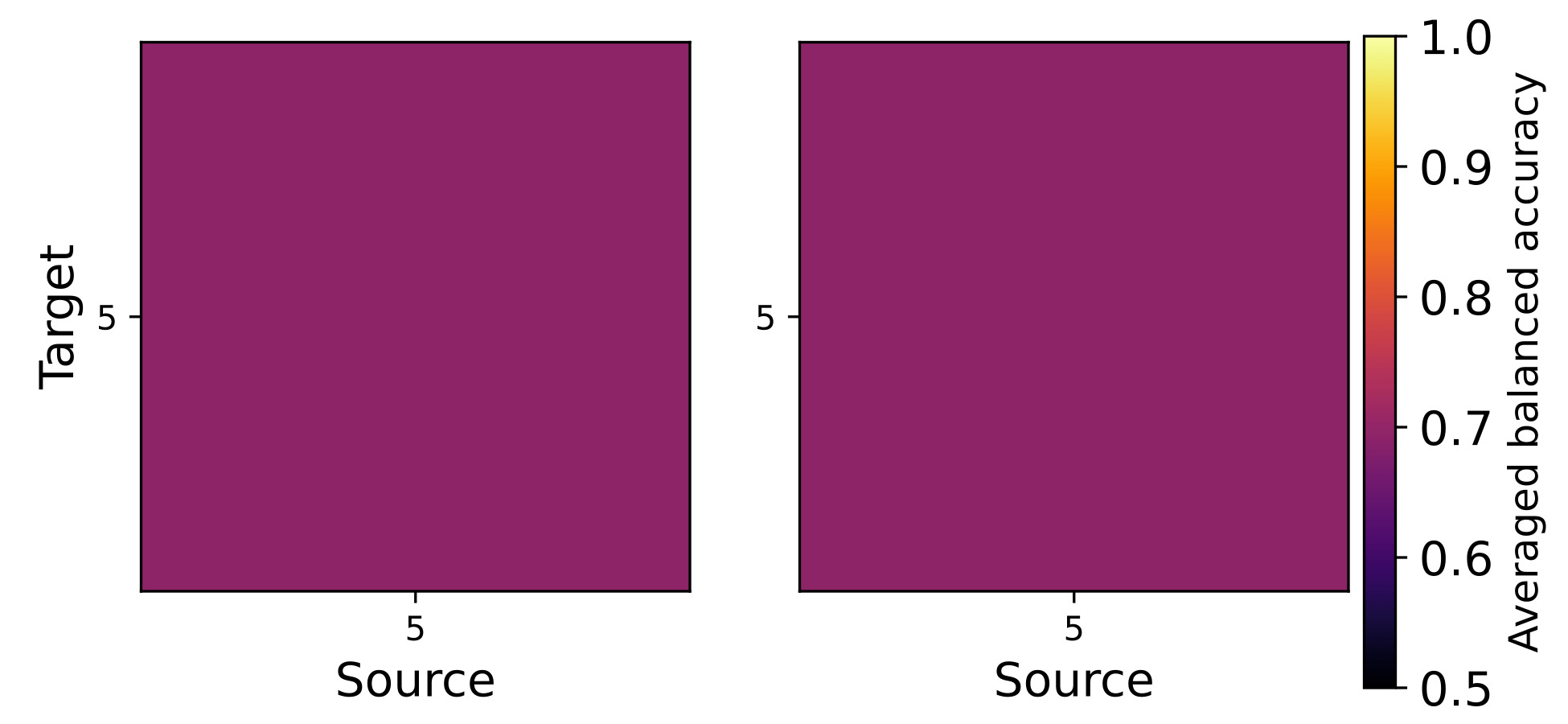

Supplement: Supplementary file 1 [file Data_Sheet_1.ZIP › MI/seriation_both_004-2015_pca3threshold0.60.jpg]

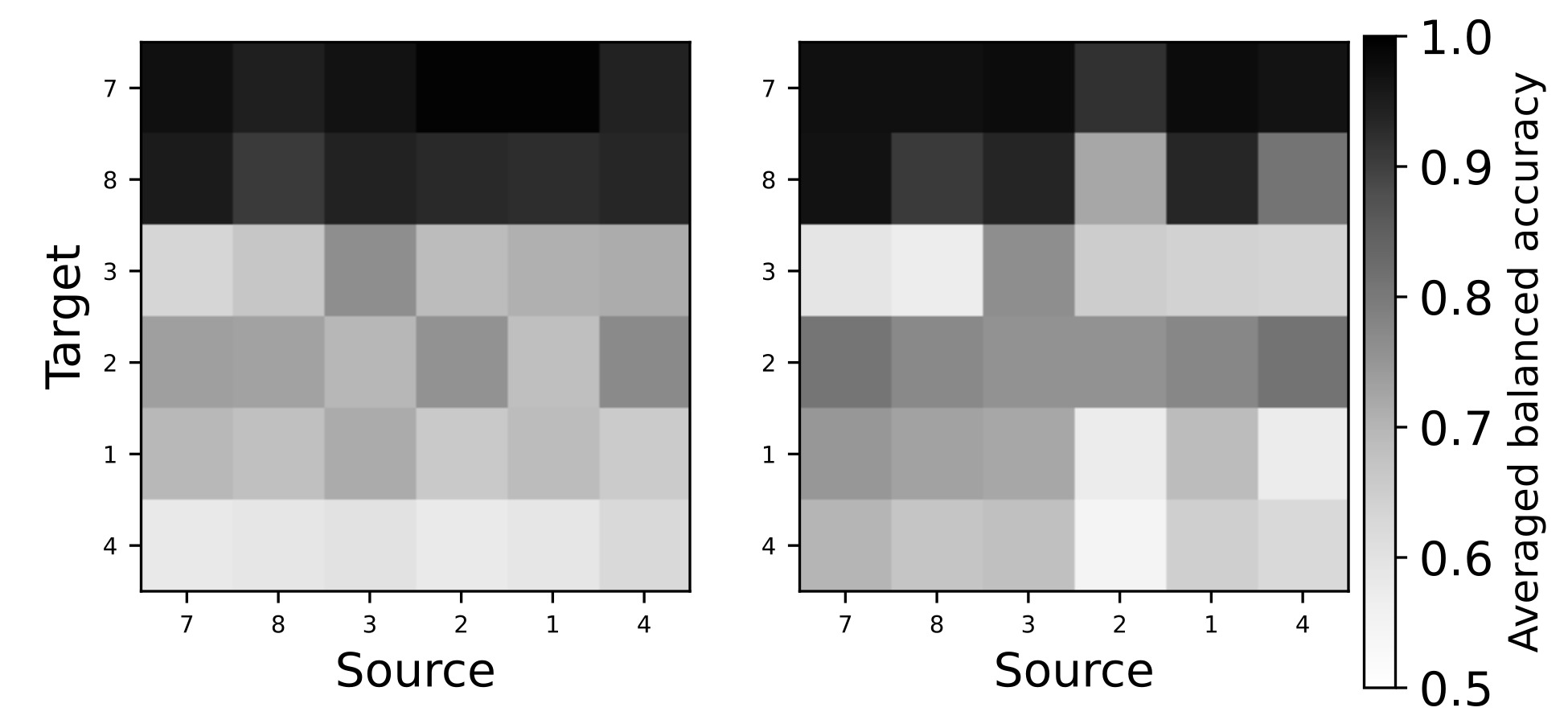

Supplement: Supplementary file 1 [file Data_Sheet_1.ZIP › MI/seriation_both_Alexandre_Motor_Imagery_pca3threshold0.60.jpg]

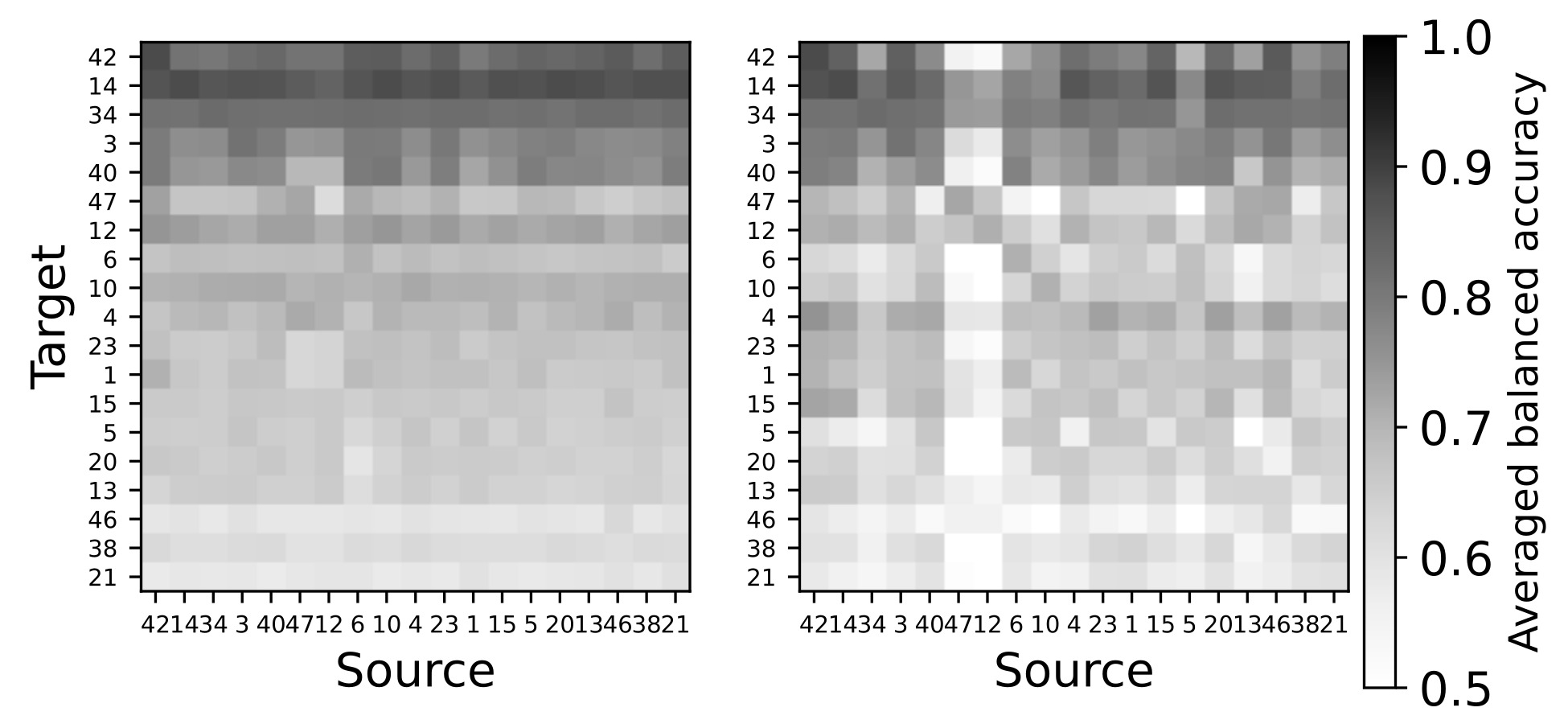

Supplement: Supplementary file 1 [file Data_Sheet_1.ZIP › MI/seriation_both_Cho2017_pca3threshold0.60.jpg]

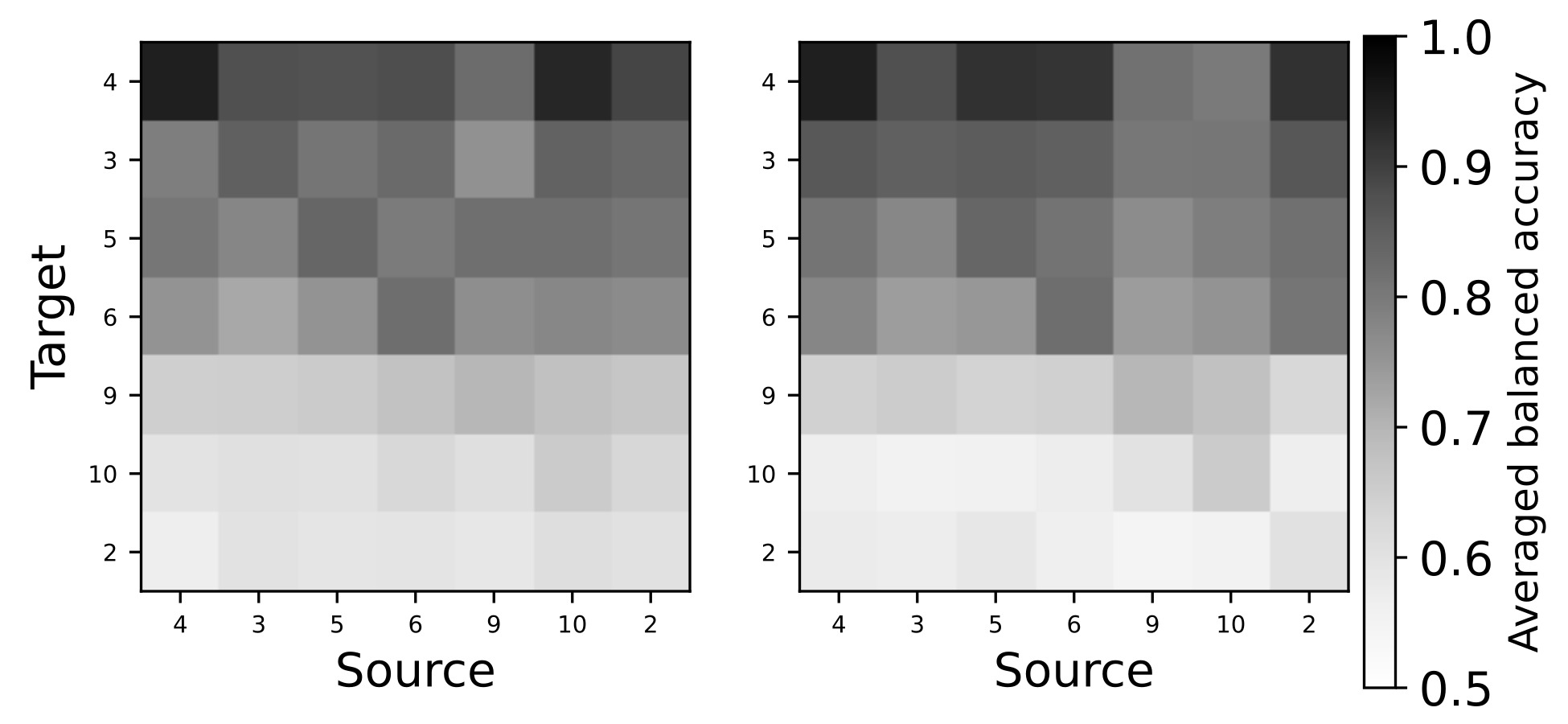

Supplement: Supplementary file 1 [file Data_Sheet_1.ZIP › MI/seriation_both_Grosse-Wentrup_2009_pca3threshold0.60.jpg]

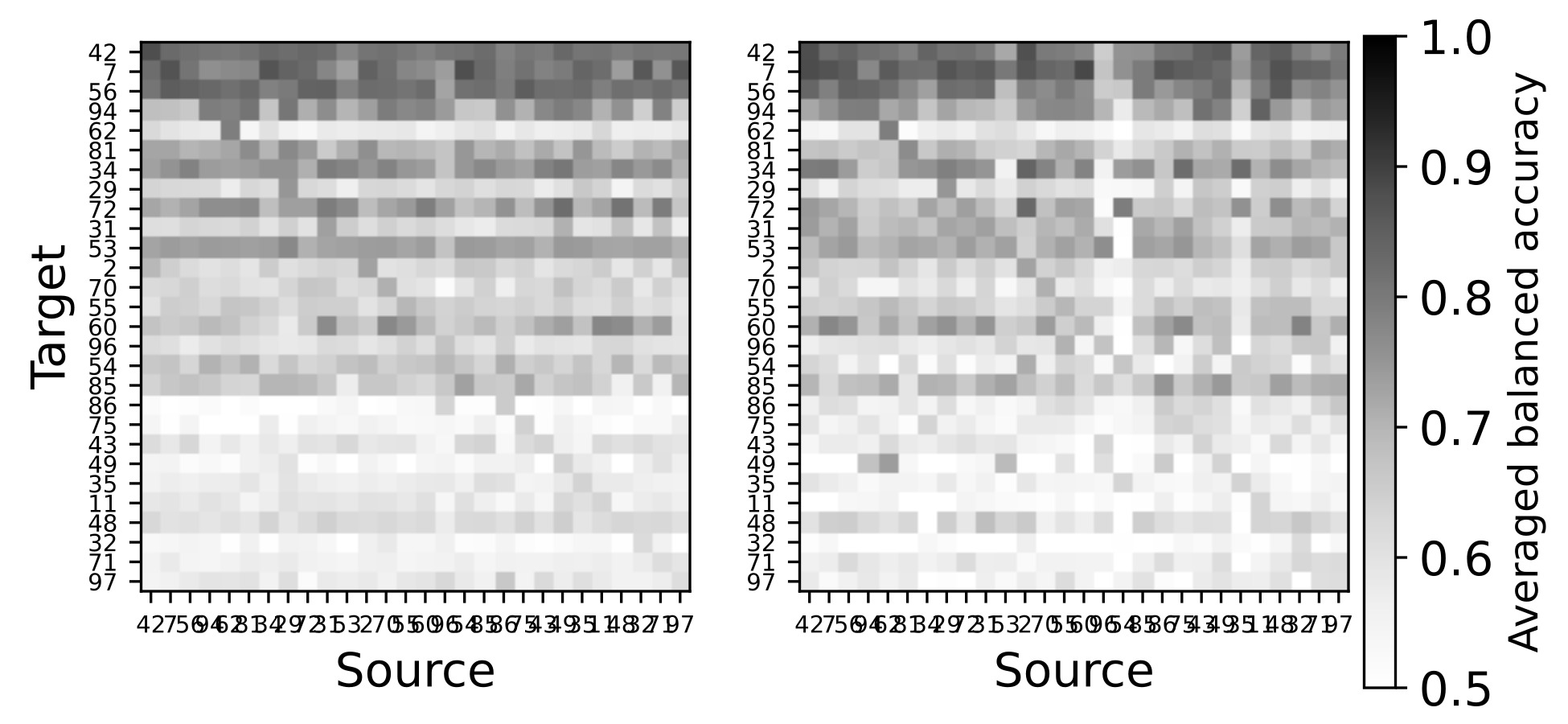

Supplement: Supplementary file 1 [file Data_Sheet_1.ZIP › MI/seriation_both_Physionet_Motor_Imagery_pca3threshold0.60.jpg]

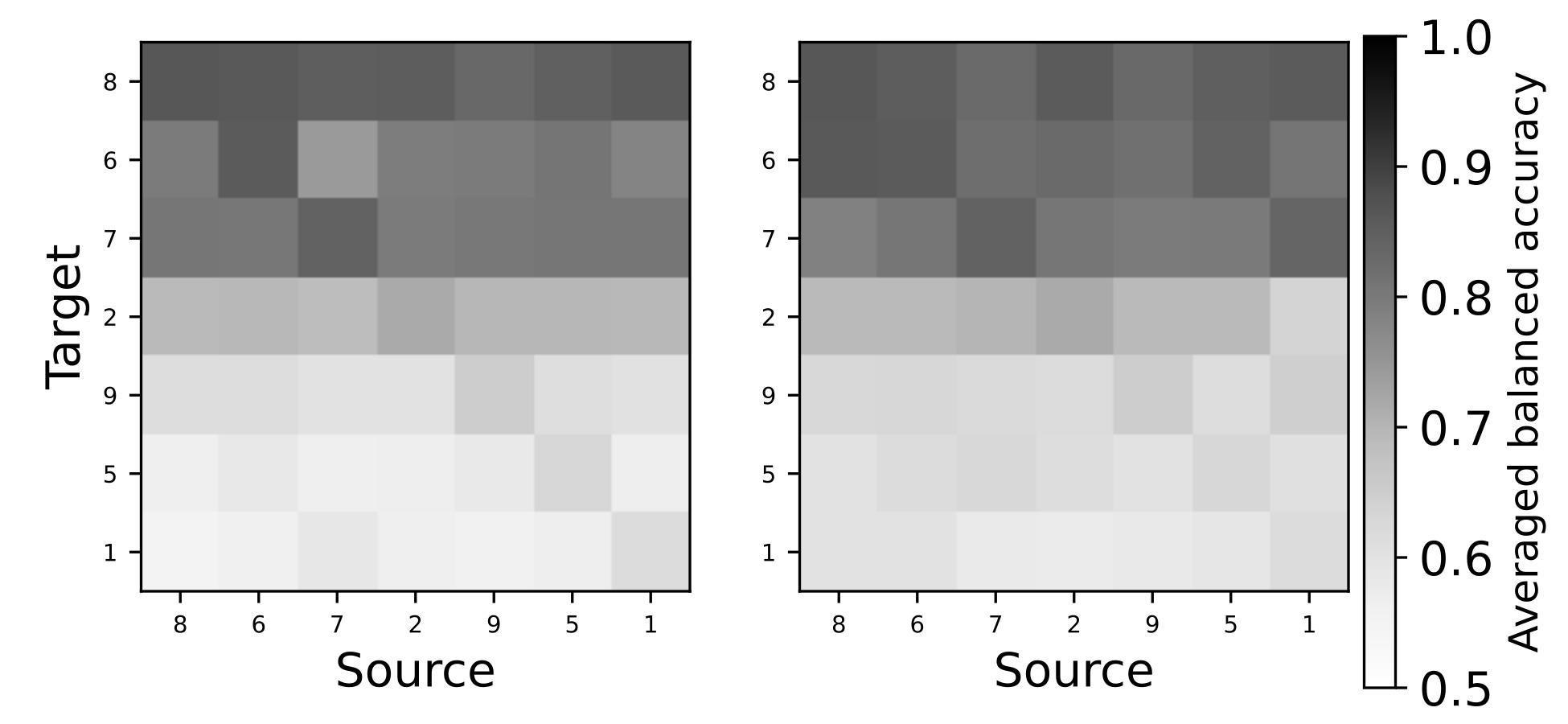

Supplement: Supplementary file 1 [file Data_Sheet_1.ZIP › MI/seriation_both_Weibo_2014_pca3threshold0.60.jpg]

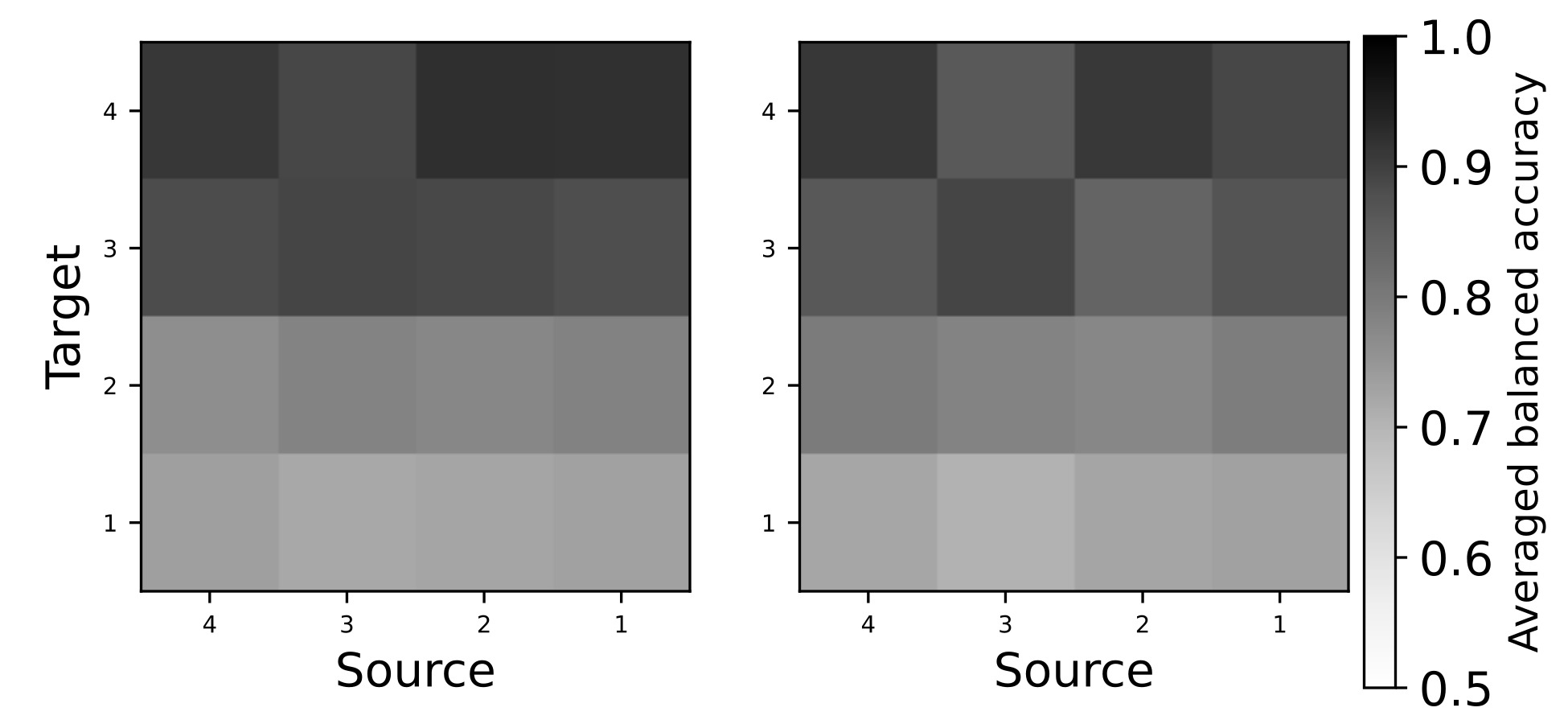

Supplement: Supplementary file 1 [file Data_Sheet_1.ZIP › MI/seriation_both_Zhou_2016_pca3threshold0.60.jpg]

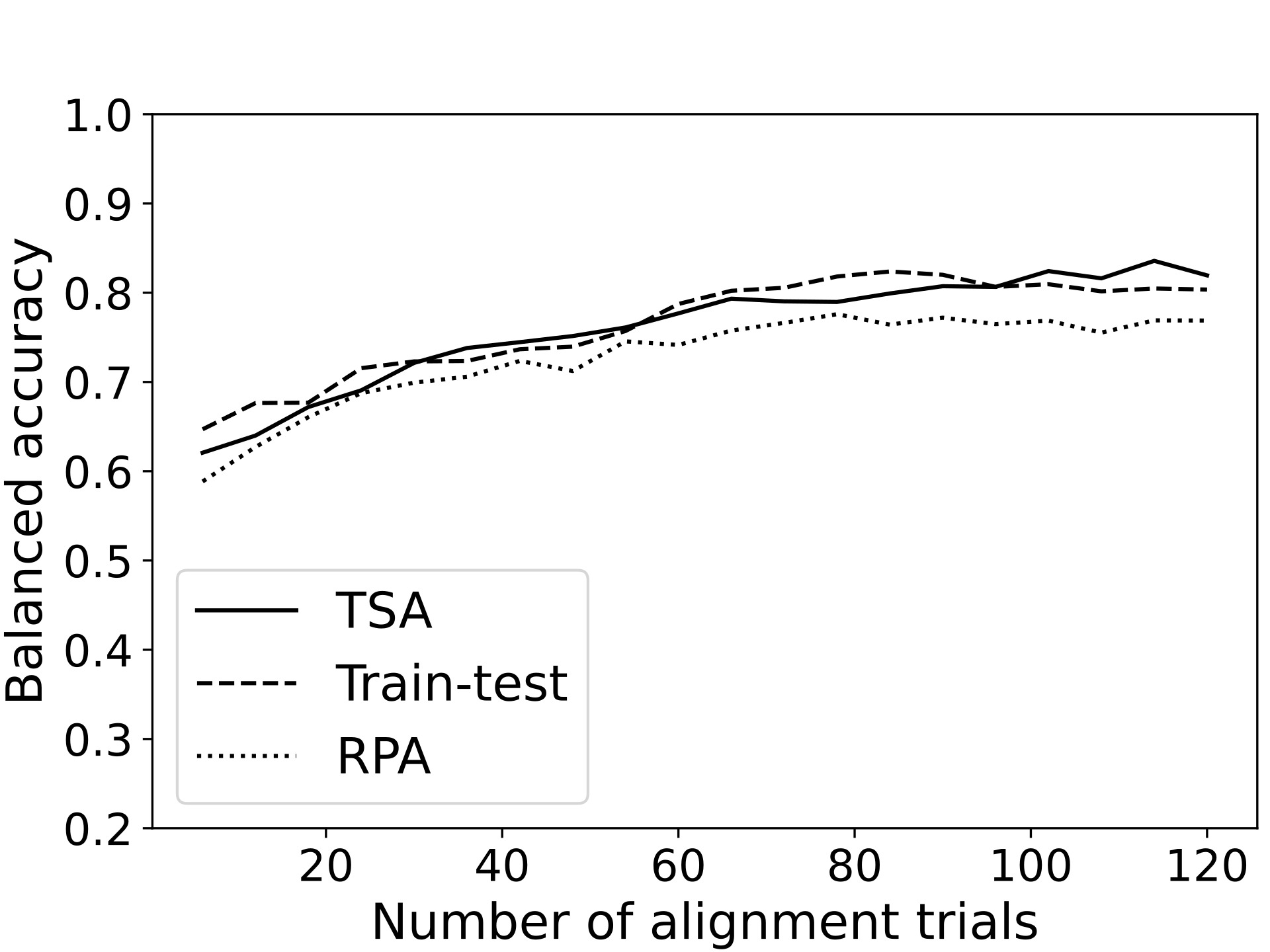

Supplement: Supplementary file 1 [file Data_Sheet_1.ZIP › MI/accuracy_001-2014_pca3threshold0.60.jpg]

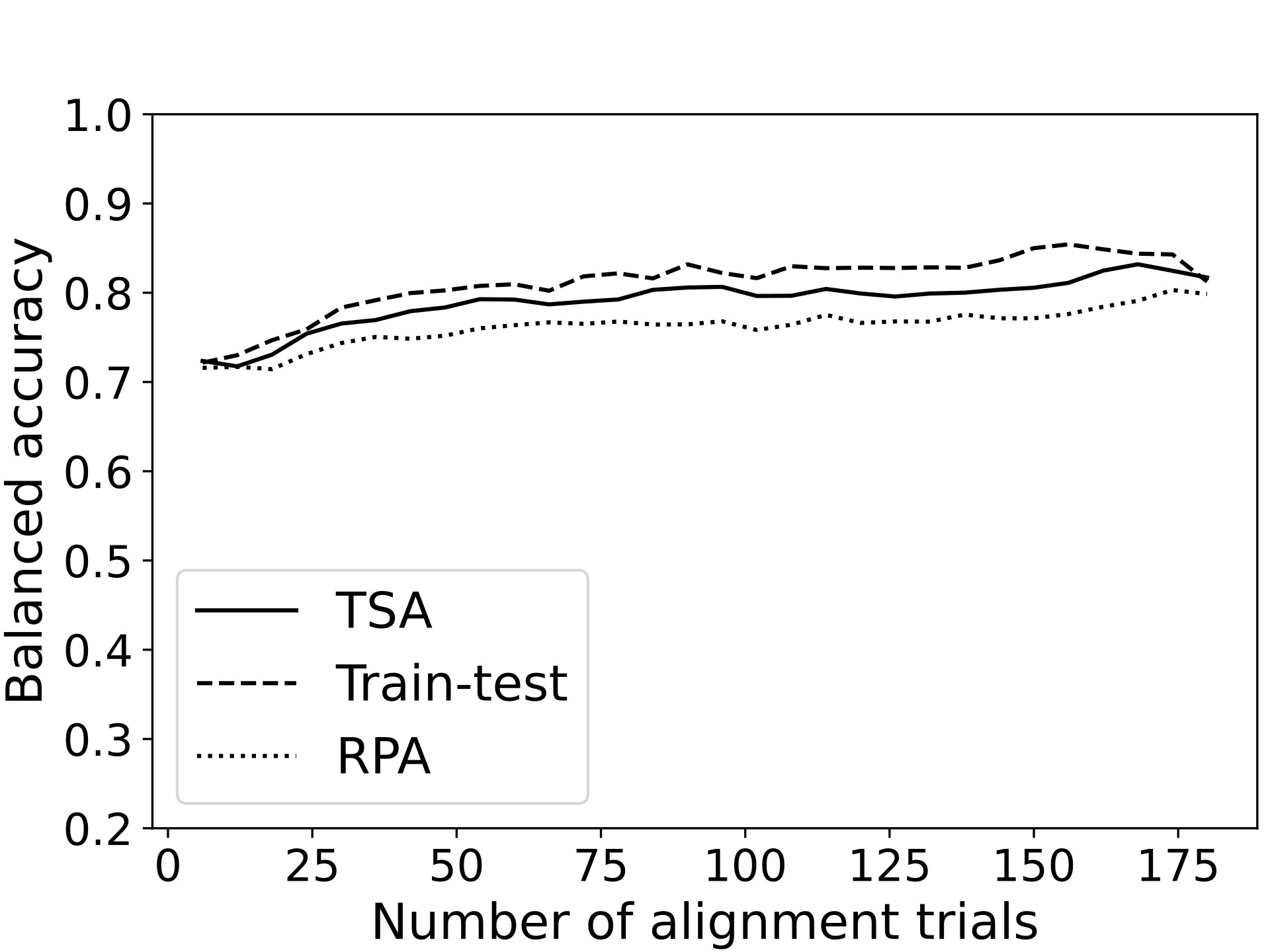

Supplement: Supplementary file 1 [file Data_Sheet_1.ZIP › MI/accuracy_001-2015_pca3threshold0.60.jpg]

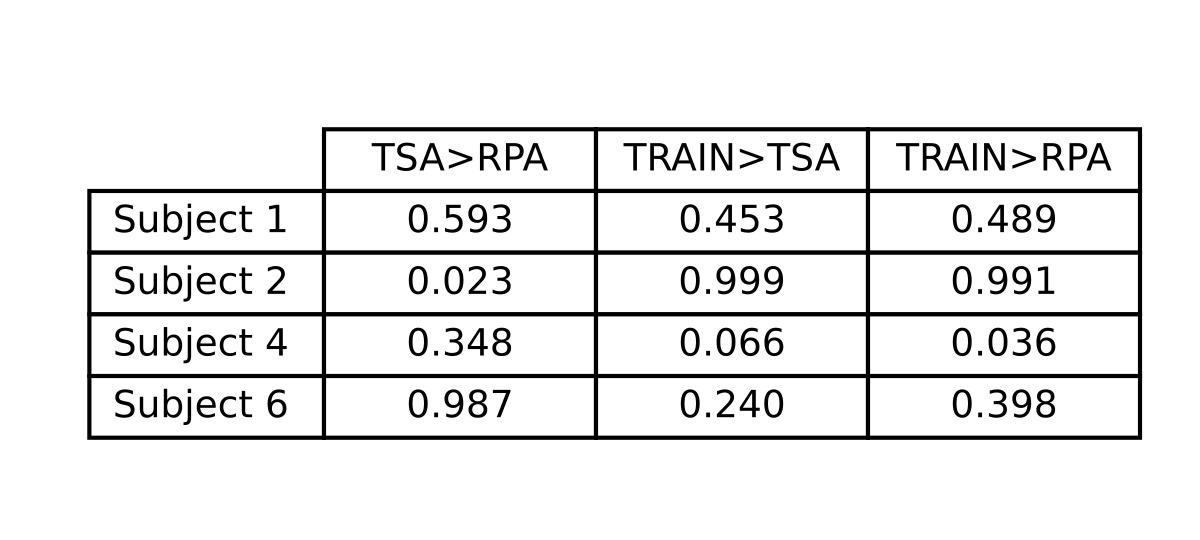

Supplement: Supplementary file 1 [file Data_Sheet_1.ZIP › MI/004-2014_threshold_0.60.jpg]

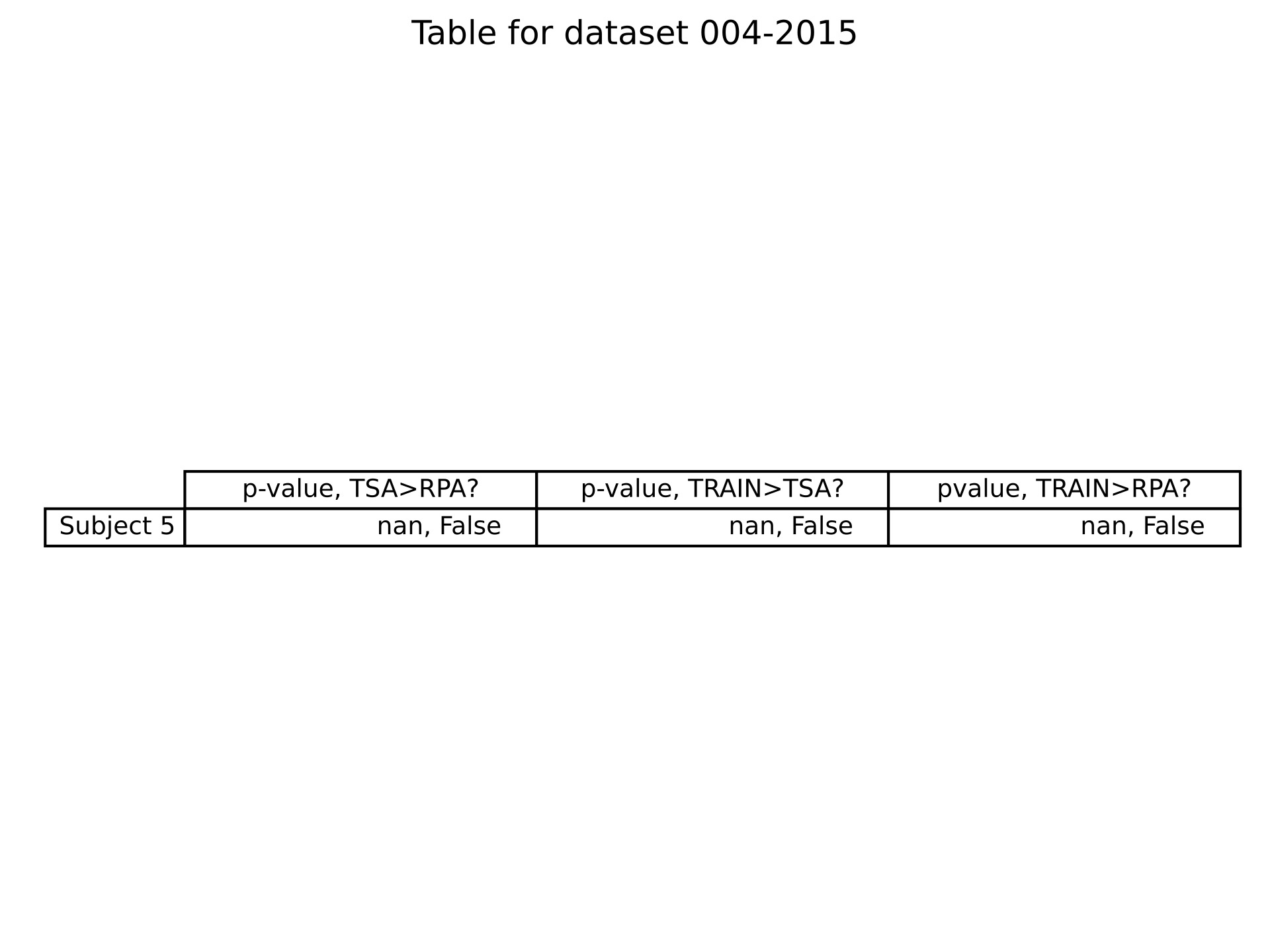

Supplement: Supplementary file 1 [file Data_Sheet_1.ZIP › MI/004-2015_threshold_0.60.jpg]

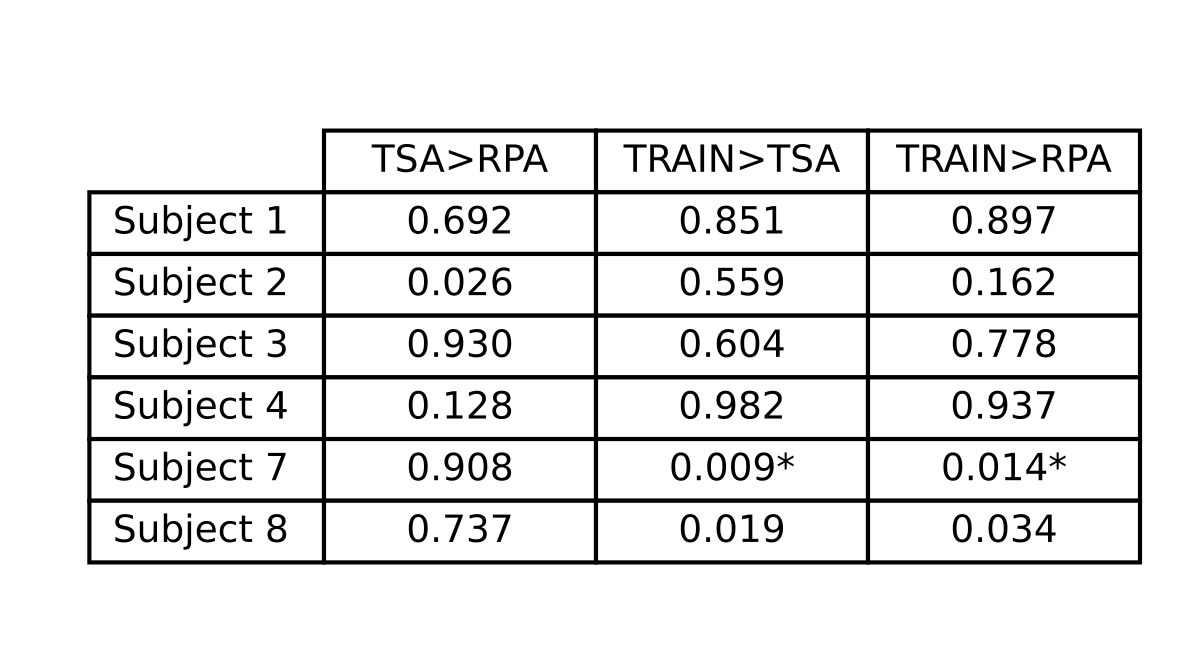

Supplement: Supplementary file 1 [file Data_Sheet_1.ZIP › MI/Alexandre_Motor_Imagery_threshold_0.60.jpg]

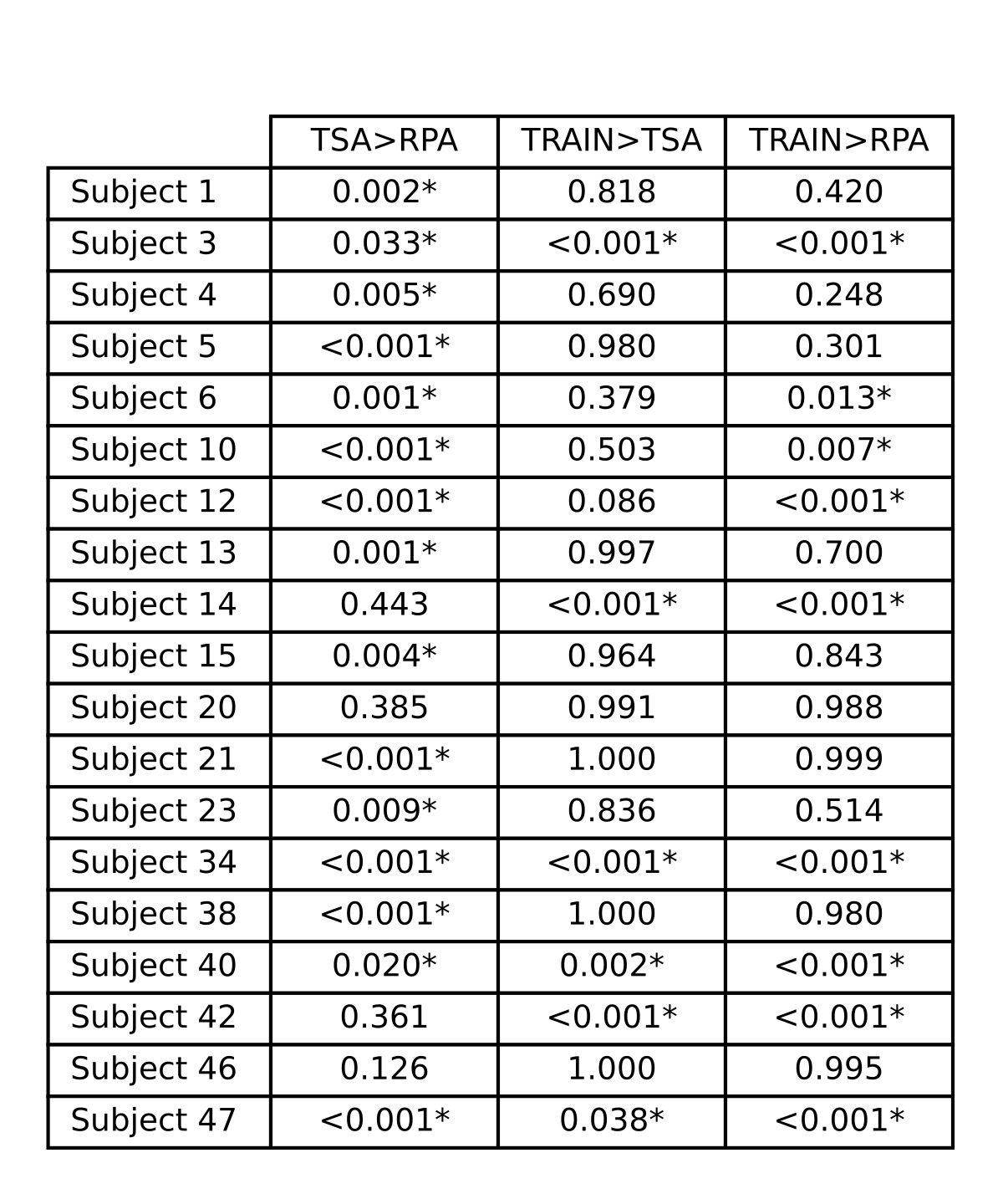

Supplement: Supplementary file 1 [file Data_Sheet_1.ZIP › MI/Cho2017_threshold_0.60.jpg]

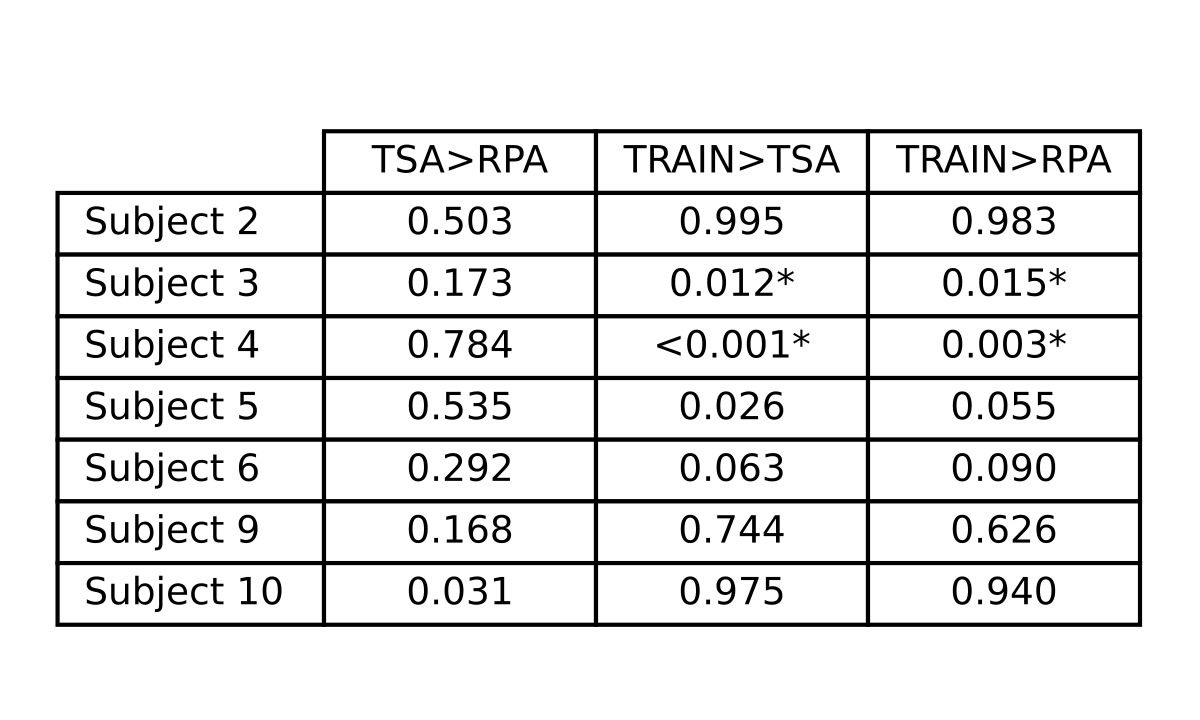

Supplement: Supplementary file 1 [file Data_Sheet_1.ZIP › MI/Grosse-Wentrup_2009_threshold_0.60.jpg]

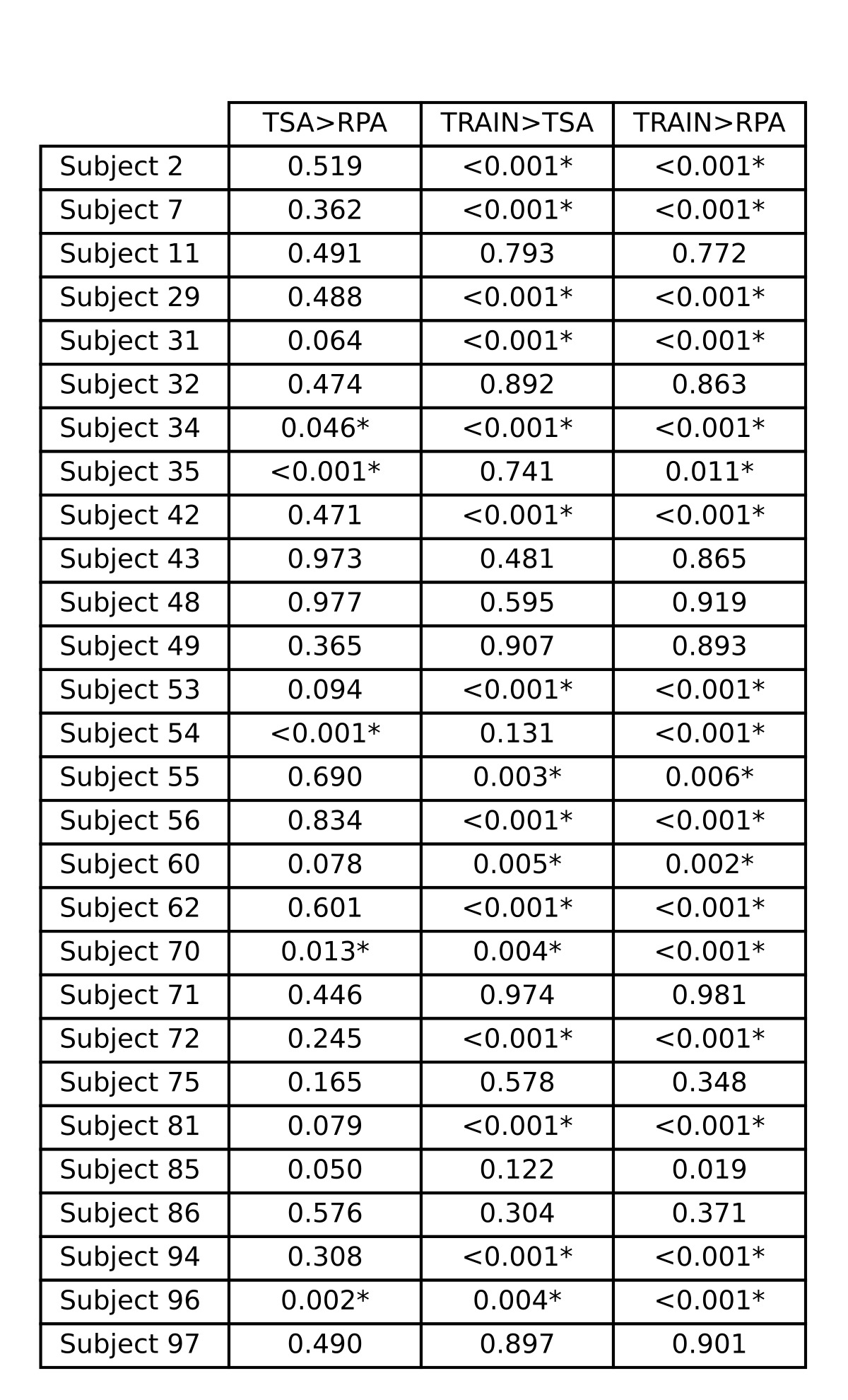

Supplement: Supplementary file 1 [file Data_Sheet_1.ZIP › MI/Physionet_Motor_Imagery_threshold_0.60.jpg]

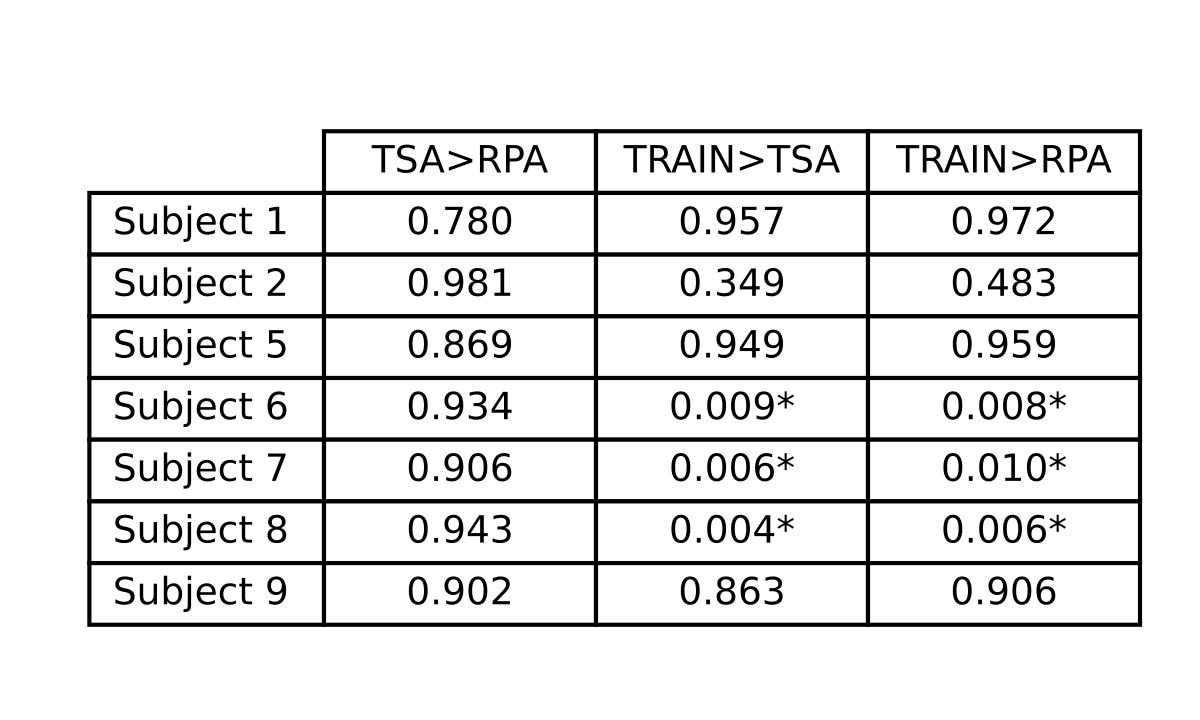

Supplement: Supplementary file 1 [file Data_Sheet_1.ZIP › MI/Weibo_2014_threshold_0.60.jpg]

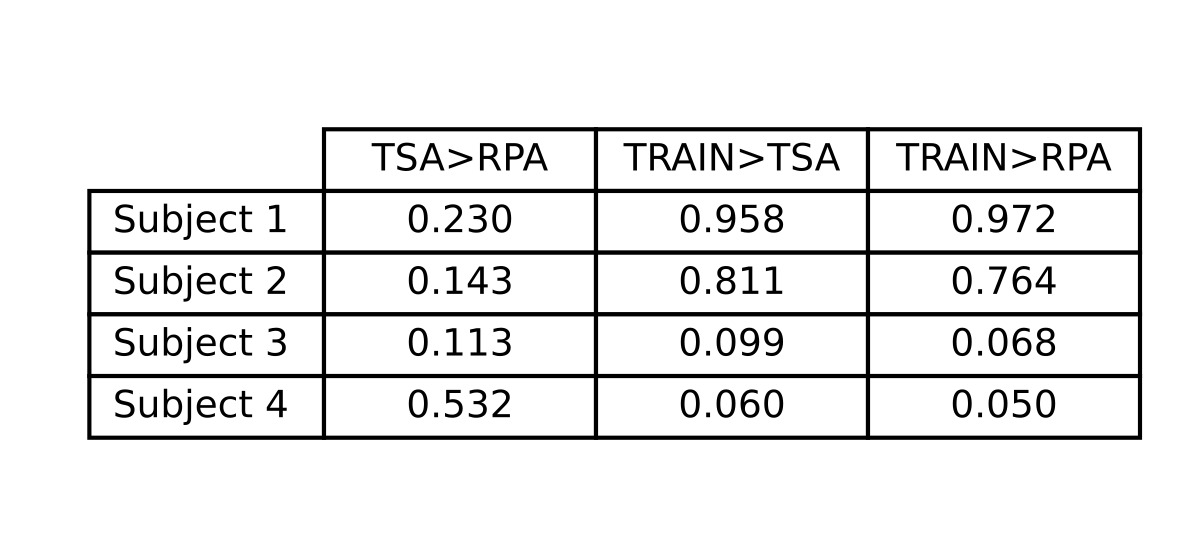

Supplement: Supplementary file 1 [file Data_Sheet_1.ZIP › MI/Zhou_2016_threshold_0.60.jpg]

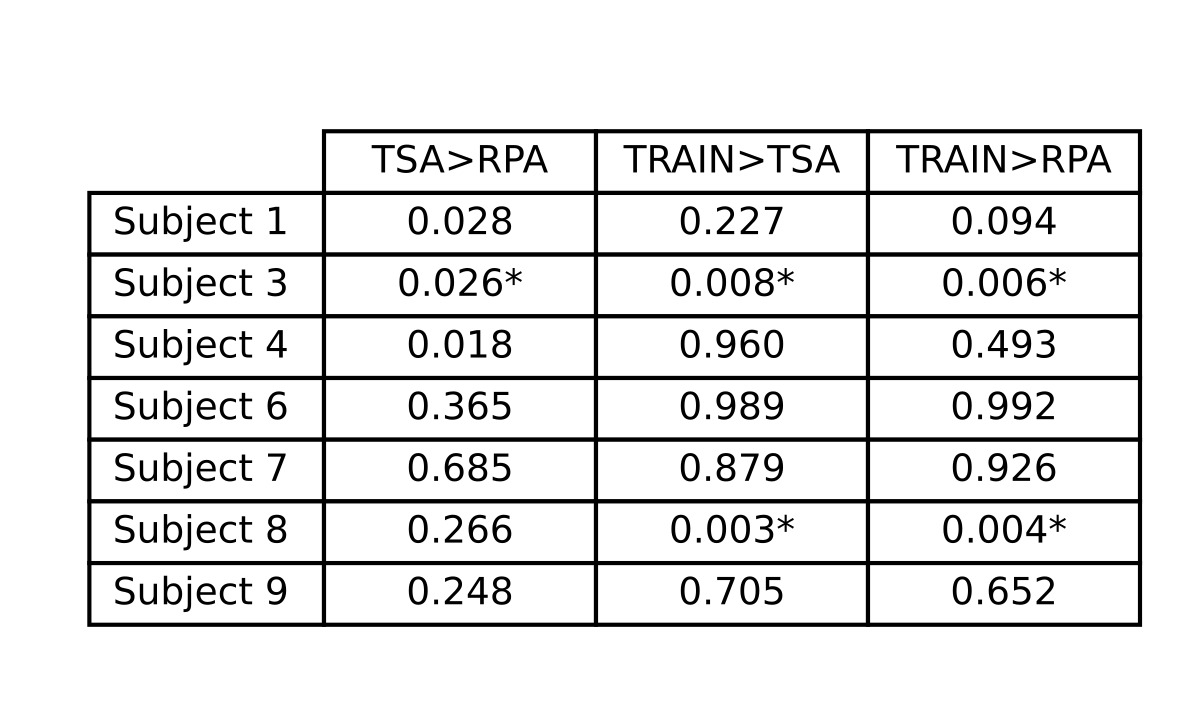

Supplement: Supplementary file 1 [file Data_Sheet_1.ZIP › MI/001-2014_threshold_0.60.jpg]

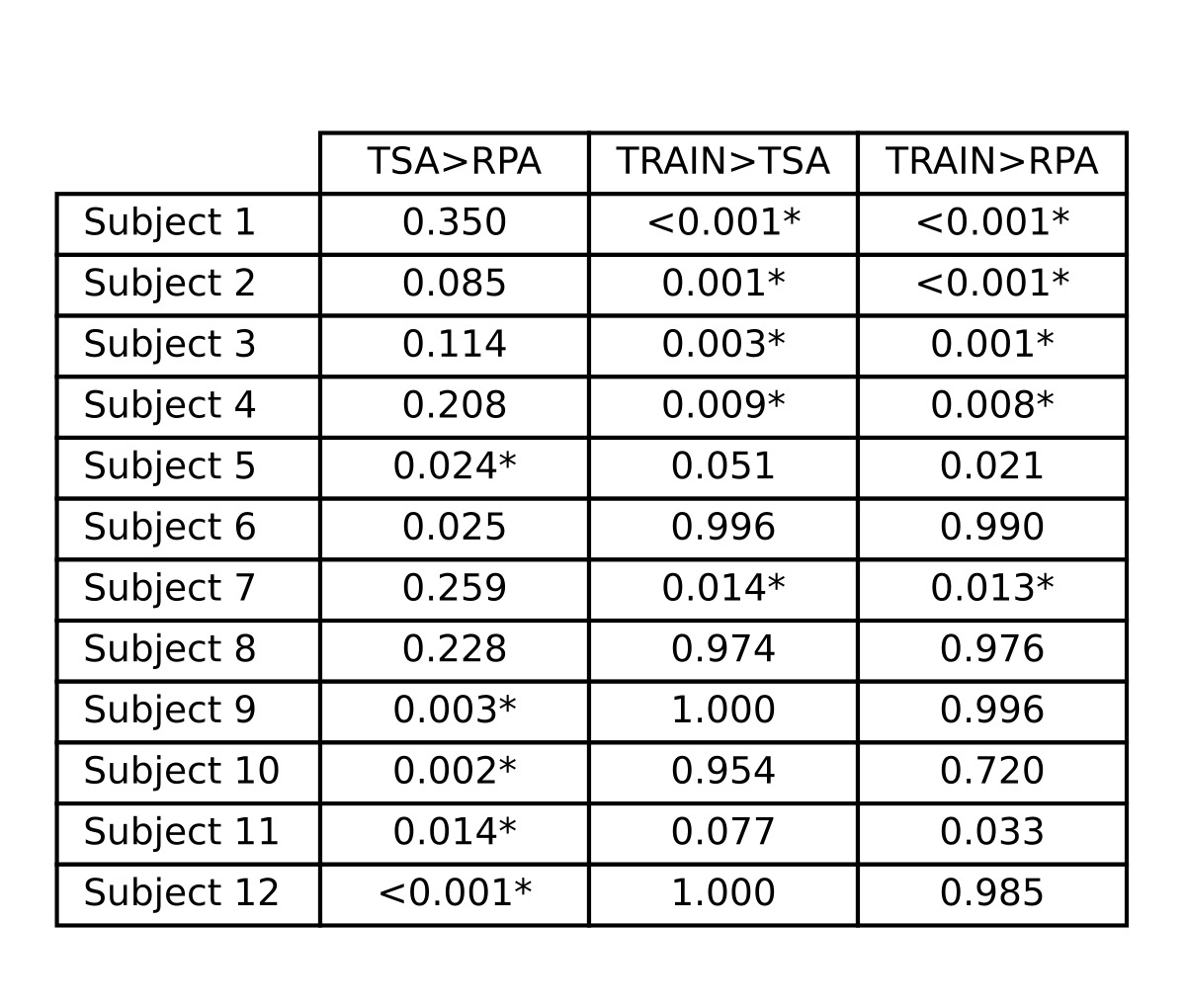

Supplement: Supplementary file 1 [file Data_Sheet_1.ZIP › MI/001-2015_threshold_0.60.jpg]

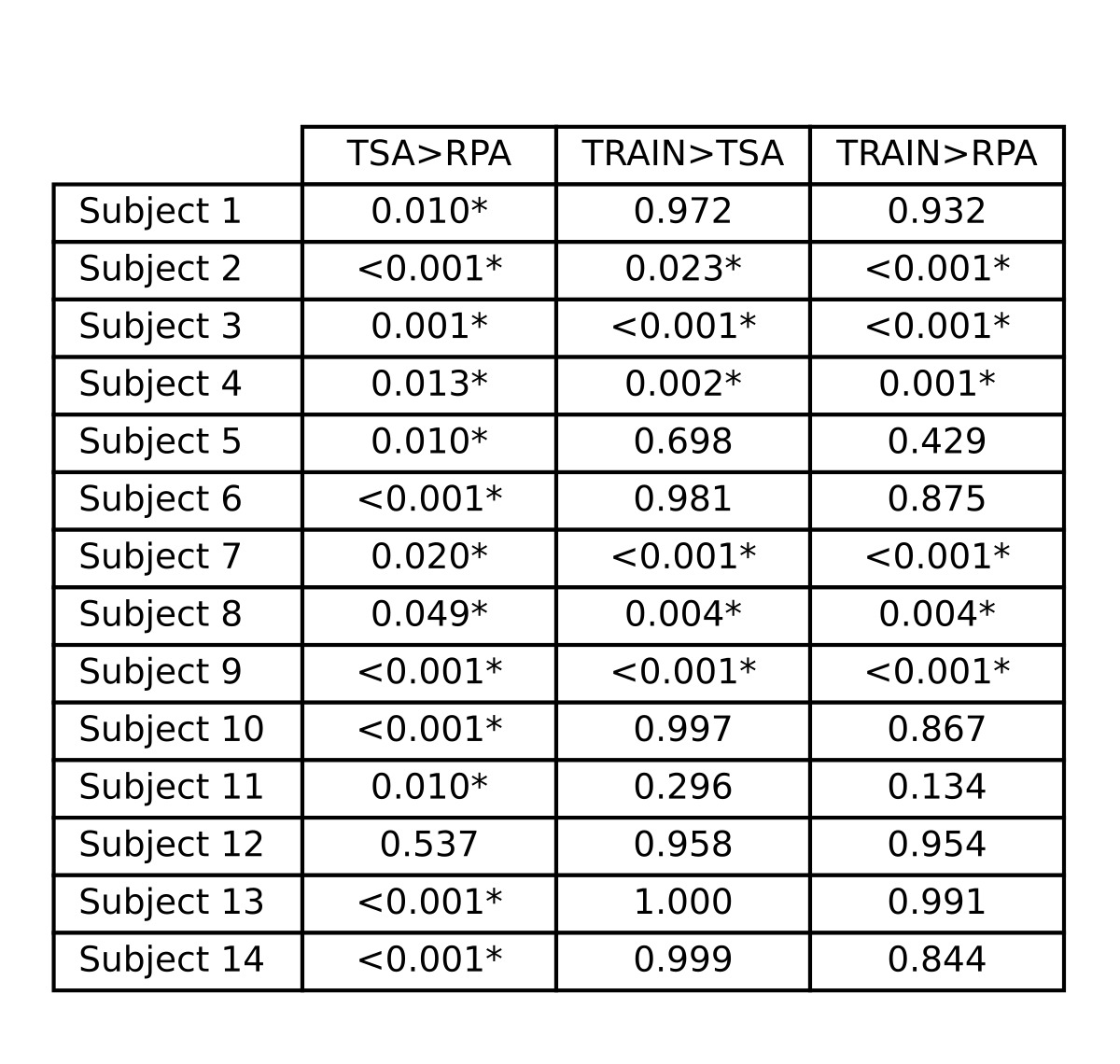

Supplement: Supplementary file 1 [file Data_Sheet_1.ZIP › MI/002-2014_threshold_0.60.jpg]

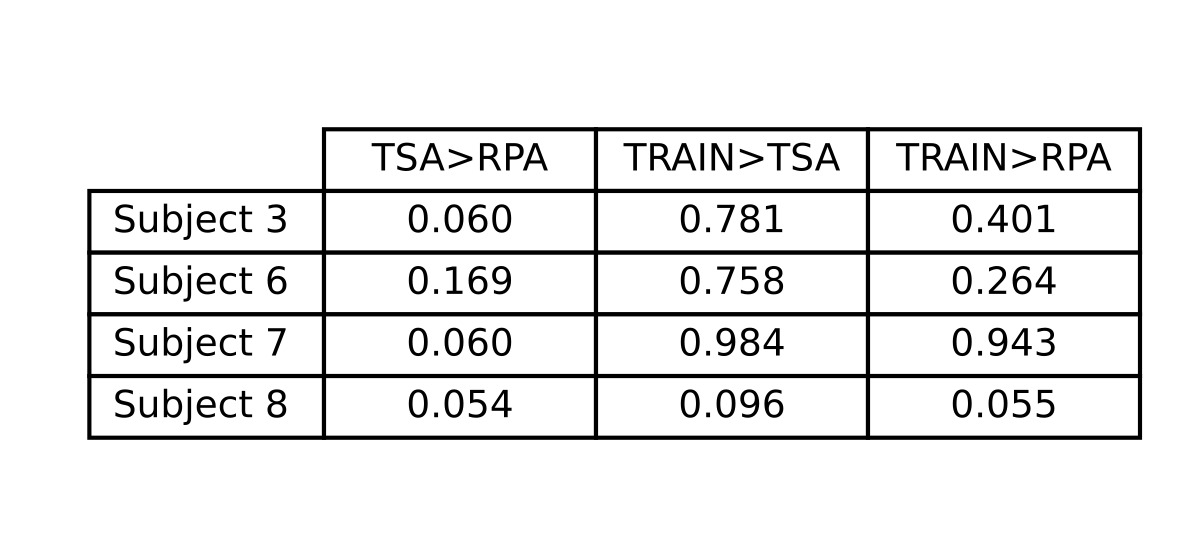

Supplement: Supplementary file 1 [file Data_Sheet_1.ZIP › ERP/008-2014_threshold_0.60.jpg]

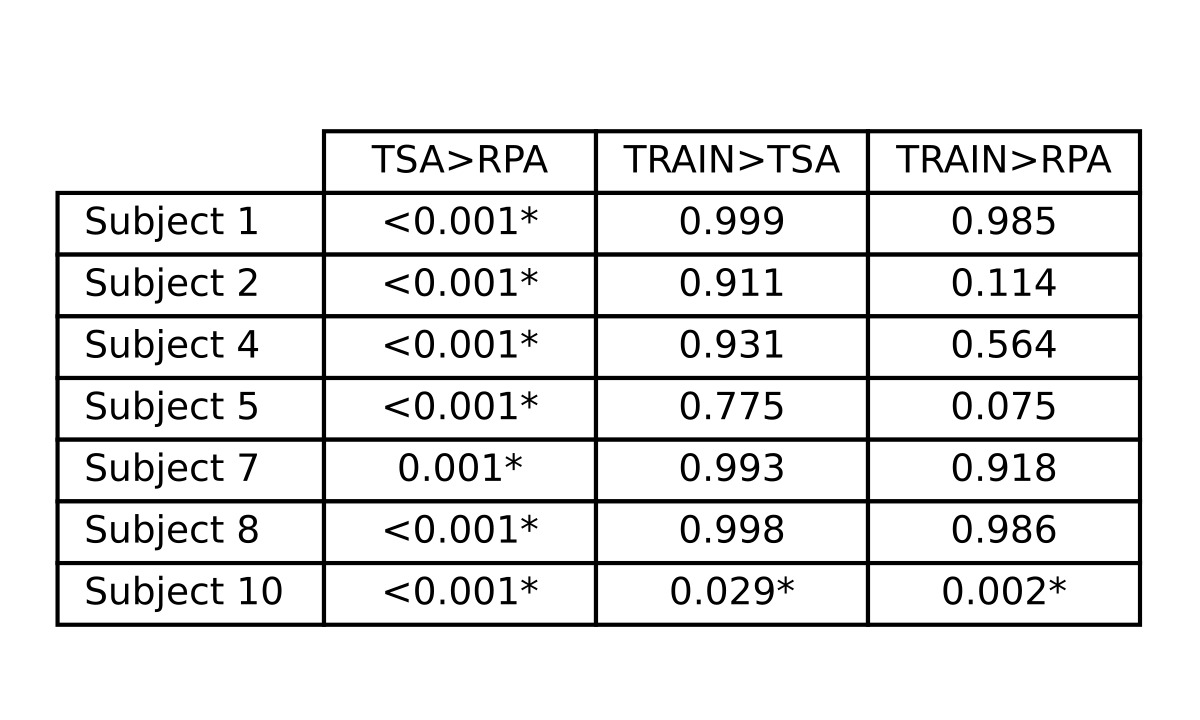

Supplement: Supplementary file 1 [file Data_Sheet_1.ZIP › ERP/009-2014_threshold_0.60.jpg]

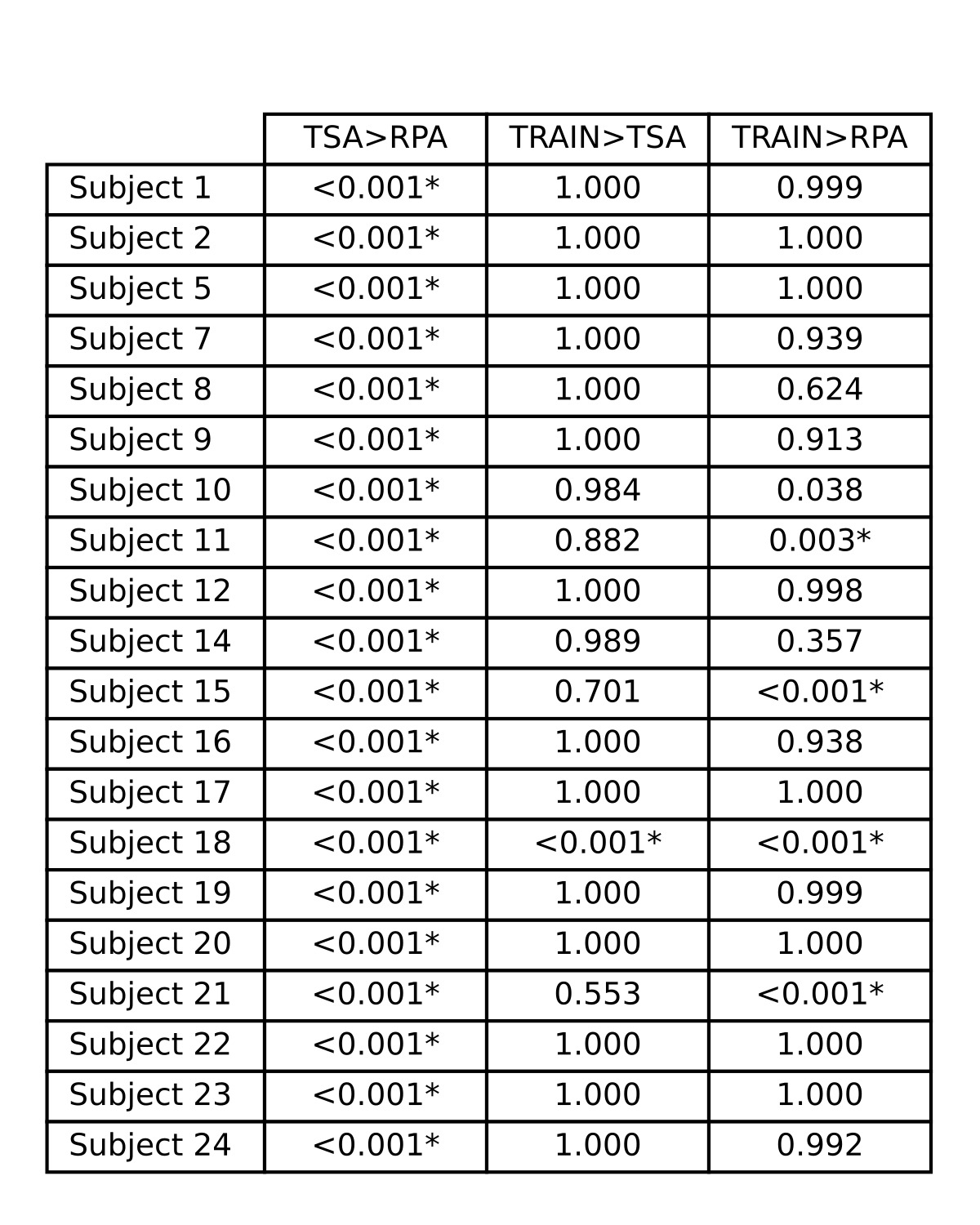

Supplement: Supplementary file 1 [file Data_Sheet_1.ZIP › ERP/Brain_Invaders_2013a_threshold_0.60.jpg]

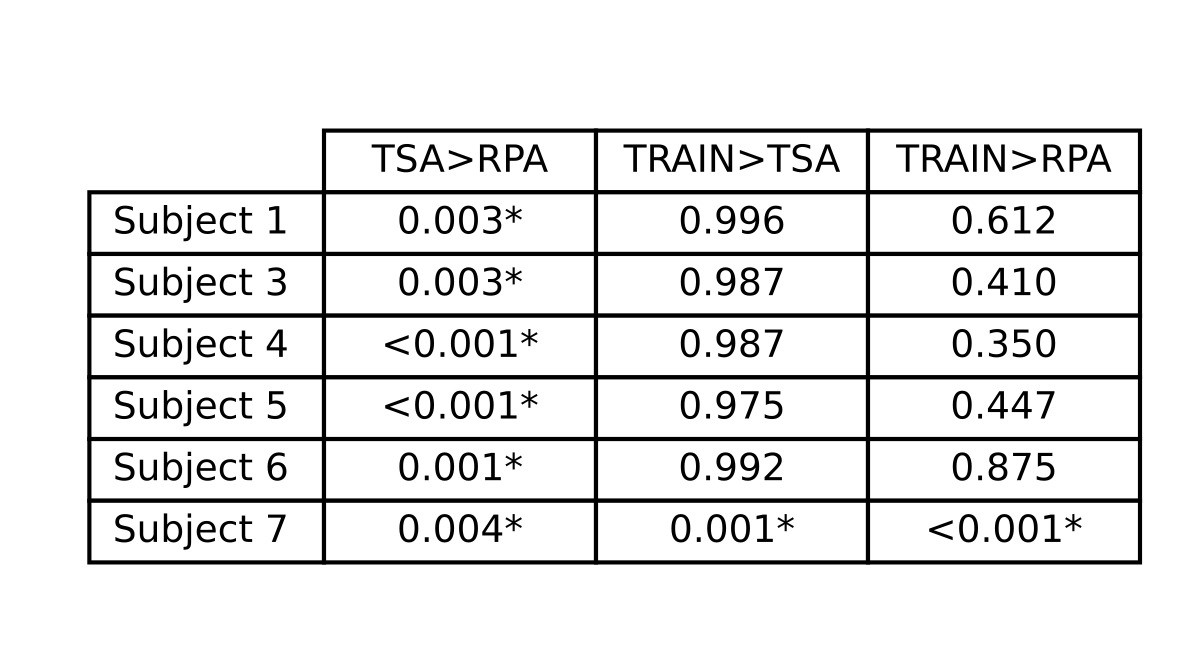

Supplement: Supplementary file 1 [file Data_Sheet_1.ZIP › ERP/EPFL_P300_dataset_threshold_0.60.jpg]

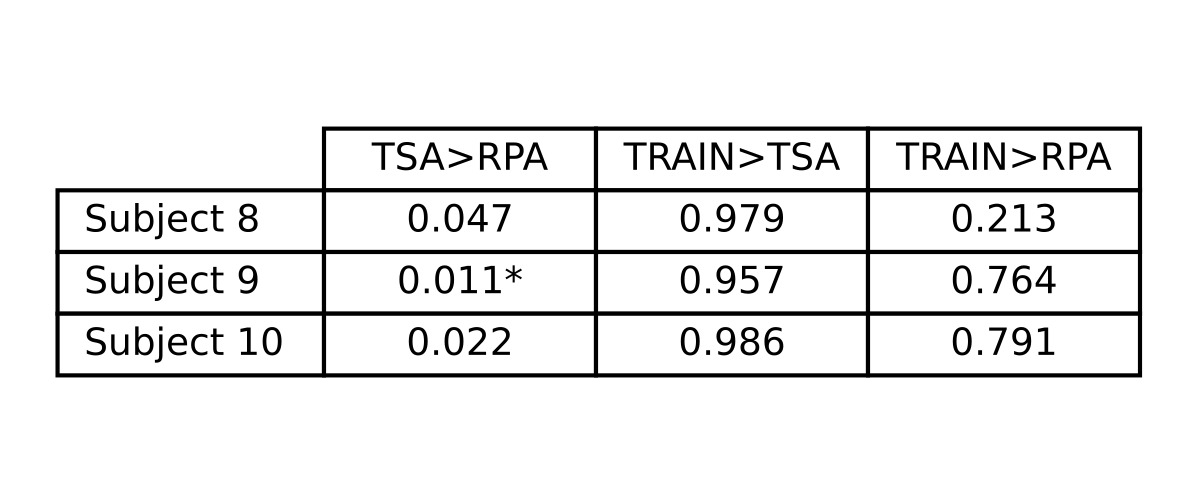

Supplement: Supplementary file 1 [file Data_Sheet_1.ZIP › ERP/003-2015_threshold_0.60.jpg]

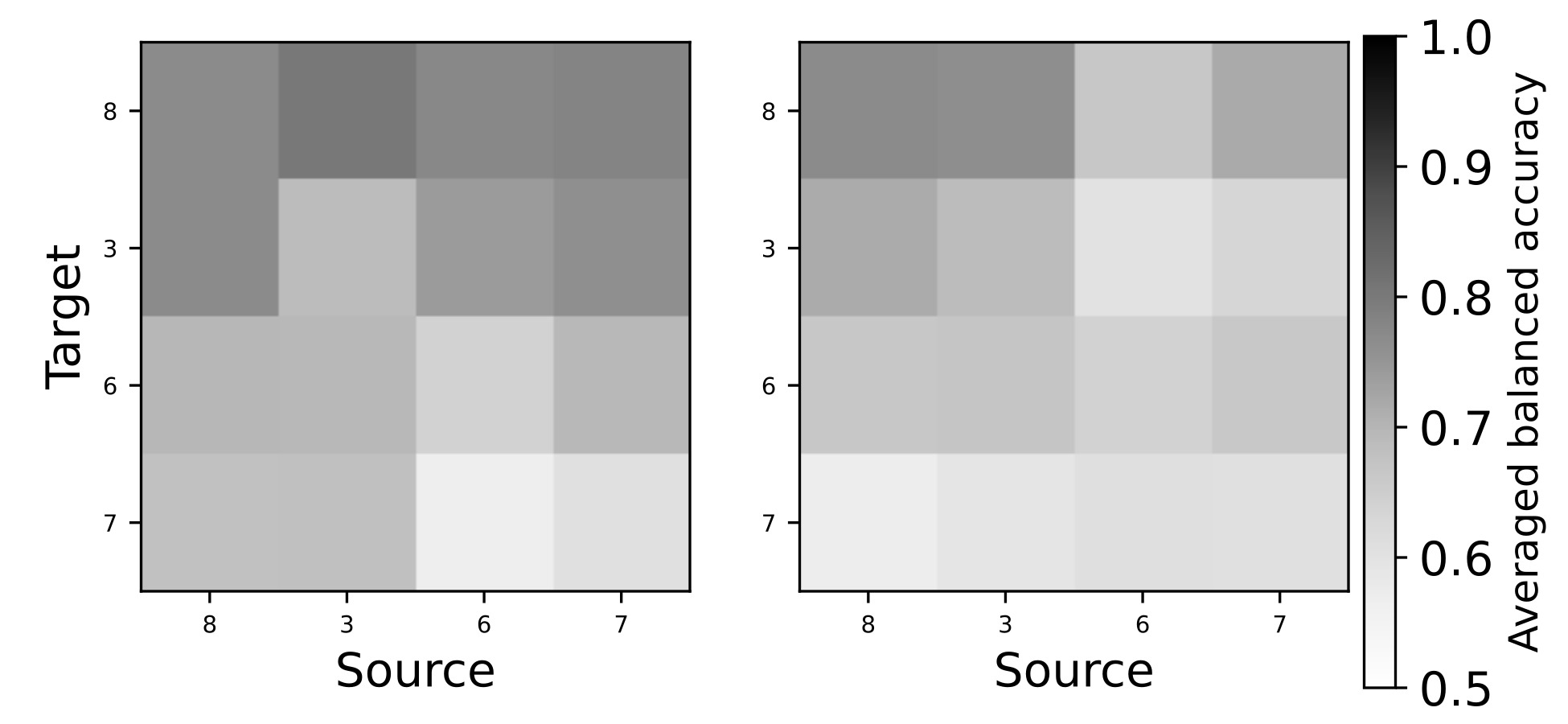

Supplement: Supplementary file 1 [file Data_Sheet_1.ZIP › ERP/seriation_both_008-2014_pca3threshold0.60.jpg]

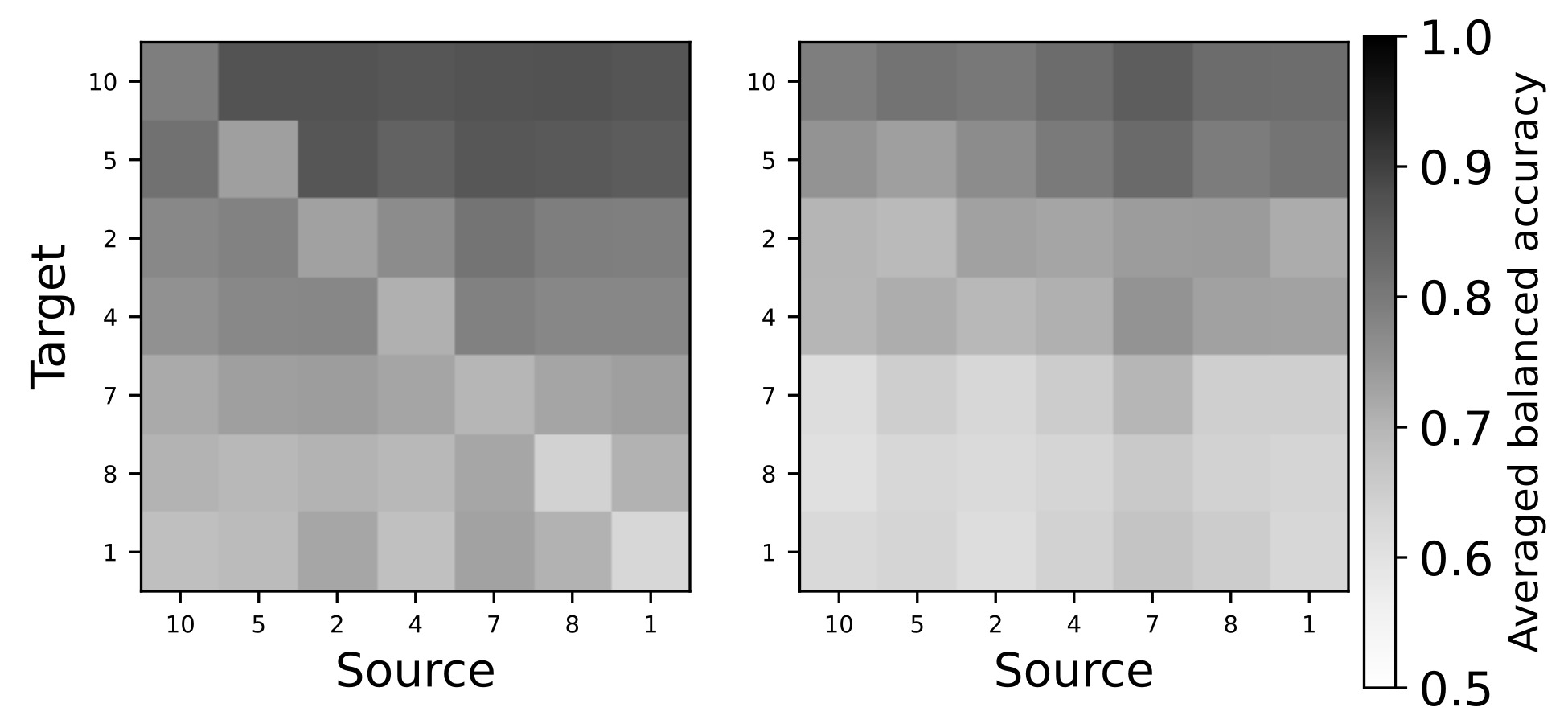

Supplement: Supplementary file 1 [file Data_Sheet_1.ZIP › ERP/seriation_both_009-2014_pca3threshold0.60.jpg]

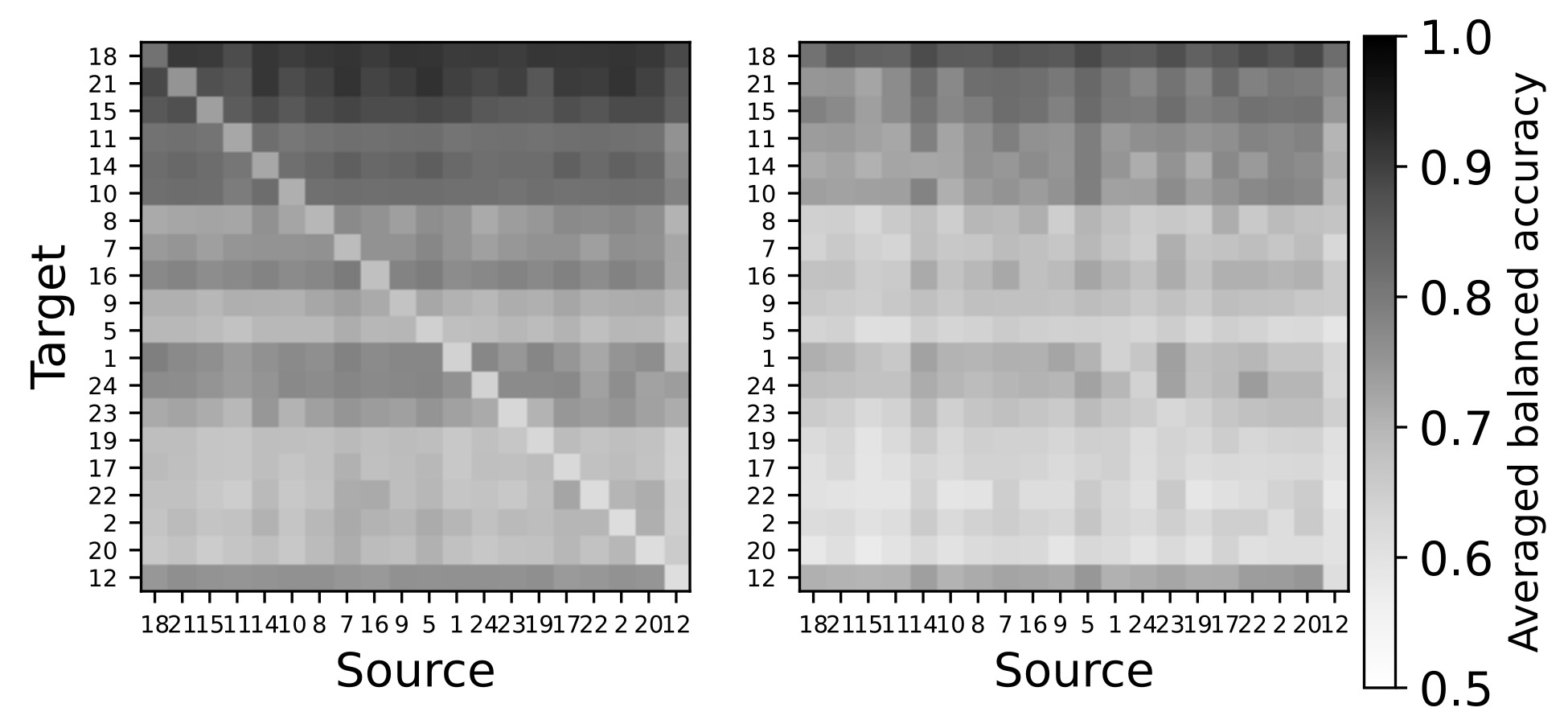

Supplement: Supplementary file 1 [file Data_Sheet_1.ZIP › ERP/seriation_both_Brain_Invaders_2013a_pca3threshold0.60.jpg]

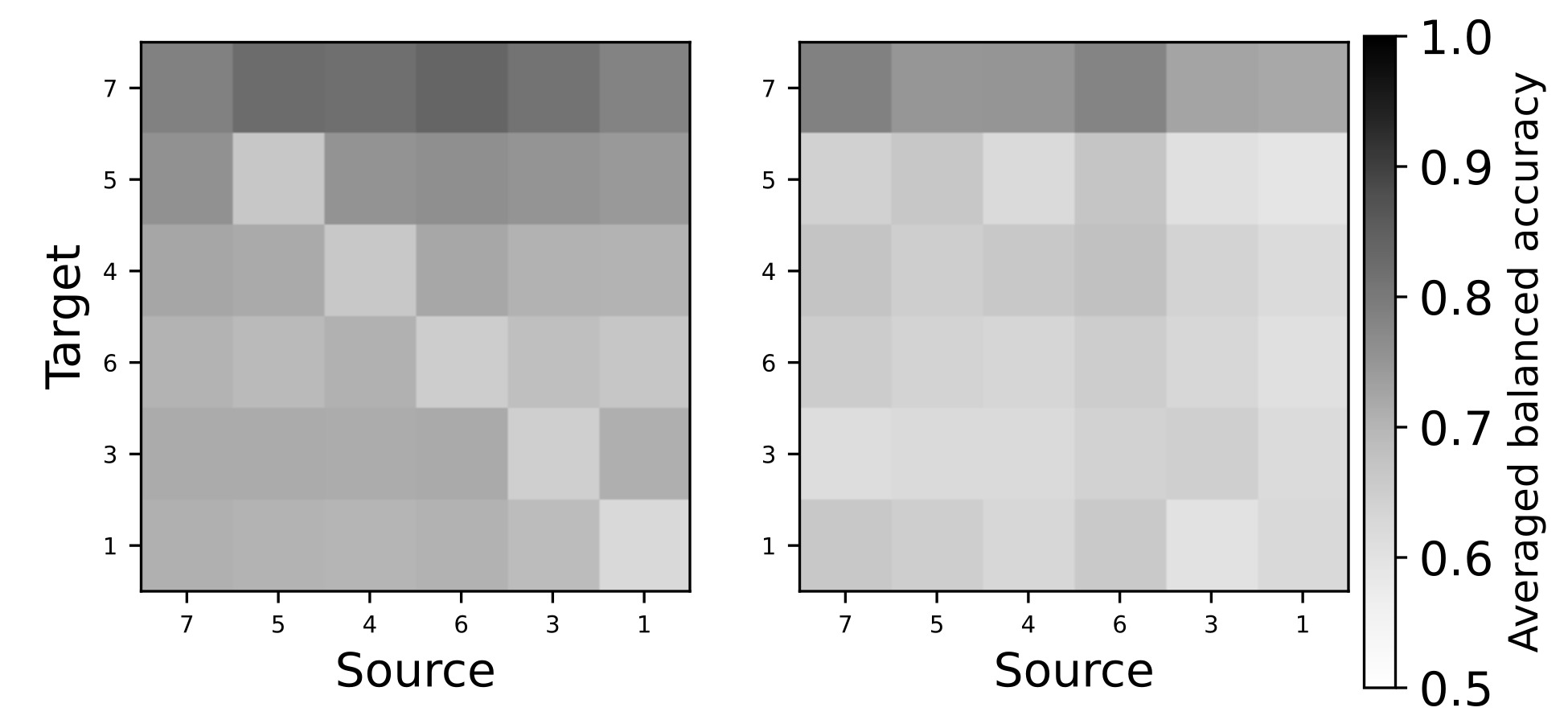

Supplement: Supplementary file 1 [file Data_Sheet_1.ZIP › ERP/seriation_both_EPFL_P300_dataset_pca3threshold0.60.jpg]

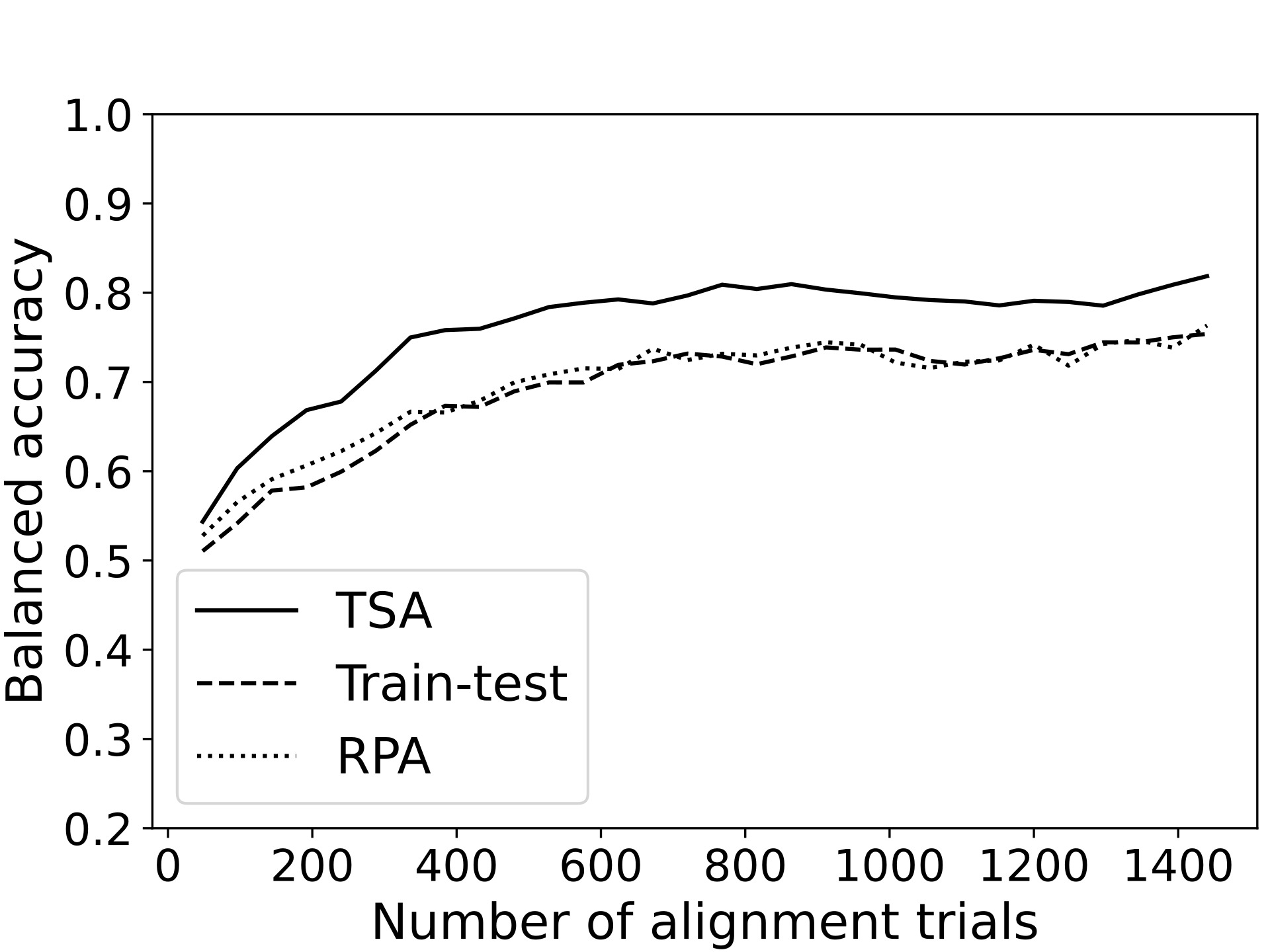

Supplement: Supplementary file 1 [file Data_Sheet_1.ZIP › ERP/accuracy_003-2015_pca3threshold0.60.jpg]

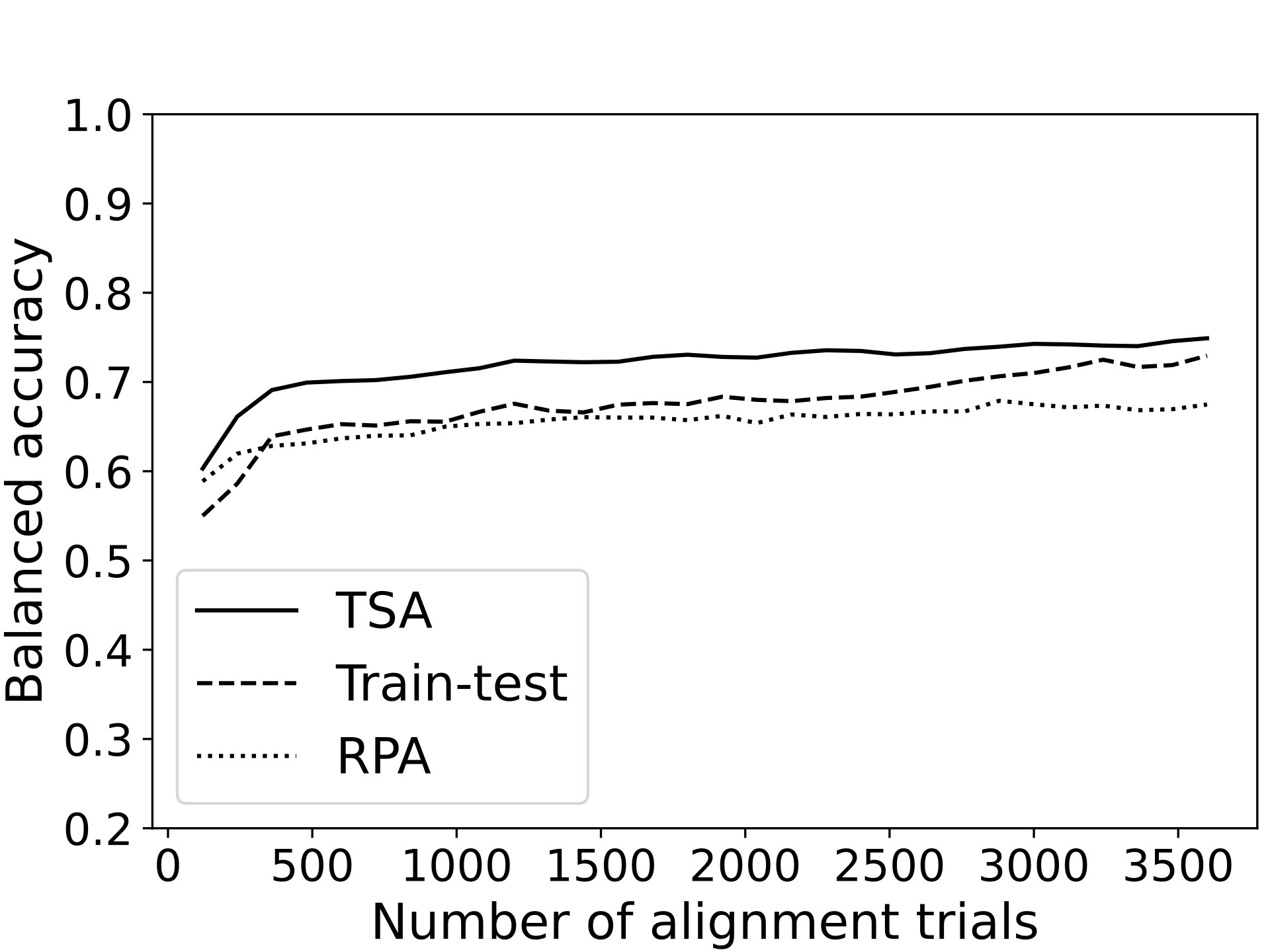

Supplement: Supplementary file 1 [file Data_Sheet_1.ZIP › ERP/accuracy_008-2014_pca3threshold0.60.jpg]

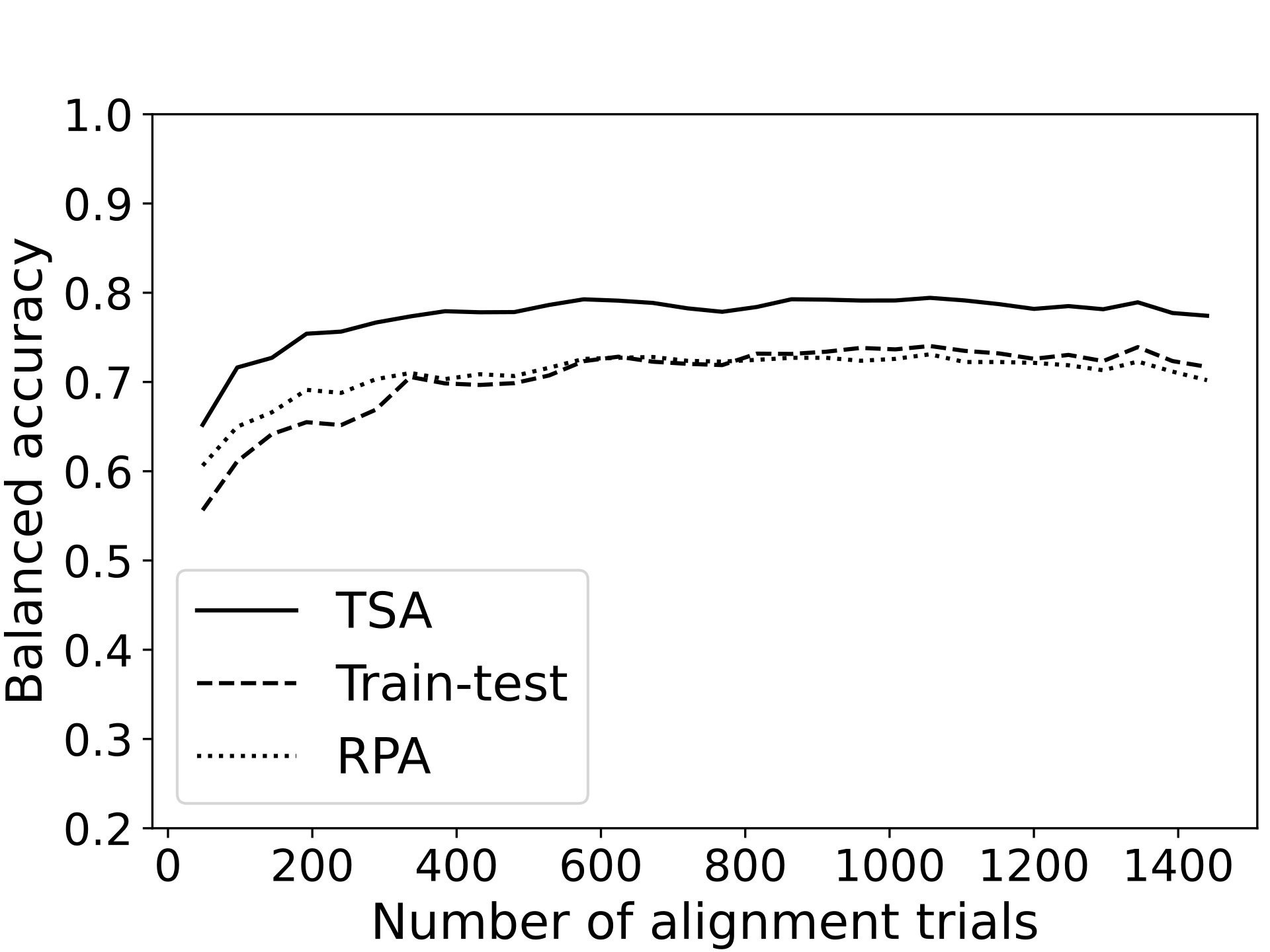

Supplement: Supplementary file 1 [file Data_Sheet_1.ZIP › ERP/accuracy_009-2014_pca3threshold0.60.jpg]

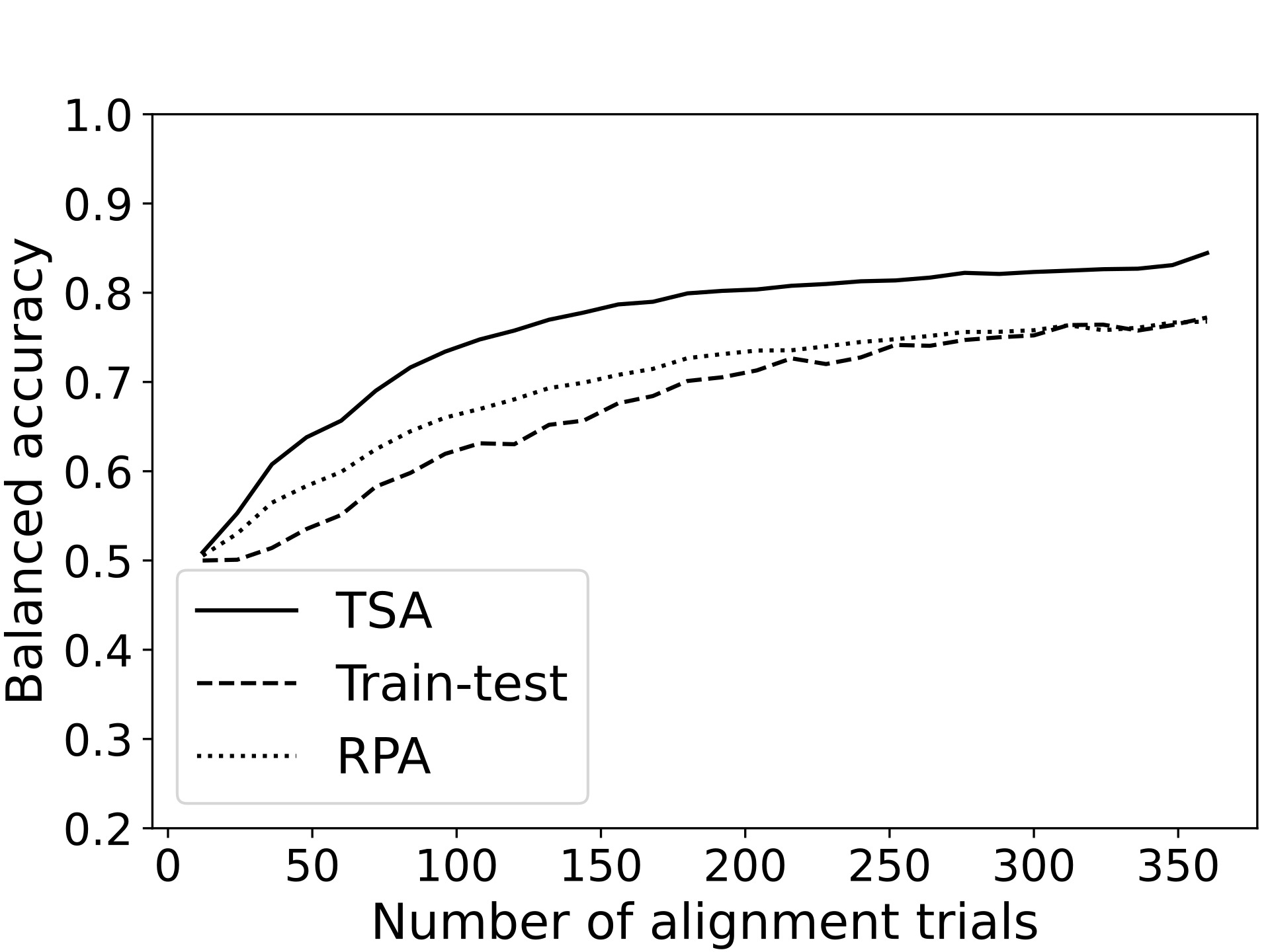

Supplement: Supplementary file 1 [file Data_Sheet_1.ZIP › ERP/accuracy_Brain_Invaders_2013a_pca3threshold0.60.jpg]

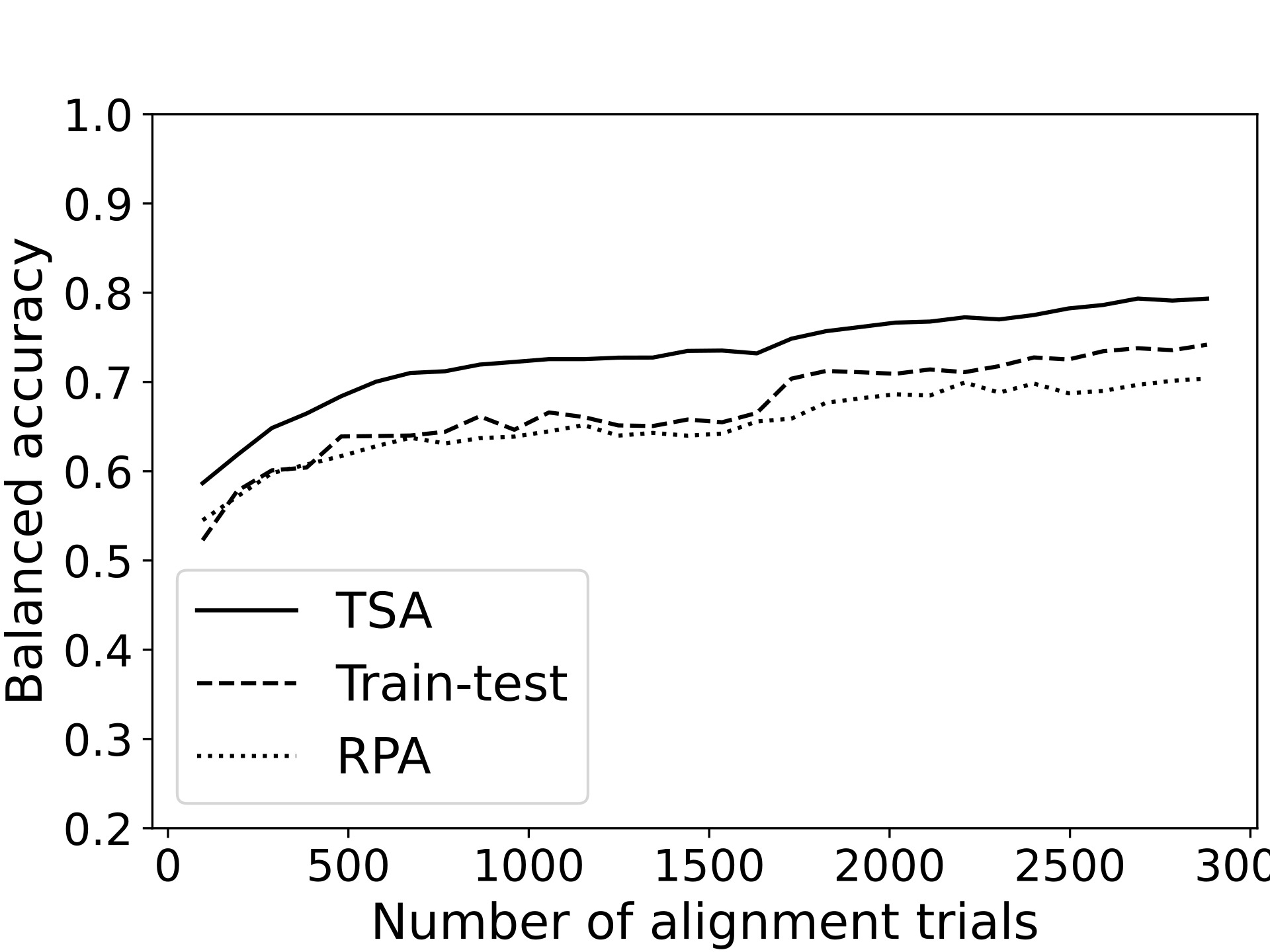

Supplement: Supplementary file 1 [file Data_Sheet_1.ZIP › ERP/accuracy_EPFL_P300_dataset_pca3threshold0.60.jpg]

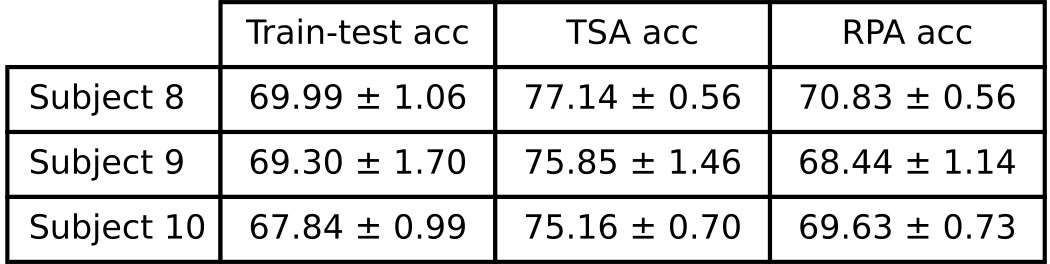

Supplement: Supplementary file 1 [file Data_Sheet_1.ZIP › ERP/accuracy_table_003-2015_pca3threshold0.60.jpg]

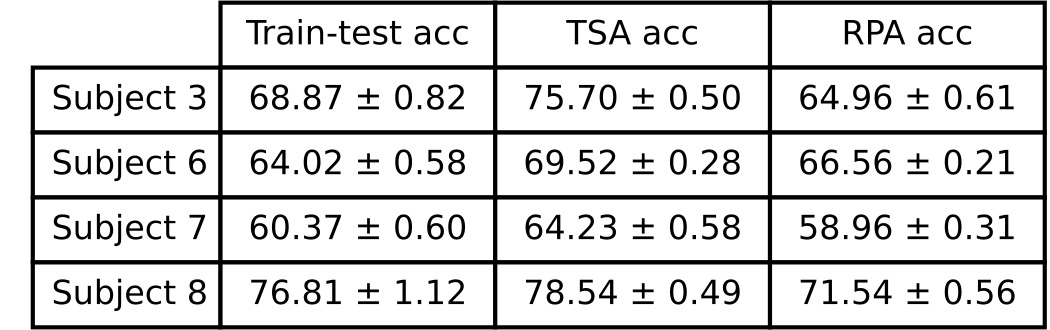

Supplement: Supplementary file 1 [file Data_Sheet_1.ZIP › ERP/accuracy_table_008-2014_pca3threshold0.60.jpg]

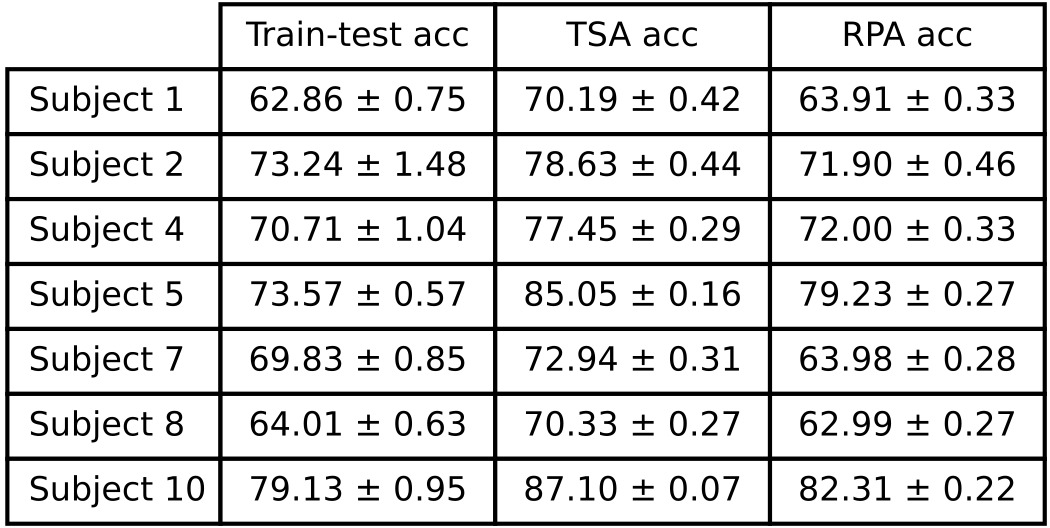

Supplement: Supplementary file 1 [file Data_Sheet_1.ZIP › ERP/accuracy_table_009-2014_pca3threshold0.60.jpg]

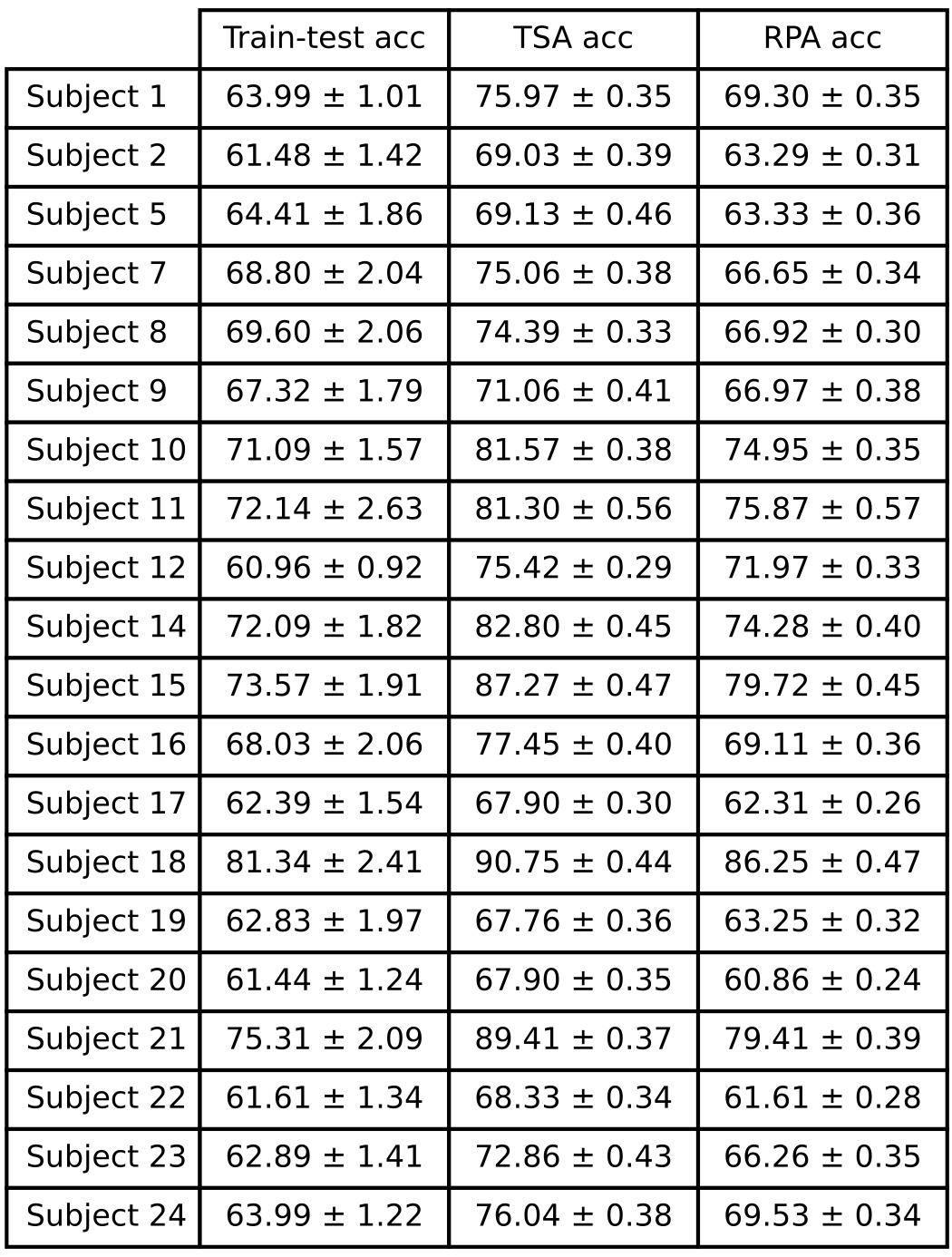

Supplement: Supplementary file 1 [file Data_Sheet_1.ZIP › ERP/accuracy_table_Brain_Invaders_2013a_pca3threshold0.60.jpg]

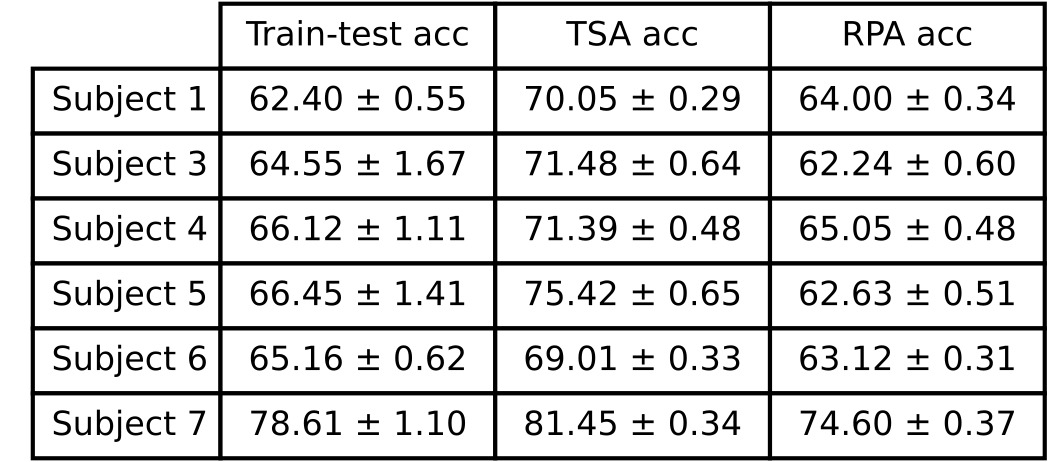

Supplement: Supplementary file 1 [file Data_Sheet_1.ZIP › ERP/accuracy_table_EPFL_P300_dataset_pca3threshold0.60.jpg]

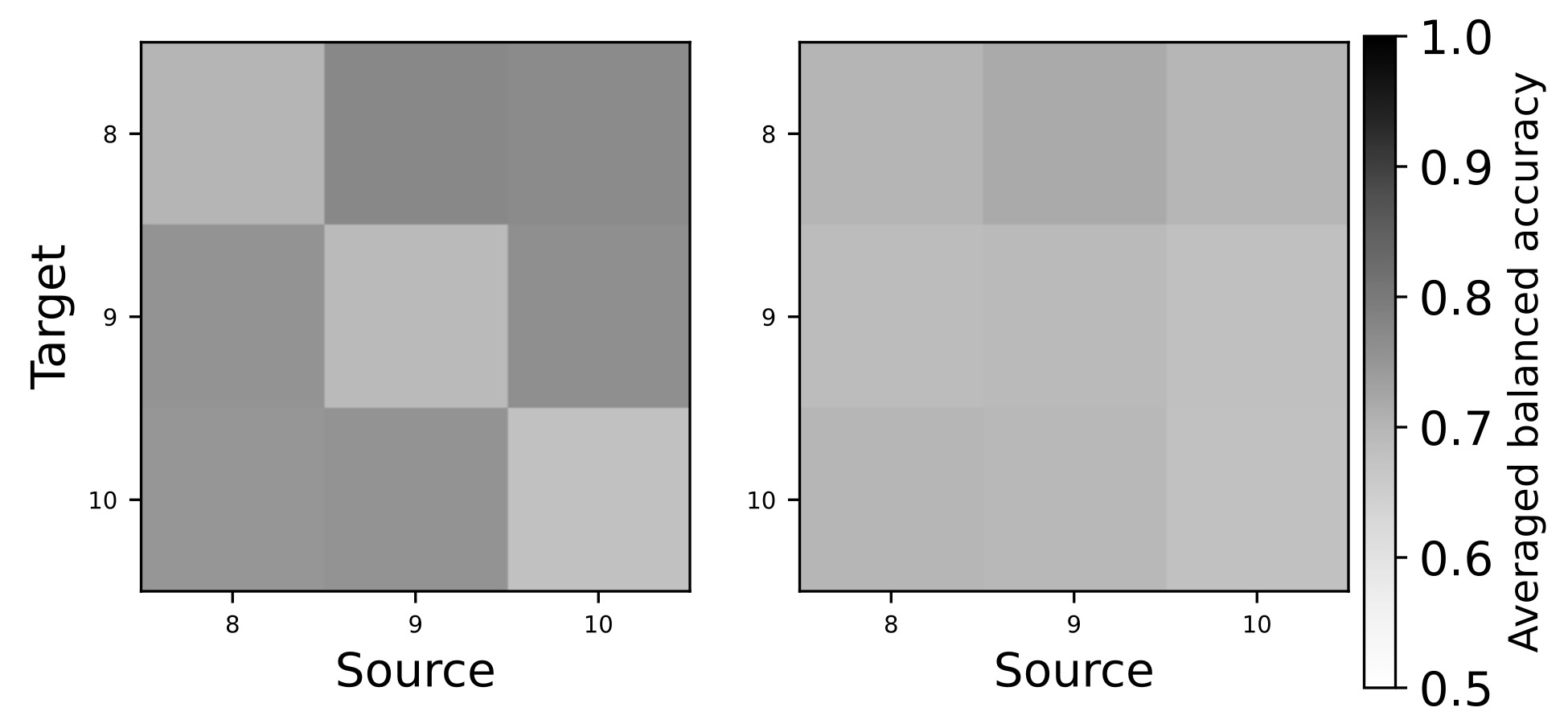

Supplement: Supplementary file 1 [file Data_Sheet_1.ZIP › ERP/seriation_both_003-2015_pca3threshold0.60.jpg]

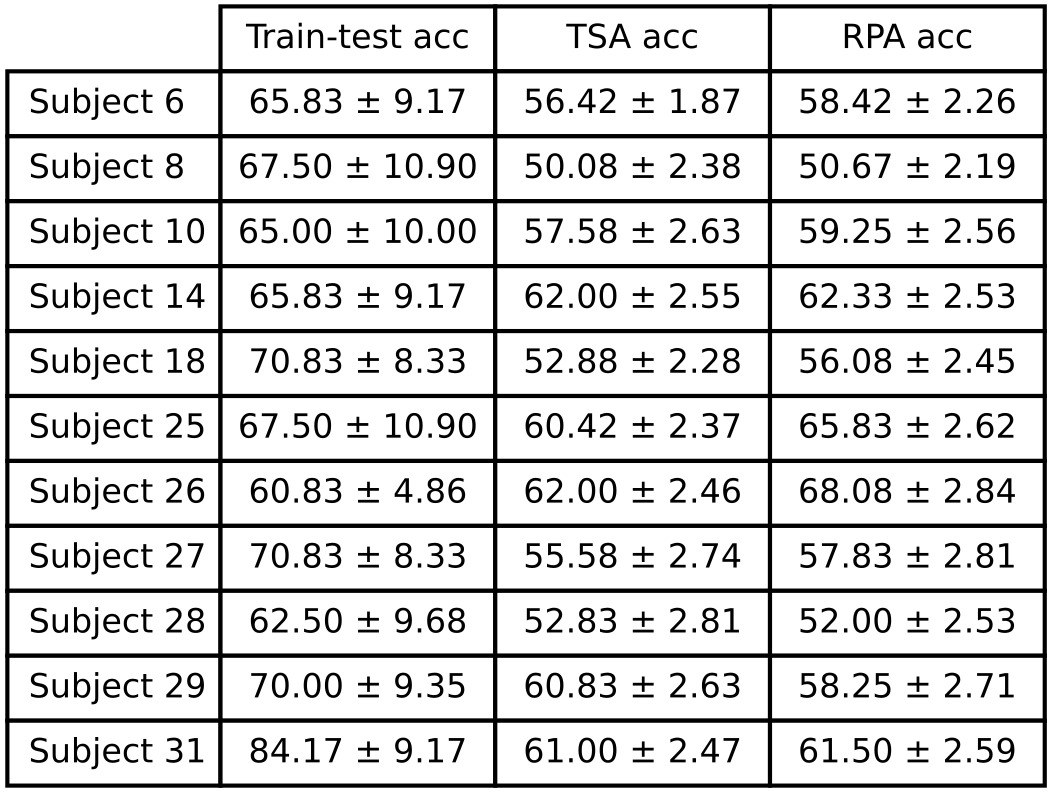

Supplement: Supplementary file 1 [file Data_Sheet_1.ZIP › SSVEP/accuracy_table_SSVEP_Wang_pca3threshold0.60.jpg]

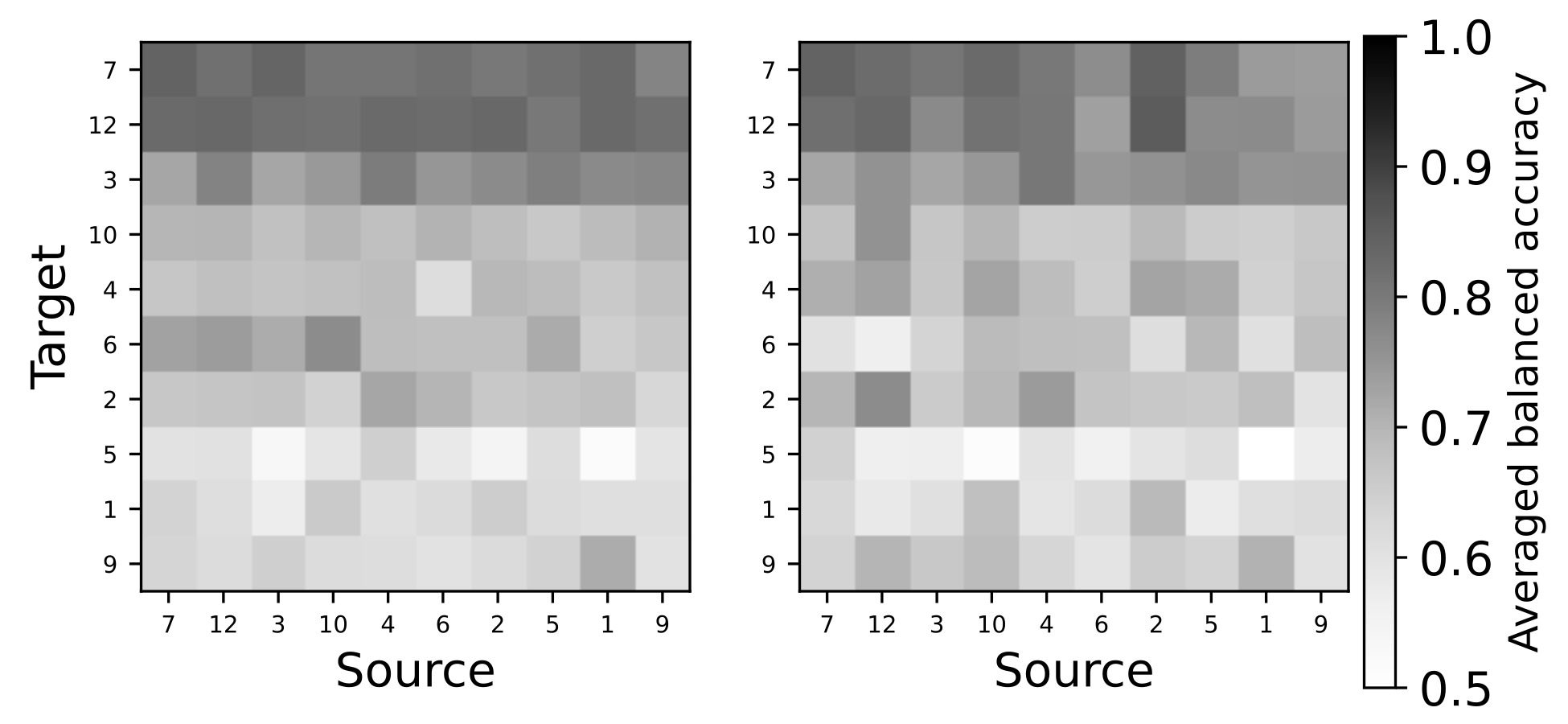

Supplement: Supplementary file 1 [file Data_Sheet_1.ZIP › SSVEP/seriation_both_SSVEP_Exoskeleton_pca3threshold0.60.jpg]

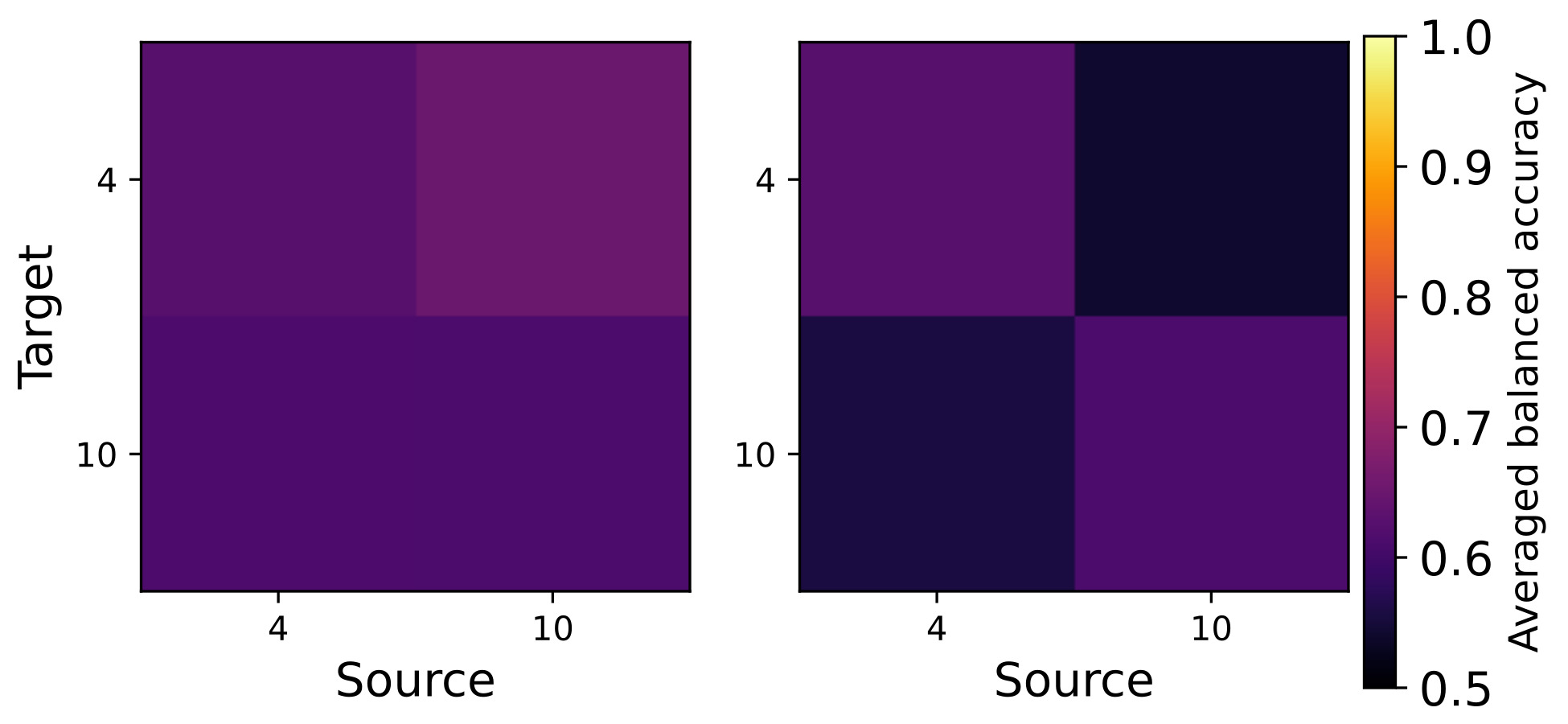

Supplement: Supplementary file 1 [file Data_Sheet_1.ZIP › SSVEP/seriation_both_SSVEP_MAMEM3_pca3threshold0.60.jpg]

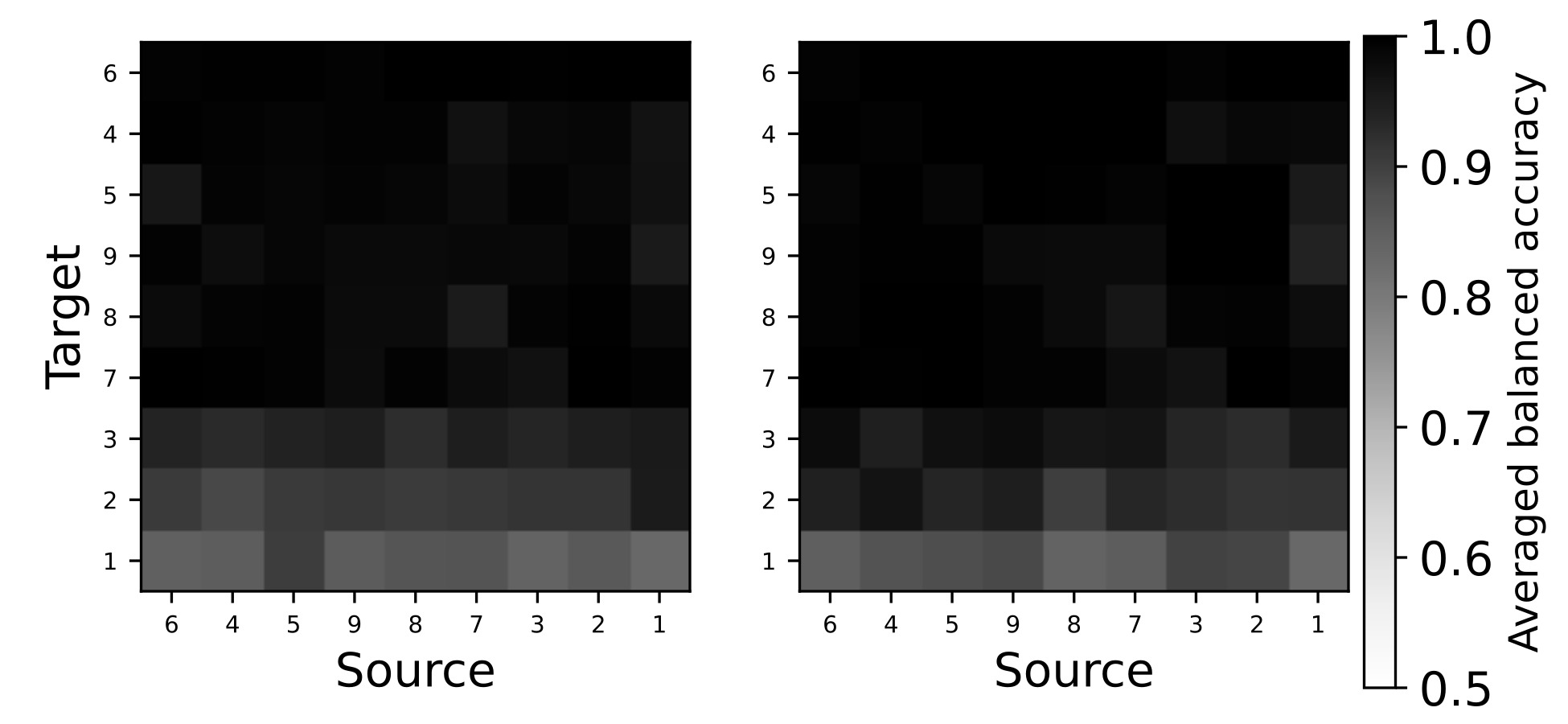

Supplement: Supplementary file 1 [file Data_Sheet_1.ZIP › SSVEP/seriation_both_SSVEP_Nakanishi_pca3threshold0.60.jpg]

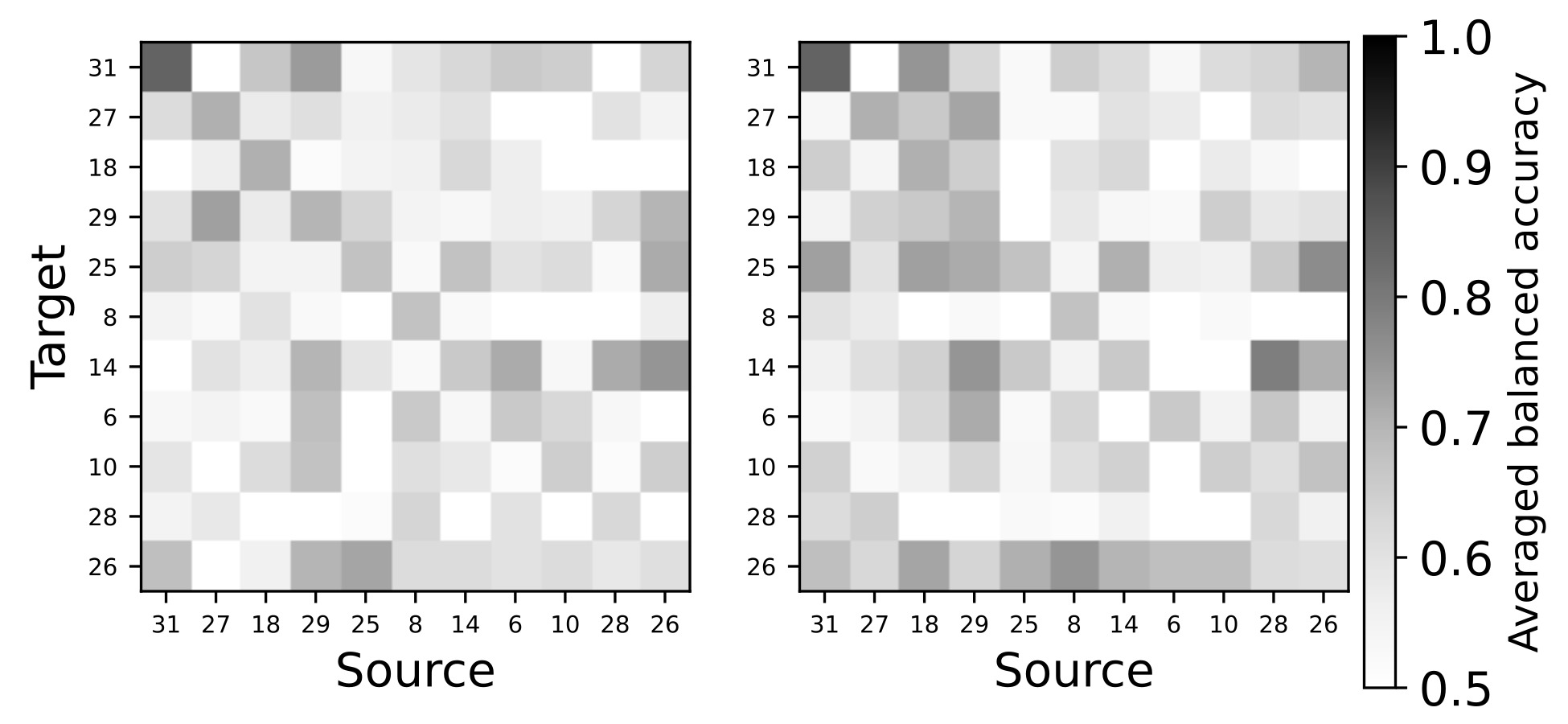

Supplement: Supplementary file 1 [file Data_Sheet_1.ZIP › SSVEP/seriation_both_SSVEP_Wang_pca3threshold0.60.jpg]

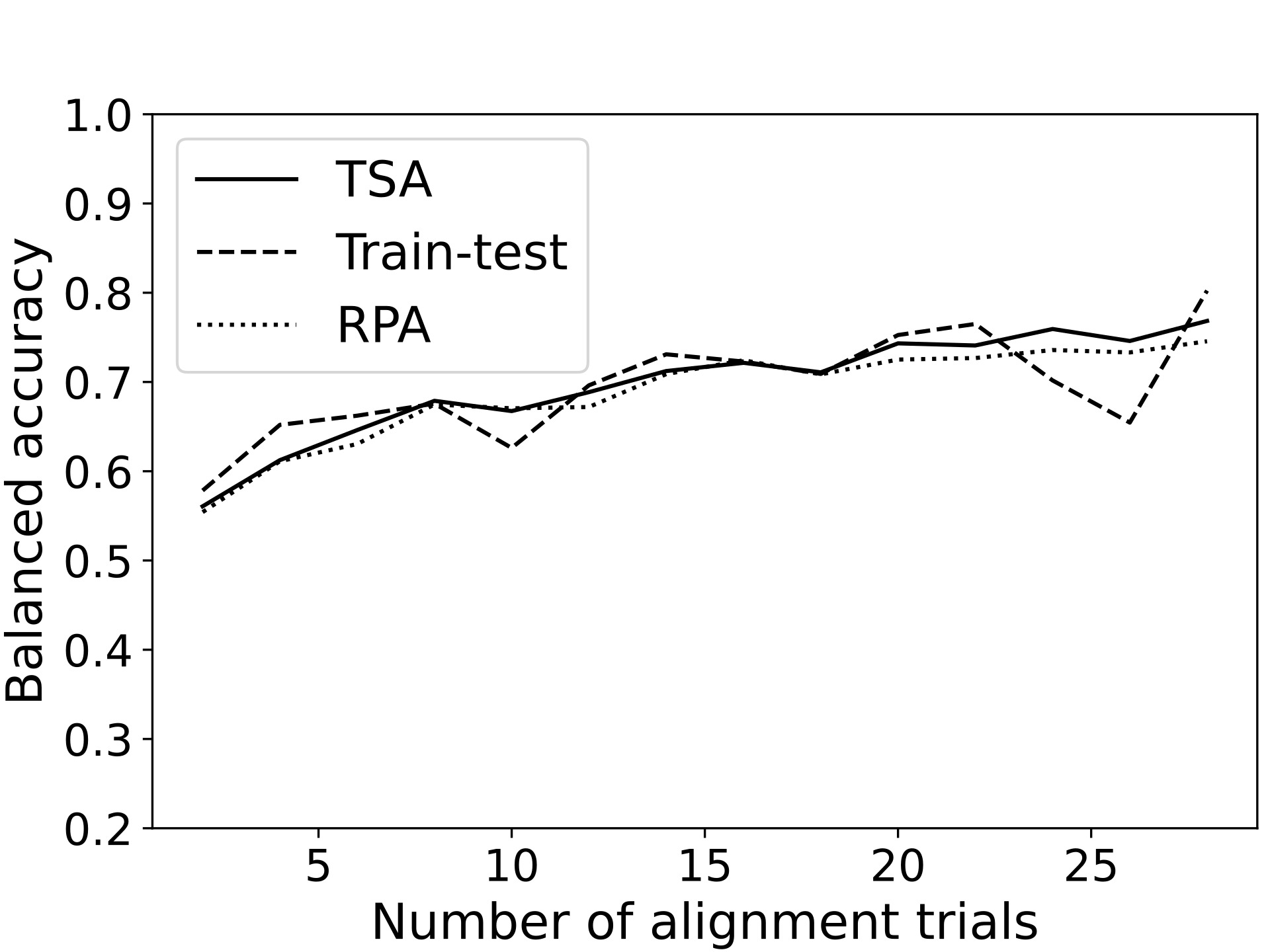

Supplement: Supplementary file 1 [file Data_Sheet_1.ZIP › SSVEP/accuracy_SSVEP_Exoskeleton_pca3threshold0.60.jpg]

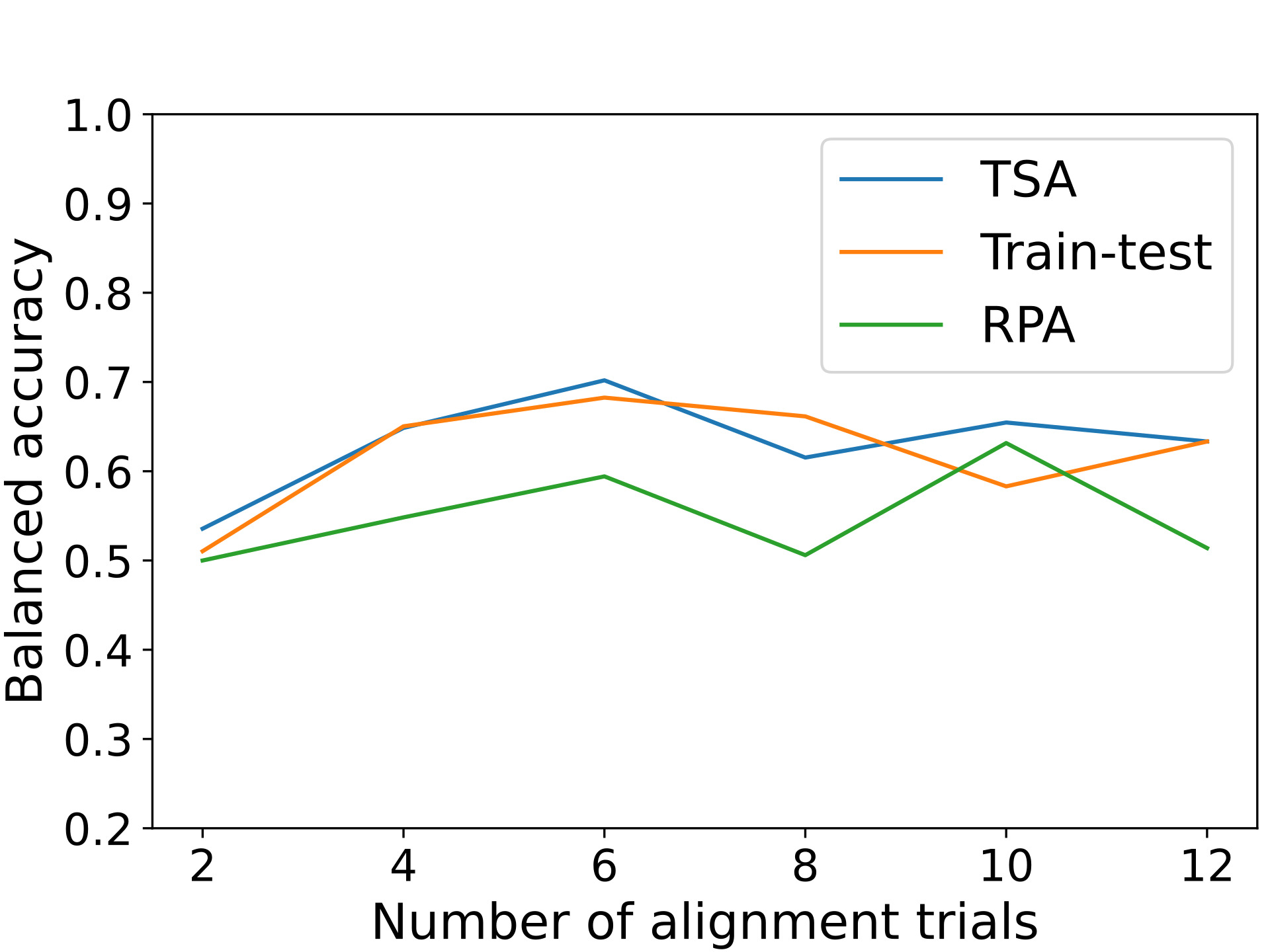

Supplement: Supplementary file 1 [file Data_Sheet_1.ZIP › SSVEP/accuracy_SSVEP_MAMEM3_pca3threshold0.60.jpg]

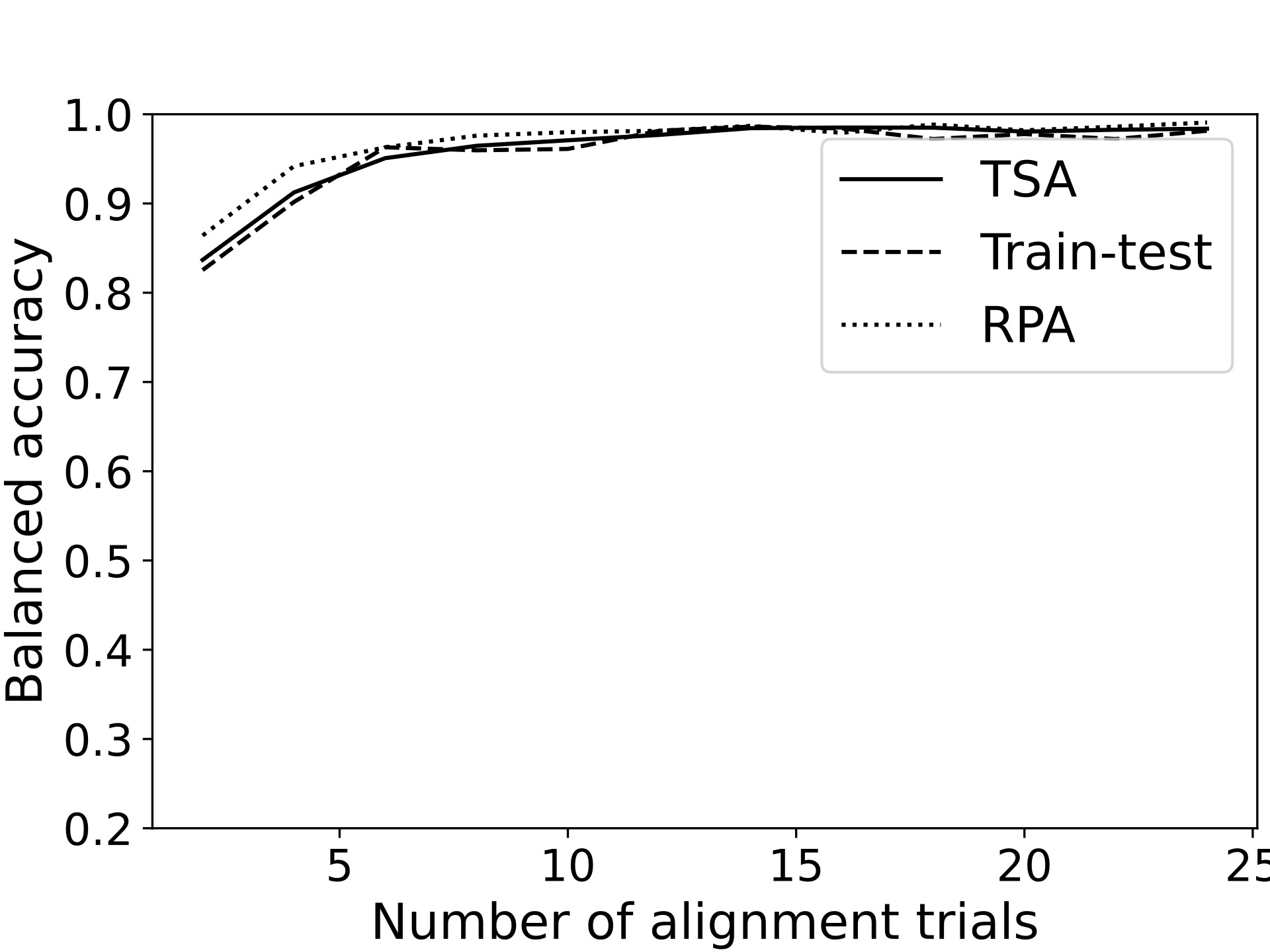

Supplement: Supplementary file 1 [file Data_Sheet_1.ZIP › SSVEP/accuracy_SSVEP_Nakanishi_pca3threshold0.60.jpg]

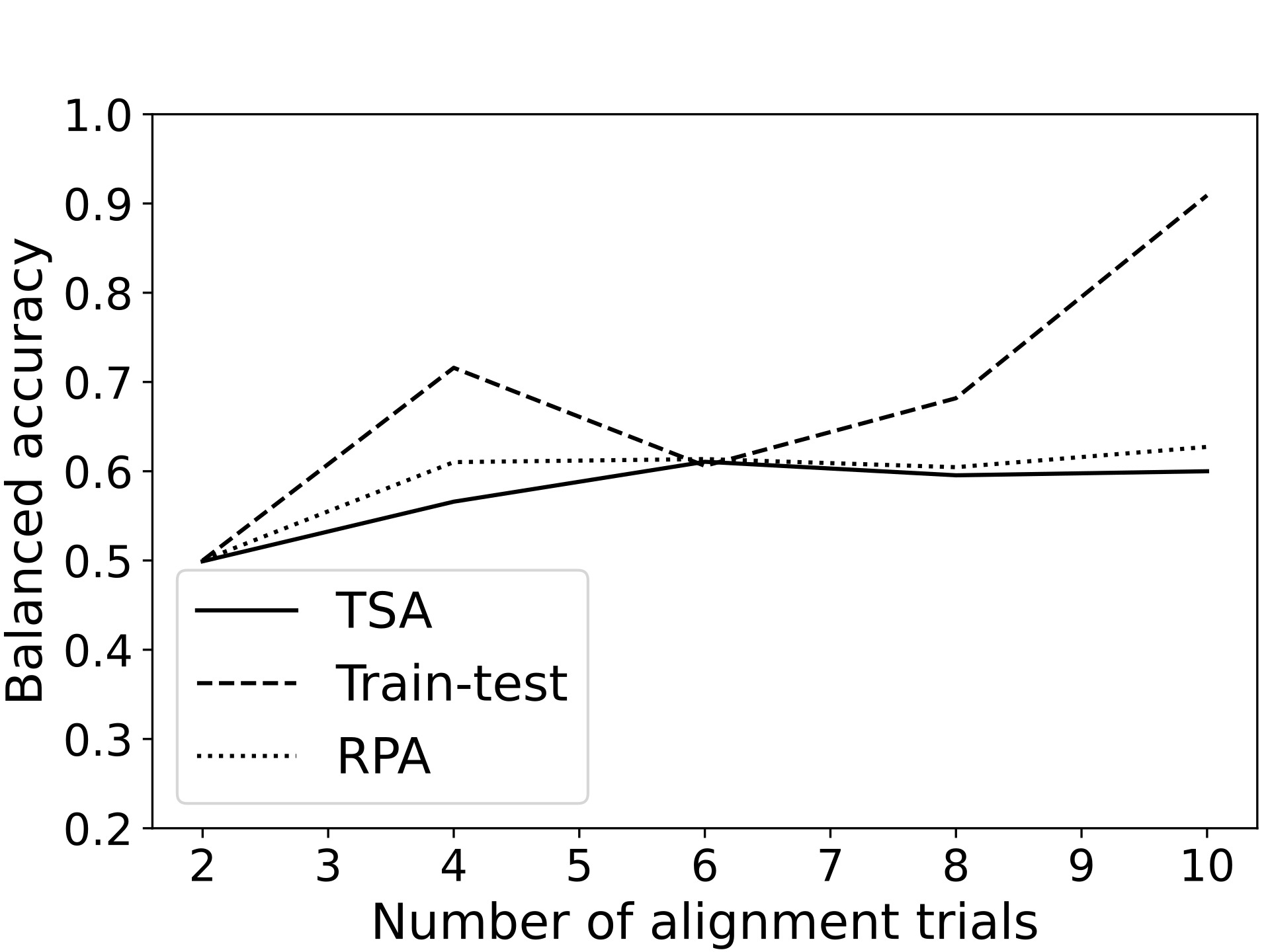

Supplement: Supplementary file 1 [file Data_Sheet_1.ZIP › SSVEP/accuracy_SSVEP_Wang_pca3threshold0.60.jpg]

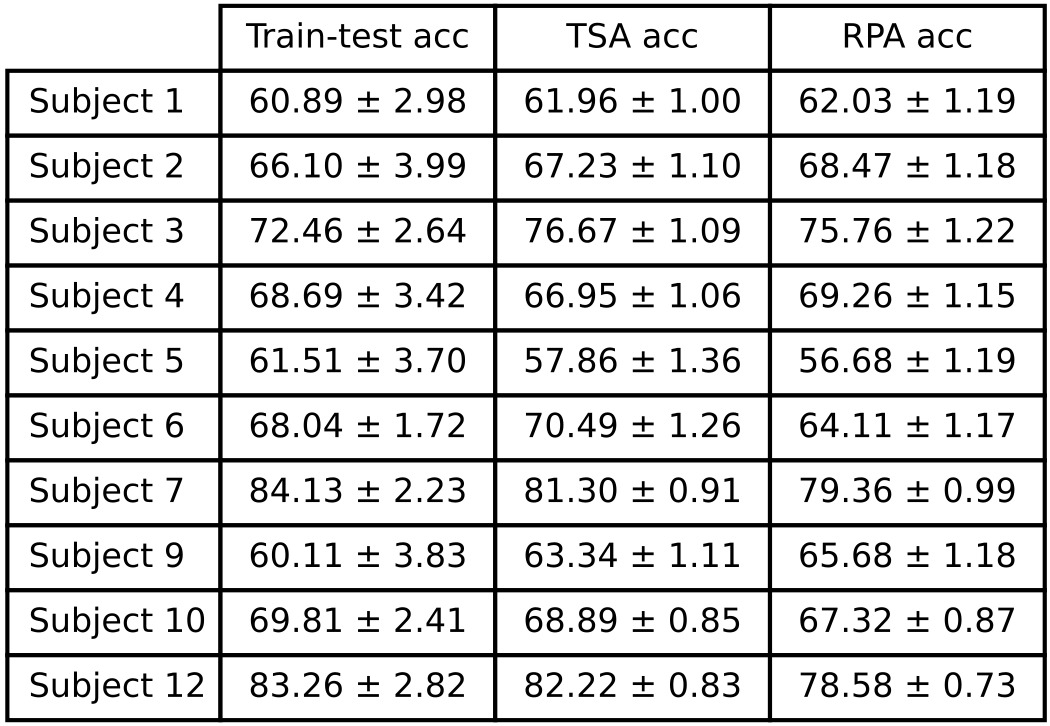

Supplement: Supplementary file 1 [file Data_Sheet_1.ZIP › SSVEP/accuracy_table_SSVEP_Exoskeleton_pca3threshold0.60.jpg]

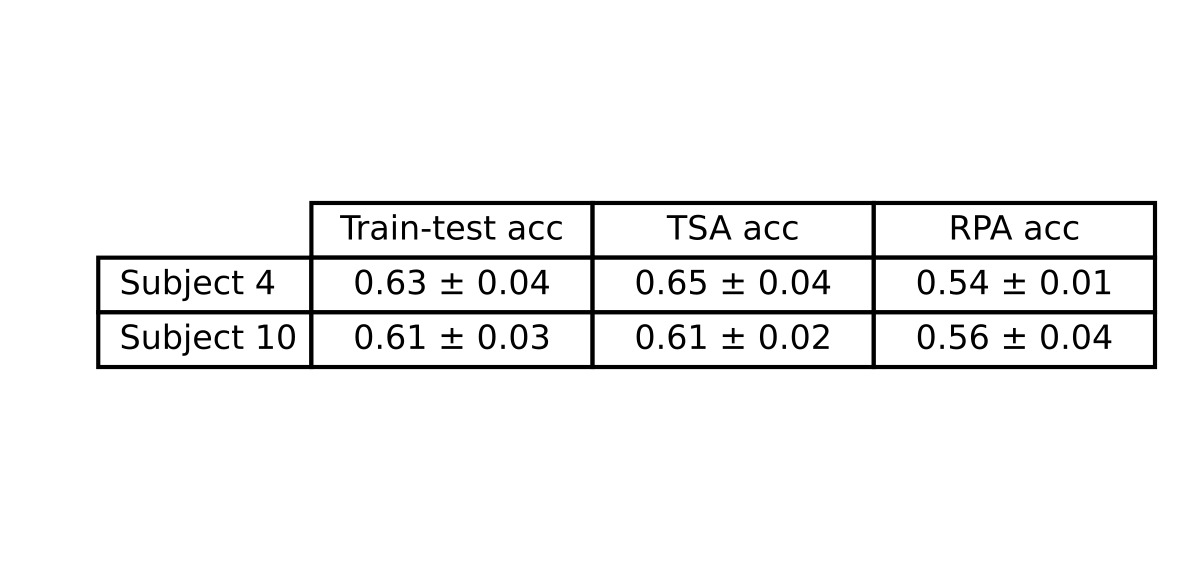

Supplement: Supplementary file 1 [file Data_Sheet_1.ZIP › SSVEP/accuracy_table_SSVEP_MAMEM3_pca3threshold0.60.jpg]

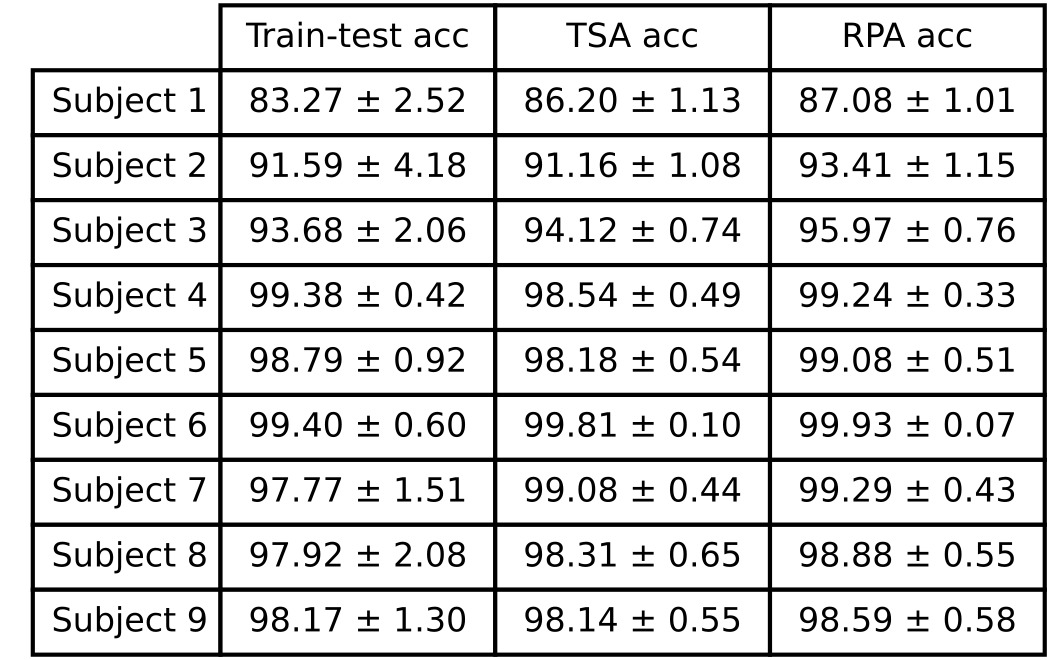

Supplement: Supplementary file 1 [file Data_Sheet_1.ZIP › SSVEP/accuracy_table_SSVEP_Nakanishi_pca3threshold0.60.jpg]

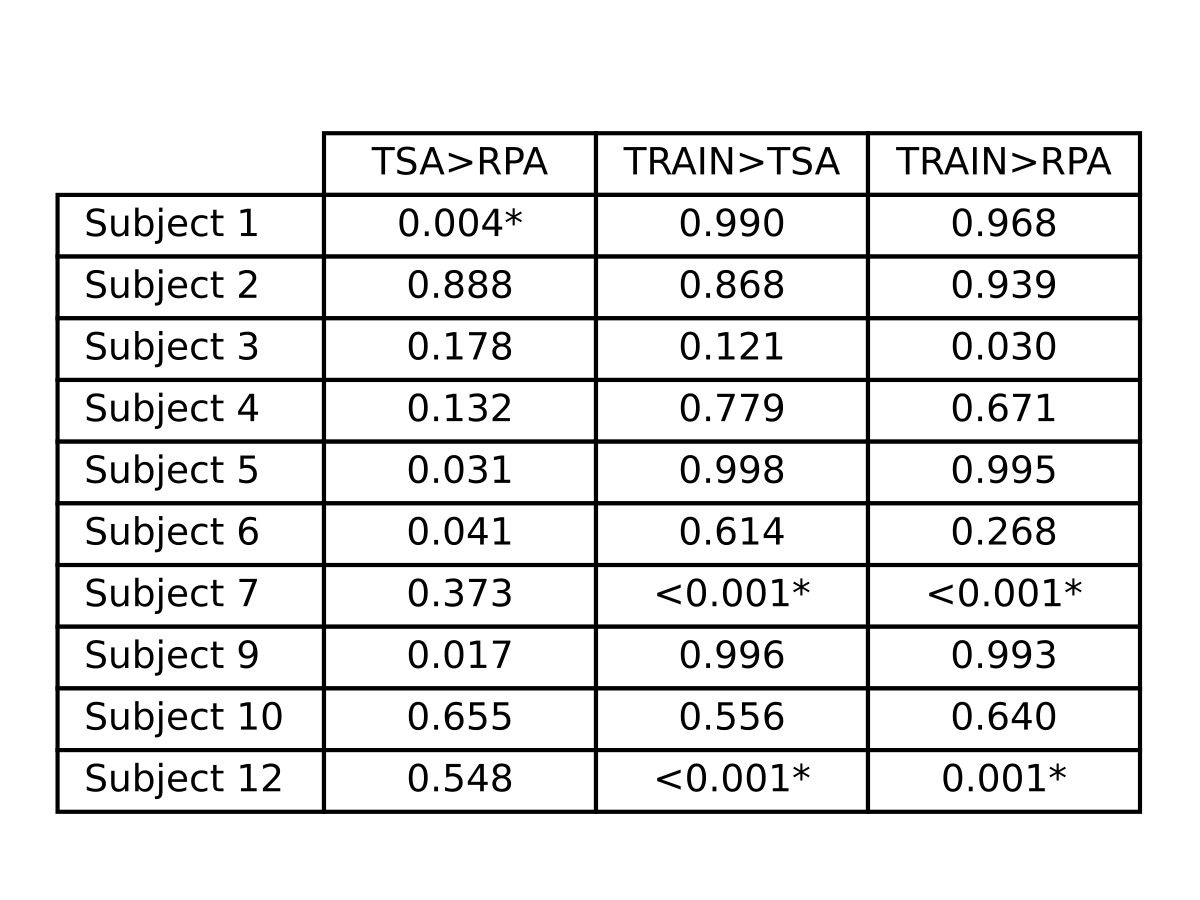

Supplement: Supplementary file 1 [file Data_Sheet_1.ZIP › SSVEP/SSVEP_Exoskeleton_threshold_0.60.jpg]

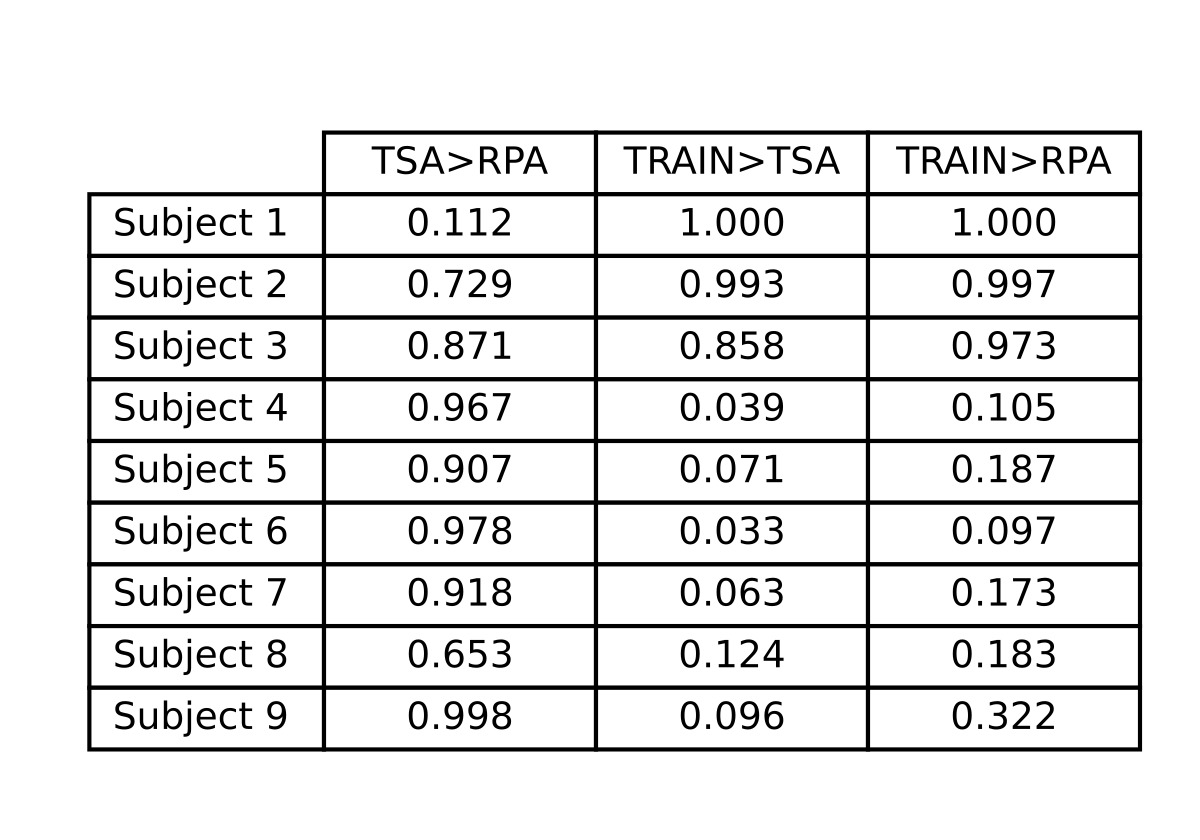

Supplement: Supplementary file 1 [file Data_Sheet_1.ZIP › SSVEP/SSVEP_Nakanishi_threshold_0.60.jpg]

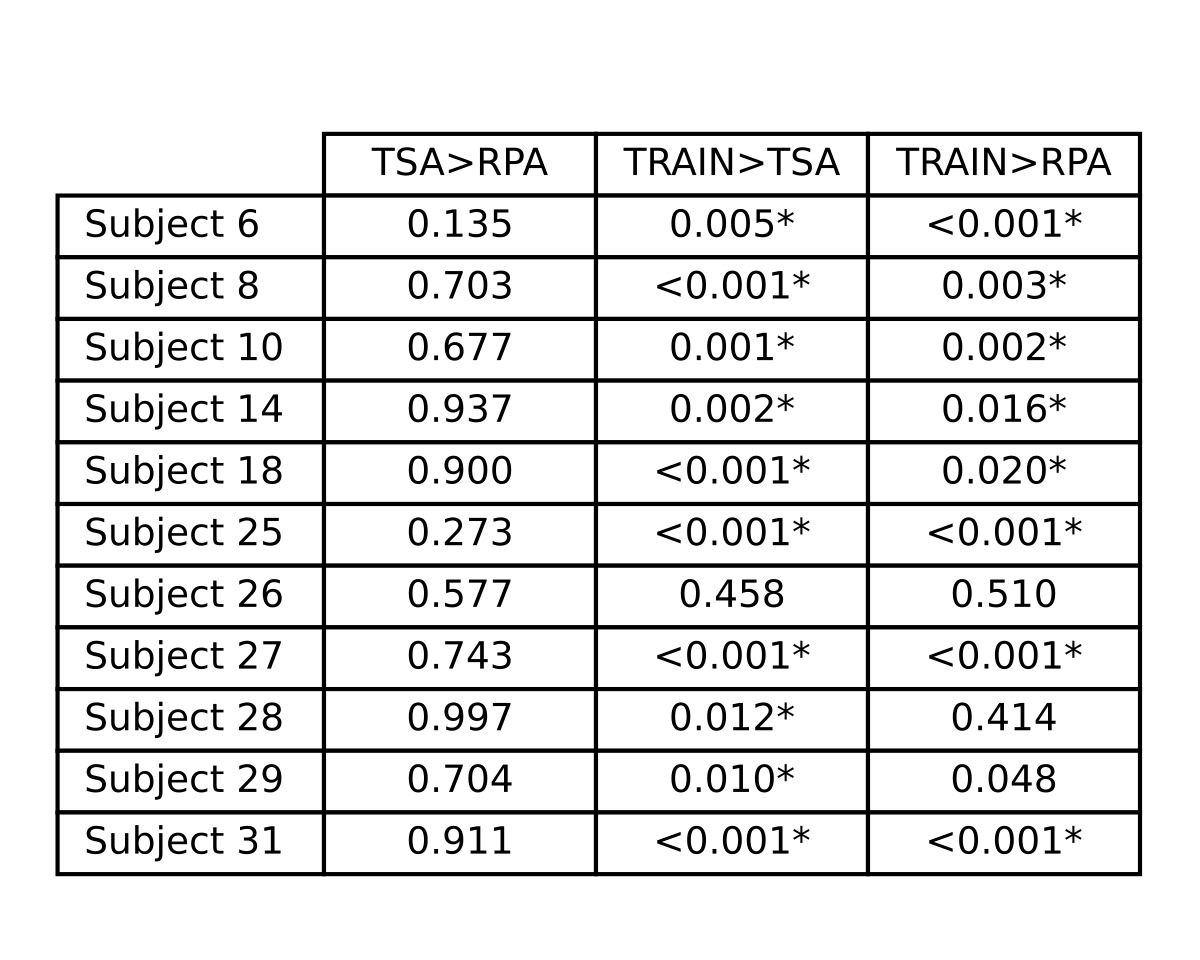

Supplement: Supplementary file 1 [file Data_Sheet_1.ZIP › SSVEP/SSVEP_Wang_threshold_0.60.jpg]
